# A High-Density Linkage Map for *Astyanax mexicanus* Using Genotyping-by-Sequencing Technology

**DOI:** 10.1534/g3.114.015438

**Published:** 2014-12-17

**Authors:** Brian M. Carlson, Samuel W. Onusko, Joshua B. Gross

**Affiliations:** Department of Biological Sciences, University of Cincinnati, Cincinnati, Ohio 45221

**Keywords:** next-generation sequencing, QTL analysis, blind Mexican cave tetra, regressive phenotypic evolution

## Abstract

The Mexican tetra, *Astyanax mexicanus*, is a unique model system consisting of cave-adapted and surface-dwelling morphotypes that diverged >1 million years (My) ago. This remarkable natural experiment has enabled powerful genetic analyses of cave adaptation. Here, we describe the application of next-generation sequencing technology to the creation of a high-density linkage map. Our map comprises more than 2200 markers populating 25 linkage groups constructed from genotypic data generated from a single genotyping-by-sequencing project. We leveraged emergent genomic and transcriptomic resources to anchor hundreds of anonymous *Astyanax* markers to the genome of the zebrafish (*Danio rerio*), the most closely related model organism to our study species. This facilitated the identification of 784 distinct connections between our linkage map and the *Danio rerio* genome, highlighting several regions of conserved genomic architecture between the two species despite ∼150 My of divergence. Using a Mendelian cave-associated trait as a proof-of-principle, we successfully recovered the genomic position of the albinism locus near the gene *Oca2*. Further, our map successfully informed the positions of unplaced *Astyanax* genomic scaffolds within particular linkage groups. This ability to identify the relative location, orientation, and linear order of unaligned genomic scaffolds will facilitate ongoing efforts to improve on the current early draft and assemble future versions of the *Astyanax* physical genome. Moreover, this improved linkage map will enable higher-resolution genetic analyses and catalyze the discovery of the genetic basis for cave-associated phenotypes.

The blind Mexican cave tetra is a powerful system for understanding the evolutionary mechanisms governing regressive phenotypes. These animals were discovered in 1936 and initially were assigned to a new genus—*Anoptichthys* (“bony fish without eyes”) (Hubbs and Innes 1936). Breeding studies in the 1940s led to the discovery of viable hybrid offspring resulting from crosses between the (derived) blind cave-dwelling forms and (ancestral) surface-dwelling forms from the same geographical region of northeast Mexico (Breder 1943a, b). Both morphotypes are now regarded as members of the same (or a closely related) species, *Astyanax mexicanus*. This system has spurred more than half a century of comparative research (Şadoǧlu 1956) focusing on unresolved problems in evolution (Jeffery 2001), development (Pottin *et al.* 2011), genetics (Schemmel 1980), physiology (Salin *et al.* 2010), and behavior (Burchards *et al.* 1985).

Classical and quantitative genetic approaches have provided clear evidence that many troglomorphic (cave-associated) phenotypes evolved through heritable genetic changes. These studies centered on both Mendelian and complex phenotypes, including eye regression (Yamamoto *et al.* 2004; Protas *et al.* 2007; Yoshizawa *et al.* 2012; O’Quin *et al.* 2013), feeding-related behaviors (Schemmel 1980; Yoshizawa *et al.* 2012), sleep loss (Duboué *et al.* 2011), schooling behavior (Kowalko *et al.* 2013), pigmentation loss (reviewed in Jeffery 2009), and intraspecific aggression (Elipot *et al.* 2013). QTL studies have identified candidate genes mediating a variety of these traits, such as retinal degeneration (O’Quin *et al.* 2013), rib number, eye size (Gross *et al.* 2008), albinism (*Oca2*) (Protas *et al.* 2006), and the *brown* phenotype (*Mc1r*) (Gross *et al.* 2009).

Genomic resources for this model system, however, have historically been limited. The first linkage map was calculated based on recombination frequencies of an experimental F_1_ × Pachón cave backcross pedigree using markers generated from random amplified polymorphic DNA (RAPD) fingerprinting (Borowsky and Wilkens 2002). This map was supplanted by a higher-resolution map using more individuals and markers composed of polymorphic microsatellites identified using CA_N_ dinucleotide repeats (Protas *et al.* 2006). Using this second-generation linkage map, Protas *et al.* (2008) discovered a genetic basis for several cave-associated phenotypic changes including pigmentation regression, reduced rib numbers, slower weight loss, and increased chemical sensitivity. Early comparative genomic analyses utilizing this map first demonstrated extensive synteny conserved between *Astyanax* and *Danio rerio*, despite ∼150 My of divergence (Gross *et al.* 2008). The first next-generation sequencing (NGS)-based linkage map using restriction-associated DNA sequencing (RAD-seq) technology was published by O’Quin *et al.* (2013). This map, comprising 698 markers on 25 linkage groups, strengthened the evidence for vast regions of synteny between the genomes of *Astyanax* and zebrafish and identified several critical loci associated with retinal degeneration (O’Quin *et al.* 2013).

Here, we present the most dense, comprehensive linkage map to date using genotyping-by-sequencing (GBS) technology. This technology enables accurate and high-throughput collection of massive amounts of sequence data (Davey *et al.* 2011), including thousands of single-nucleotide polymorphisms (SNPs) segregating between cave-dwelling and surface-dwelling morphs. GBS utilizes deep Illumina sequencing of restriction enzyme-nicked genomic DNA libraries that are uniquely barcoded for each member of an experimental pedigree. This technique is optimized to avoid inclusion of repetitive portions of the genome and is extremely specific and highly reproducible (Elshire *et al.* 2011). Fish are well-represented among studies using GBS and other RAD-seq–based methodologies (Rowe *et al.* 2011). However, a majority of GBS studies in fish have focused on species of commercial (Everett *et al.* 2012; Houston *et al.* 2012; Li *et al.* 2014) or conservational concern (Hecht *et al.* 2013; Ogden *et al.* 2013; Hess *et al.* 2014; Larson *et al.* 2014). Here, we adapted this technology to construct a high-density linkage map for evolutionary and developmental studies in our emerging model system. The resulting linkage map will enable higher-resolution genomic studies and inform the assignment of chromosomal builds for the ongoing *Astyanax* genome sequencing project (McGaugh *et al.* 2014).

## Materials and Methods

### Pedigree, husbandry, and genomic DNA isolation

Linkage mapping and QTL studies were performed using genotypic and phenotypic data obtained from two separate F_2_ hybrid mapping populations (n = 129; n = 41) bred from a male surface fish and female cavefish from the Pachón cave. In addition, surface (n = 4), Pachón cave (n = 4), and surface × Pachón F_1_ hybrid (n = 4) specimens were used to evaluate and code GBS markers for use with JoinMap software (v. 4.1; Kyazma; see below), but were not included in linkage mapping calculations. Parental specimens belonged to laboratory populations originally sourced from the El Abra region of northeastern Mexico and all fish used were generously provided to our laboratory by Dr. Richard Borowsky (New York University). All live fish used in this study were maintained as previously described (see Gross *et al.* 2013). Every individual from the “Asty66” F_2_ population (n = 129) was individually reared in a 1-liter tank. All phenotypic data from the “Asty12” F_2_ population (n = 41) were obtained from paraformaldehyde-preserved specimens.

### Genotyping-by-sequencing

Genomic DNA was extracted from caudal tail fin tissue of live surface, cave, and F_1_ and F_2_ hybrid *Astyanax mexicanus* specimens using the DNeasy Blood and Tissue Kit (Qiagen) as previously described (Gross *et al.* 2013). Twenty genomic samples were digested with *Eco*RI, subjected to gel electrophoresis and imaged to verify that sample quality, concentration, and restriction fragment size distributions were suitable for use in downstream analyses. DNA samples were then pipetted into individual wells of 96-well plates and diluted to a final volume of 30 µl (100 ng/µl). Samples were processed by the Institute for Genomic Diversity (Cornell University), where genomic libraries were constructed and GBS was performed as described elsewhere (Elshire *et al.* 2011; Lu *et al.* 2013).

### GBS marker selection

Genotypes for each of 7956 GBS markers (each consisting of a single SNP in a 64-bp-long sequence fragment) were screened in cave and surface (parental) forms to assign the morphotypic origin of each allele. F_1_ individuals were then evaluated to confirm heterozygosity at each locus. The morphotypic origin of each allele was assigned by consensus—if three or more (out of four) surface or cave individuals had the identical nucleotide at a particular locus, then the genotype was assigned to the consensus parental population. Likewise, a true “hybrid” genotype was assigned if three or more F_1_ individuals harbored the same heterozygous condition (*e.g.*, M, R, S, W, Y, K SNP code) at a given locus. Those genotypes with an ambiguous morphotypic origin were denoted “NA.”

Markers were then screened for suitability in linkage calculations. Markers were deemed unsuitable and discarded from further analysis if neither parental genotype could be assigned (*i.e.*, both the surface and cave genotypes were scored “NA”) or if the assigned surface and cave genotypes were identical; 6006 genomic markers were deemed suitable and prepared for linkage map calculation using the “cross-pollination” (CP) segregation coding used in JoinMap. At this stage, 107 markers were found to be uninformative (*i.e.*, a single genotype was shared by all F_2_ individuals) and discarded from further analysis. We screened the remaining set (n = 5899) to identify markers failing to conform to predicted genotypic ratios (*e.g.*, 1:2:1 ratios across the entire pedigree); 2896 markers demonstrated a χ^2^ value more than 50, implying significant departure from the predicted genotype ratio and were discarded from further analysis. Our final GBS marker set included 3003 markers evaluated in 170 F_2_ individuals.

### Linkage map construction and QTL analysis

Linkage map calculations were performed using JoinMap (v.4.1, Kyazma). Our workflow used program default settings, with the following exceptions: the maximum grouping independence LOD value was set to 50.0; linkage groups were calculated using regression mapping; and linkage mapping was performed using the Kosambi method (Kosambi 1943). Linkage groups were assigned based on independence LOD scores. We increased the maximum grouping independence LOD value to 50.0, because the default value of 10.0 did not allow sufficient subdivision of our data into an appropriate number of groups. Initial groupings identified 29 groups populated with between 10 and 225 markers, with independence LOD scores ranging from 7.0 to 21.0. These groups were then processed for formal mapping calculations.

The first round of mapping produced 28 linkage groups comprising a total map length of 2956 cM. At this stage, one linkage group (comprising 10 markers, independence LOD = 19.0) failed to assemble into a consolidated group and was therefore eliminated from further analysis. The remaining individual linkage groups ranged in length from 27.25 to 187.46 cM, containing between 10 and 225 markers with an average intermarker distance between 0.51 and 6.40 cM. After this initial round of mapping, we further screened existing linkages to target the most optimal 25 groups (*Astyanax mexicanus* has karyotypic number of 25; Kirby *et al.* 1977) and reduce the average intermarker distance to a target of ∼1 cM. Accordingly, nine groups (10 ≤ n ≤ 45 markers) were removed because of low marker number and/or unusually high average intermarker distance. The five largest groups (154 ≤ n ≤ 225 markers) were then subdivided at the lowest independence LOD value resulting in two linkage groups comprising 20 or more markers. Throughout mapping, we limited the inflation of the overall map length by eliminating certain markers sparsely populating distal ends of otherwise densely populated linkage groups. This resulted in size reduction of the five longest remaining linkage groups (142.041 ≤ n ≤ 187.458 cM) by splitting them at the lowest independence LOD score at which a group (comprising 10 or more markers) was separated. In these cases, the larger of the two resulting groups was retained. The resulting 25 linkage groups (independence LOD scores 10.0 ≤ n ≤ 24.0) were subjected to additional mapping. Groupings of markers eliminated during this or a subsequent round of mapping were excluded from further analysis.

The second round of mapping produced a 2556.6-cM linkage map composed of 25 linkage groups, each consisting of 25 to 171 markers, ranging in length from 31.18 to 142.78 cM, with mean intermarker distances ranging from 0.47 to 3.66 cM. Using the same criteria described above, an additional group (comprising 25 markers and an average intermarker distance of 3.658) was eliminated. A densely populated group with a high independence LOD (153 markers; 135.73 cM; independence LOD of 24.0) was split and 12 linkage groups (103.982 ≤ n ≤ 142.783 cM) were trimmed.

The result of this third and final round of mapping was then analyzed for genomic synteny shared between *Astyanax mexicanus* and the zebrafish genome and used to map albinism as a proof of concept. Albinism was scored as a binary phenotype wherein presence of melanin (0) or absence of melanin (1) was assigned to each of the members of our experimental F_2_ pedigrees. All QTL analyses of albinism were conducted using R/qtl (Broman *et al.* 2003) run for each of three scan-one mapping methods: marker regression (MR), expect maximum (EM), and Haley-Knott (HK), according to the methodology in Gross *et al.* (2014).

### Assignment of genomic synteny between the *Astyanax mexicanus* and *Danio rerio* genomes

At present, physical genome resources for *Astyanax mexicanus* are in their early draft phases (McGaugh *et al.* 2014). Therefore, we anchored our GBS-based linkage map to the physical genome of the most closely related fish model system with comprehensive resources, *Danio rerio*. *Astyanax* and *Danio* are members of the superorder Ostariophysii, which diverged ∼150 My ago (Briggs 2005). Despite this distance, significant genome-level synteny remains between these species (Gross *et al.* 2008; O’Quin *et al.* 2013). Our GBS marker set was derived from endonuclease restriction site-based libraries and was therefore anonymous. We first identified all GBS markers that could be directly localized to a conserved region in the *D. rerio* genome. Accordingly, we performed BLAST searches of the 64-bp sequences comprising our marker sequences directly against the *Danio* genome (downloaded from the Ensembl genome browser; www.ensembl.org).

These and all subsequent searches were performed using a BLASTN script run on the Ohio Supercomputing Cluster (OSC). All quality control defaults, including an expect value (e-value) cutoff of 10, were maintained. The script permitted the return of alignments between a given 64-bp marker sequence and regions of up to three distinct targets (*e.g.*, three different *Danio rerio* chromosomes). In cases where a single marker sequence aligned multiple times with the same target, raw results were filtered by e-value, retaining the lowest e-value alignment for each marker-target pairing. There are two 64-bp sequences for each GBS marker, differing only in that each contains one of the two alleles for the imbedded SNP. Because both of these sequences were included when BLAST searches using the 64-bp marker sequences were conducted, this filtering step also served to collapse these results into a single set of results, retaining the better of the two alignments for each marker-target pairing.

In some instances, a single queried sequence returned alignments with multiple targets. These instances were resolved by sorting results to determine the “top hit,” which was defined as having the lowest e-value and highest percent identity (in case of an e-value tie) to a particular target sequence. If the target of the top hit (*i.e.*, the alignment with the lowest e-value) for a given marker sequence agreed with the target reported for one or more other markers on the same linkage group that returned only a single robust hit, then the top hit for the marker in question was considered “supported” and retained. If the top hit was not supported in this fashion but a different BLAST result was, then the latter “not top hit, supported” result was retained instead. If none of the results returned for a marker sequence were supported, then the top hit was retained, despite the lack of support. In rare cases, there was no way to resolve which result should be retained. Results for these “unresolved” markers were discarded.

When using BLAST searches to align our 64-bp markers directly to the *Danio rerio* genome returned relatively few high-quality hits, we developed a strategy whereby we first aligned our GBS marker sequences to the *Astyanax mexicanus* genome and transcriptome data. This information was then used to identify homologous *Danio* genomic and transcriptomic sequences. Current genomic resources in *Astyanax* consist of >10,000 unplaced genomic scaffolds (Bioproject PRJNA89115). The collective sequence data for the *Astyanax* genome (GenBank Assembly ID GCA_000372685) were downloaded from Ensembl, along with the transcript sequences for 23,042 predicted genes. BLAST searches were used to determine putative locations for the 64-bp sequences of the 2235 GBS markers comprising our final linkage map in the *Astyanax* genomic and transcriptomic data sets. After initial searches were performed as described, ∼2000-bp stretches of genomic sequence harboring our 64-bp GBS marker sequences were aligned with the *Danio* genome. Similarly, full sequences for predicted *Astyanax* transcripts to which our GBS markers aligned were queried against a *Danio* cDNA database downloaded from Ensembl. Both data sets were then filtered (as described), yielding a single “best” *Danio* alignment for each informative query. This process enabled us to leverage draft genomic and transcriptomic data to augment the amount of sequence information associated with our 64-bp GBS markers and to identify homologous genomic positions in a well-characterized model system.

After BLAST searches using the direct, genomic, and transcriptomic alignment methods were completed, the filtered results for all three were combined. When multiple methods returned results for the same marker, a single result was chosen and retained using the same filtering process applied to single data sets (above). The Circos program (Krzywinski *et al.* 2009) was used to visualize comparative genomic positions between our linkage map and the *Astyanax* and *Danio rerio* genomes.

### Position identification for previously published markers in the Astyanax genome

Previous maps published by Gross *et al.* (2008) and O’Quin *et al.* (2013) were used to examine synteny between *Astyanax* and *Danio* and to provide a comparison between this study and prior studies. These authors provided predicted *Danio* positions for the markers used in their analyses, but positions in the draft *Astyanax* genome were not determined because these studies predated available genomic resources. Our GBS-based map does not share any markers with the two previous maps, so it was necessary to identify positions of previously generated markers in *Astyanax* to enable comparison between previous mapping efforts and those described here. Accordingly, microsatellite and RAD-seq marker sequences (where available) for each data set were aligned with *Astyanax* genome scaffolds using the same BLAST and filtering protocols used for our own data (above). Both previous studies included markers located in candidate genes. The locations of *Astyanax* orthologs of these candidate genes were identified using Ensembl.

GBS marker sequences and genotyping data are available from the Dryad Digital Repository (http://dx.doi.org/10.5061/dryad.6s718).

## Results and Discussion

### A high-density linkage map in *Astyanax mexicanus*

**Figure 1:**
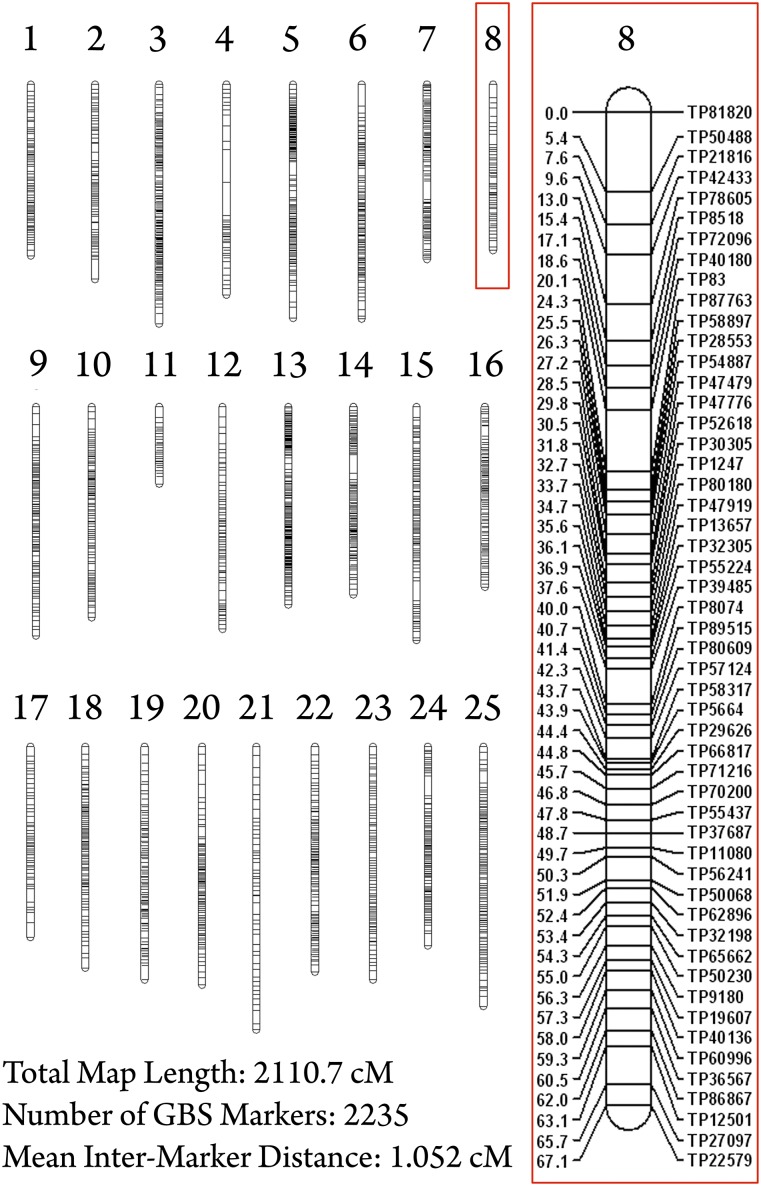
A GBS-based linkage map in the Mexican cave tetra, *Astyanax mexicanus*. We analyzed 3003 SNP markers in 170 individuals using genotyping-by-sequencing technology. This linkage map consists of 2235 markers in 25 linkage groups (*A. mexicanus* karyotype number = 25), spanning a total distance of 2110.7 cM (mean intermarker distance = 1.052 cM). *Astyanax* linkage group 8 (red box) illustrates typical marker density observed in most groups. This group consists of 52 GBS markers spanning 67.061 cM with a mean intermarker distance of 1.315 cM.

Here, we present a dense linkage map for *Astyanax mexicanus* generated using genotyping-by-sequencing technology. This map was created using 170 experimental F_2_ individuals based on genotypic information for 3003 loci. The construction of this map ultimately yielded 25 linkage groups (the karyotypic number for *Astyanax*) comprising 2235 markers spanning 2110.7 cM, with an average intermarker distance of 1.052 cM (Figure 1, Supporting Information, Table S1). The strategy we used enables application of powerful, cost-effective, next-generation sequencing technology to facilitate genetic studies in emerging or nonmodel systems.

**Figure 2:**
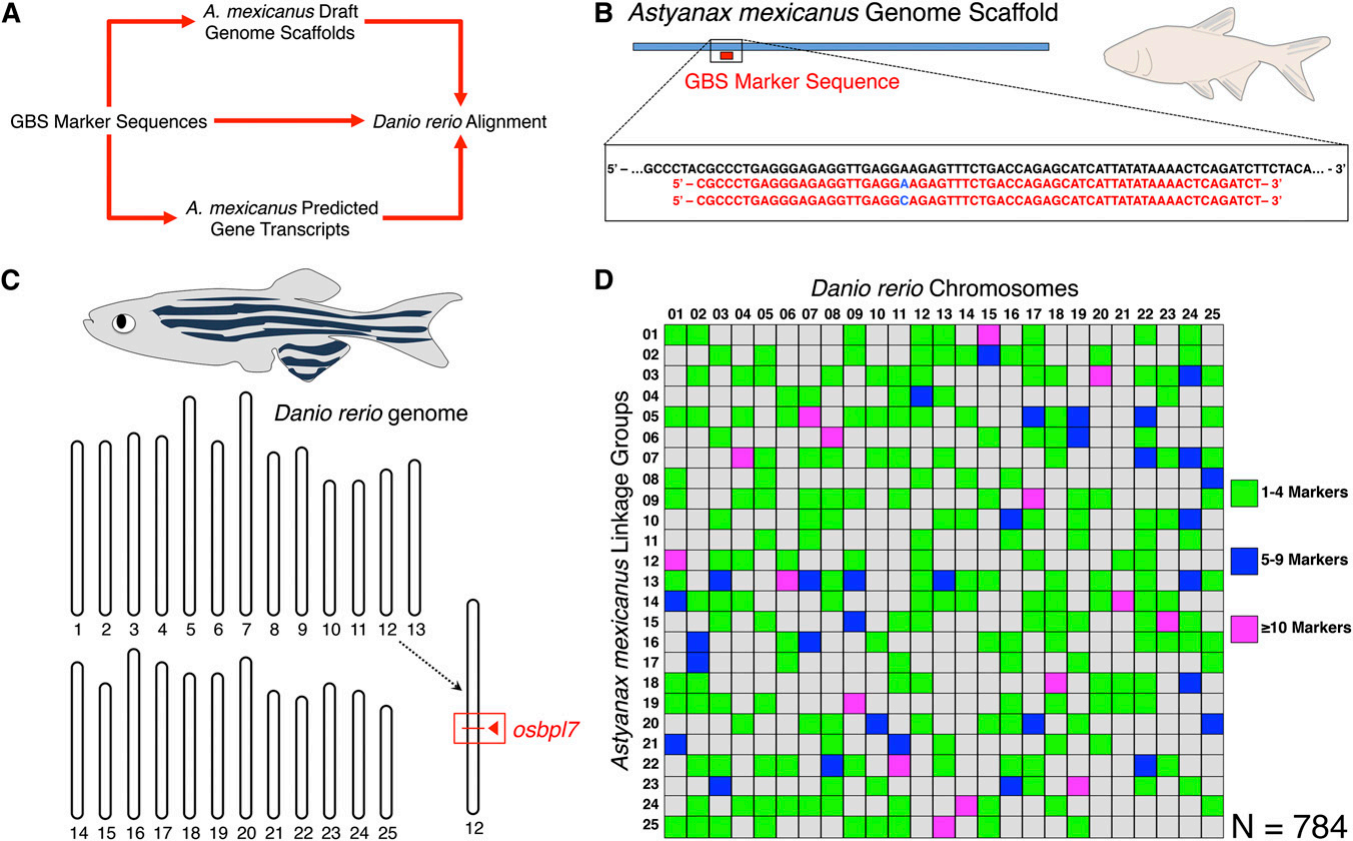
Short GBS sequences identify syntenic stretches between two Ostariophysian freshwater fish species. To reveal syntenic regions between *Astyanax mexicanus* and *Danio rerio*, we first identified stretches of the *Danio* genome harboring homologous sequences to our anonymous GBS marker sequences (A). Individual 64-bp sequences for the 2235 GBS markers in our linkage map were compared with the *Danio* genome both directly and by first aligning to larger *Astyanax* genomic scaffolds and predicted gene transcripts (B), followed by alignment of some or all of the larger sequence to the *Danio* genome based on BLAST sequence analysis (C). This resulted in identification of homologous sequences for 784 *Astyanax* GBS markers within the *Danio* genome. The markers shared between *Danio* chromosomes and *Astyanax* linkage groups are represented using an Oxford plot (D).

**Figure 3:**
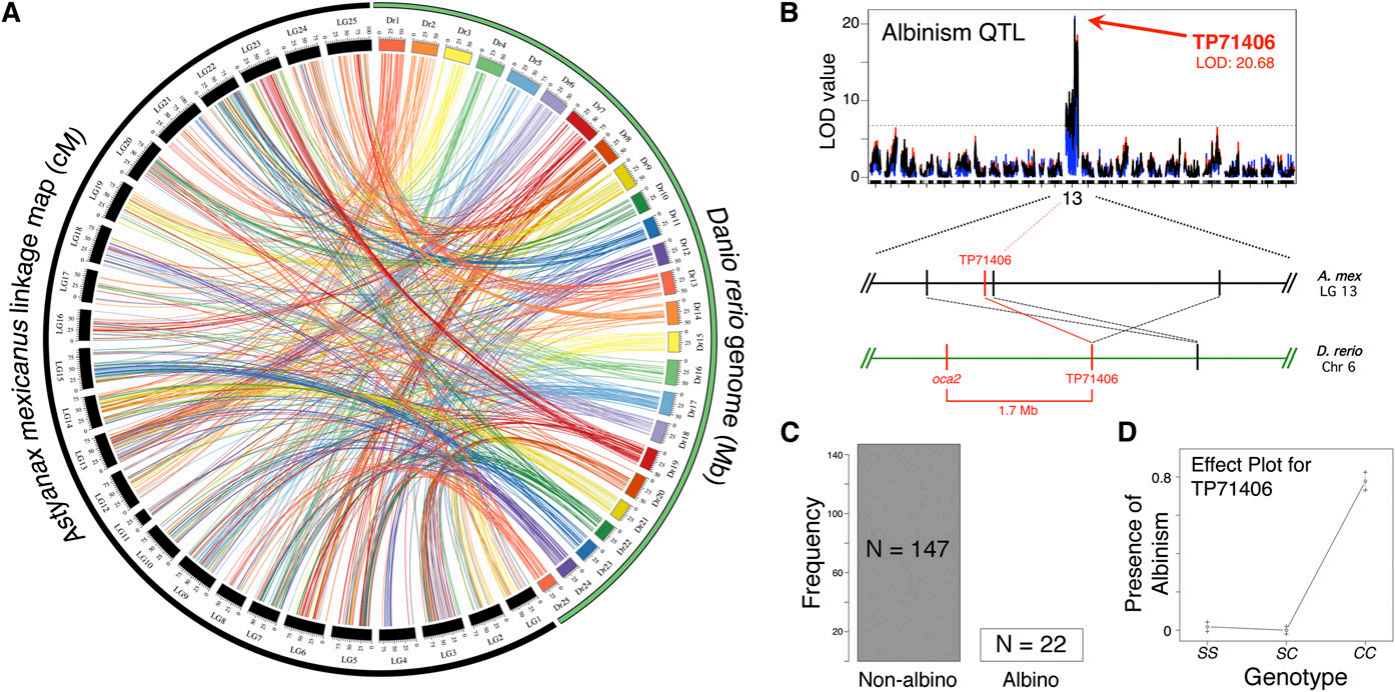
Whole-genome synteny between *Astyanax* and *Danio* and a proof-of-concept analysis of albinism. Syntenic links between our GBS map and the *Danio* genome were visualized using Circos (A). Each line represents a connection between the position of a particular marker in our linkage map (black; scale in cM) and a homologous sequence in *Danio* (various colors; scale in Mb). We scored albinism, a Mendelian trait associated with the *Oca2* gene in cave-dwelling *Astyanax* (C), and performed QTL analysis using R/qtl. Each of three mapping methods (MR in red; EM in blue; HK in black) revealed peak LOD scores of ∼20 (LOD at 0.001α threshold = 6.75) at, or adjacent to, GBS marker TP71406 on *Astyanax* linkage group 13 (B). Homologous sequences to TP71406 and several of its neighbors on *Astyanax* linkage group 13 are clustered together on *Danio* chromosome 6 near the *Oca2* gene. A phenotypic effect plot for marker TP71406 revealed the predicted association between the homozygous “cave” condition (genotype *CC*) and albinism in F_2_ individuals (D).

Cross-genera marker identification was greatly facilitated by alignment first to draft *Astyanax* genomic and transcriptomic resources, followed by searches of the homologous sequences in *Danio* (Figure 2, A–C). Although direct BLAST searches of our 64-bp GBS marker sequences returned results for few of the markers in our map (1.2%), success rates were much higher when using *Astyanax* genomic (26.5%) or transcriptomic (13.3%) sequences as an intermediary (Table 1). Each *Danio rerio* chromosome was represented in our comparative genomic analysis, with *Astyanax* linkage groups containing 14–52 markers (average = 30.84) comprising ancient syntenic blocks shared with each of 25 zebrafish chromosomes (Figure 2D). Of the 2235 GBS markers that constitute our linkage map, 784 marker sequences (35.1%) were successfully identified in the *Danio rerio* genome (Figure 3A).

**Table 1:** Summary of BLAST results and identification of markers used in *Astyanax*-to-*Danio* syntenic analysis

	*GBS Markers* to *Danio* Genome*^a^*	GBS Markers to *Astyanax* Genome*^b^*	*Astyanax* Genome to *Danio* Genome*^c^*	GBS Markers to *Astyanax* Transcriptome*^d^*	*Astyanax* Transcriptome to *Danio* Transcriptome*^e^*
**Total no. of BLAST queries**	2235	2235	2088	2235	572
**BLAST result categories**					
Single robust hit	14 (0.6%)	1838 (82.2%)	255 (12.2%)	508 (22.7%)	110 (19.2%)
Top hit, with positional support	0 (0.0%)	173 (7.7%)	92 (4.4%)	15 (0.7%)	120 (21.0%)
Top hit, without positional support	10 (0.4%)	71 (3.2%)	138 (6.6%)	60 (2.7%)	61 (10.7%)
Not top hit, with positional support	2 (<0.1%)	6 (0.3%)	108 (5.2%)	2 (<0.1%)	7 (1.2%)
Unresolved	4 (0.2%)	14 (0.6%)	4 (0.2%)	12 (0.5%)	0 (0.0%)
No result	2205 (98.7%)	133 (6.0%)	1491 (71.4%)	1638 (73.3%)	274 (47.9%)
**Identified syntenic markers between *Astyanax* and *Danio***	26	N/A	593	N/A	298

a Results of 64-bp GBS markers BLASTed directly to the *Danio rerio* genome.

b Results of 64-bp GBS markers BLASTed directly to the *Astyanax* genome draft assembly.

c Results of ∼2-kb genomic intervals harboring 64-bp GBS markers BLASTed to the *Danio rerio* genome.

d Results of 64-bp GBS markers BLASTed directly to the *Astyanax* predicted transcriptome.

e Results of *Astyanax* transcripts harboring 64-bp GBS markers BLASTed to the *Danio rerio* transcriptome.

We performed a proof-of-concept analysis using the albinism phenotype to validate the utility of our GBS-based linkage map (Figure 3, B–D). Accordingly, we mapped the monogenic trait of albinism using the R/qtl package to evaluate phenotypic and genotypic data for the 170 F_2_ hybrid individuals used to construct our map. We identified a peak LOD score of 20.68 on linkage group 13, associated with marker TP71406. This marker and the surrounding region form a syntenic block within a region of *Danio rerio* chromosome 6. This genomic interval contains the gene *Oca2*, previously demonstrated to be the causative locus for albinism in *Astyanax* cavefish. This supports previous findings of conserved synteny inclusive of significant portions of chromosome 6 in *Danio* (Gross *et al.* 2008; O’Quin *et al.* 2013) and implies our densely populated map will enable future QTL studies of trait evolution in *Astyanax*.

### Conserved genomic architecture between *Astyanax* and *Danio* based on GBS markers

Our analysis of synteny between *Astyanax* and *Danio* illustrates variable levels of genomic conservation across linkage groups (Figure 2D, Figure 3A). Certain chromosomes, for instance, appear to have changed little since the divergence of these teleost species (*e.g.*, *Danio* chromosomes 6 and 23 in *Astyanax* linkage groups 13 and 15, respectively). However, other *Danio* chromosomes appear scattered across several linkage groups, without a consensus representation for any particular group (*e.g.*, *Danio* chromosomes 2 and 5).

We believe these findings most likely reflect genomic rearrangements that have occurred since the divergence of these two species. However, this finding could also be attributed to low representation of particular *Danio* chromosomes within our GBS marker set. We examined this possibility by assessing the number of syntenic links between our GBS-based linkage map and each *Danio* chromosome. We would anticipate that longer chromosomes would naturally harbor more syntenic links. Values were therefore expressed as a ratio of syntenic links per megabase (mean = 0.59 GBS markers/Mb). Although the mean value for chromosomes that were not strongly represented on any particular linkage group in our map (*i.e.*, had fewer than 10 syntenic links with each linkage group, mean = 0.52 GBS markers/Mb, n = 8) was lower than that for chromosomes demonstrating strong synteny with a particular linkage group (mean = 0.61 GBS markers/Mb, n = 17), there was not a significant difference between the two groups (t_23_ = 0.5809, *P* = 0.5670). This leads us to conclude that, although representation of particular chromosomes in our data set may be a contributing factor, it is unlikely that this is the primary cause of the differences in chromosomal representation patterns observed.

Alternatively, BLAST results for *Astyanax* GBS markers (or the larger *Astyanax* sequences to which they were aligned) may include paralogous genes or otherwise ambiguous results that could lead to erroneous links between a linkage group and a *Danio* chromosome. Although we cannot rule out this possibility, we feel our strategy prioritized the “optimal” BLAST result among multiple hits for a single marker leading to alignments that agree with nearby unambiguous results (Table 1). As a result, of the 784 markers in our map for which a putative *Danio* position was determined, only 15.9% (n = 125) of final calls were unsupported by the results for other markers belonging to the same linkage group (Table S1). Given that chromosomal arrangements have occurred over the ∼150 My since divergence, we feel our systematic approach best identifies paralogous genes and other potential sources of ambiguity.

Erroneous or ambiguous genotyping data may have led to incorrect assignment of “cave” and “surface” alleles for particular markers. These erroneous assignments could have adversely affected downstream efforts, causing markers to be incorrectly placed during the grouping and/or mapping stages of linkage map construction. All efforts were made to ensure allelic identification was accurate using a stringent screening process (see *Materials and Methods*); however, we relied on a relatively small number of cave, surface, and F_1_ hybrid individuals (n = 4 each) to identify parental allelic origin. Similarly, the relatively small number of meiotic events represented by the 170 F_2_ individuals may have resulted in linkage map inaccuracies (Gross *et al.* 2008). Future comparisons between the map we present here and a finished-grade *Astyanax* genome will clarify if regions lacking synteny between *Astyanax* and *Danio* are attributable to errors in our linkage map or genomic rearrangements that have occurred since the divergence of these taxa.

### Unplaced *Astyanax* genome scaffolds can be anchored to our new linkage map

Positional locations in the current draft of the *Astyanax* genome were established for 93.6% (n = 2091) of the 2235 GBS markers present in our map. These markers were localized to positions spread across 598 different *Astyanax* genome scaffolds. Our 25 *Astyanax* linkage groups contain markers representing between 12 (linkage groups 8 and 22) and 55 (linkage group 3) genome scaffolds each, with a map-wide average of 27.64 scaffolds/linkage group. Individual genome scaffolds contained between 1 and 31 GBS markers appearing in our final map, with an average of 3.50 markers per scaffold. GBS markers located on the same genomic scaffold colocalized to a single linkage group 87.3% of the time. This suggests that our recombination mapping successfully recapitulated the true genomic positions of the markers used to construct our map.

### Improved linkage mapping resources in *Astyanax*

We sought to compare our linkage map with maps previously published by Gross *et al.* (2008) and O’Quin *et al.* (2013) that also examined synteny between *Astyanax* and *Danio*. Metrics such as the number of linkage groups, total map length, number of markers, and marker density are commonly used to compare linkage maps within species. Both our GBS-based map and the RAD-seq and microsatellite-based map published by O’Quin *et al.* (2013) consist of 25 linkage groups, matching the *Astyanax mexicanus* karyotype number of 25. The microsatellite-based map presented by Gross *et al.* (2008) contains 28 groups (Table 2). Although our map is of comparable length, it represents a dramatic increase in marker number (+559% compared with that of Gross *et al.* 2008; +320% compared with that of O’Quin *et al.* 2013) and marker density (+473% compared with that of Gross *et al.* 2008; +279% compared with that of O’Quin *et al.* 2013) relative to previously published linkage maps for this system. As a result, we saw a substantial increase in the number of syntenic links between our map and *Danio* (+506% compared with that of Gross *et al.* 2008; +453% compared with that of O’Quin *et al.* 2013) and an increase in the number of unplaced *Astyanax* scaffolds that can be anchored to our map (+263% compared with that of Gross *et al.* 2008; +171% compared with that of O’Quin *et al.* 2013).

**Table 2:** Comparison of *Astyanax* linkage maps and syntenic studies with *Danio rerio*

	**Gross *et al.* 2008**	**O’Quin *et al.* 2013**	**Current Analysis**
Total no. of linkage groups	28	25	25
Total no. of genomic markers	400	698	2235
Linkage map length	1783 cM	1835.5 cM	2110.7 cM
Marker density	0.224 per cM	0.380 per cM	1.06 per cM
Marker type	Microsatellite	Microsatellite + RAD-seq	Genotyping-by-sequencing
No. of *Astyanax* genomic scaffolds represented by map	227	350	598
No. of syntenic markers identified between *Astyanax* and *Danio*	155	173	784

Our map contains a total of 784 links between our linkage groups and the *Danio rerio* genome and an average of 30.84 links (minimum = 14, maximum = 52) per *Danio rerio* chromosome (Table 3). This represents a considerable improvement over the results presented by Gross *et al.* (2008) (155 total links, average links per *Danio* chromosome = 6.20, minimum = 0, maximum = 15) and O’Quin *et al.* (2013) (173 total links, average links per *Danio* chromosome = 6.92, minimum = 1, maximum = 20). Additionally, although instances of synteny strongly represented in previous maps were also identified in this analysis, our map demonstrated increased representation of certain *Danio* chromosomes poorly represented in previous maps. For example, Gross *et al.* (2008) did not identify links between their map and *Danio rerio* chromosome 11; however, we identified 36 links between our map and chromosome 11. Similarly, *Danio* chromosomes 17 and 19 are each represented once in the map of O’Quin *et al.* (2013). We identified substantial links between these chromosomes and our linkage groups 9 (n = 21) and 23 (n = 15), respectively.

**Table 3:** Comparison of syntenic analyses between *Astyanax* linkage maps and their association with the *Danio rerio* genome across multiple studies

	**Gross *et al.* 2008**			**O’Quin *et al.* 2013**			**Current Analysis**		
*Danio rerio* Chromosome	No. of Syntenic Links*^a^*	Represented Linkage Group(s)*^b^*	No. of Represented *Astyanax* Genome Scaffolds*^c^*	No. of Syntenic Links	Represented Linkage Group(s)	No. of Represented *Astyanax* Genome Scaffolds	No. of Syntenic Links	Represented Linkage Group(s)	No. of Represented *Astyanax* Genome Scaffolds
1	13	5, **8**, 21	7	15	**4**, 5, 9, 18, 21, 23	11	42	1, 5, 8, 9, **12**, 13, **14**, 18, 19, **21**, 25	26
2	6	2, 14, 15, 22	2	6	7, 12, 13, 16, 23	5	23	1, 3, 5, 14, **16**, **17**, 18, 19, 22, 24, 25	20
3	6	1, 4, 19	6	4	4, 15, 25	3	28	2, 6, 10, 12, **13**, 14, 15, 19, 22, **23**, 25	23
4	3	6, 7	2	4	3	4	24	3, 5, **7**, 9, 12, 14, 20, 24	17
5	15	**1**, 5, 9, 10, 20	9	13	**2**, 8, 16, 17, 19	11	22	2, 3, 7, 8, 9, 11, 15, 19, 22, 24, 25	17
6	9	**4**, 13	4	20	1, 2, 11, 16, 18	16	37	4, 6, 12, **13**, 16, 17, 22, 24	23
7	11	17, 22, 24, 26	10	6	13, 22, 23, 25	6	31	4, **5**, 7, 9, 10, 11, **13**, **16**, 20, 24	25
8	4	9, 12	4	7	7, 14, **17**	6	42	3, **6**, 7, 9, 10, 13, 14, 20, 21, **22**, 23, 24	29
9	5	3, 17	4	8	10, **11**	7	31	1, 2, 5, 9, 12, **13**, **15**, **19**, 22, 25	23
10	3	17, 18	3	4	8, 10, 14	4	14	3, 5, 7, 16, **20**, 23, 25	12
11	0	—	0	5	14, 17, 22	5	36	3, 4, 5, 7, 9, 15, 17, 18, **21**, **22**, 24, 25	24
12	7	**10**, 16	4	6	**24**	4	26	1, 2, 3, **4**, 5, 8, 11, 12, 13, 14, 15, 18, 20	18
13	11	1, **5**	6	7	**4**, 12	4	34	1, 2, 4, 7, 10, **13**, 14, 21, 22, **25**	21
14	6	6, 7	4	9	3, **6**, 15, 19	6	24	2, 5, 8, 10, 13, 14, **24**	16
15	5	**2**	5	8	1, 7, **12**, 14	6	27	**1**, **2**, 6, 9, 13, 16, 20, 24, 25	17
16	3	13	3	7	**8**, 19	7	23	2, 8, **10**, 11, 16, 17, 19, 20, 22, **23**	17
17	6	3, **23**	2	1	20	1	52	1, 2, 3, **5**, 6, **9**, 10, 12, 14, 15, **20**, 22, 23	34
18	8	**11**	3	7	**5**	3	32	3, 5, 6, 7, 13, 14, 15, 16, **18**, 19, 21, 24	26
19	3	19	3	1	25	1	42	**5**, **6**, 9, 10, 11, 15, 17, 20, **23**, 25	28
20	7	**1**, 2	7	3	1, 2	3	21	2, **3**, 9, 13, 14, 18, 19, 21	15
21	3	15, 17	1	6	2, **7**	6	16	12, **14**, 18, 19	12
22	4	12, 20	4	7	14, 18, 22	6	42	1, 3, **5**, 6, **7**, 9, 10, 11, 12, 13, 14, 15, 16, 18, 19, **22**, 23	32
23	6	**26**	3	7	14, 16, 18, 25	6	33	3, 4, 7, 10, 14, **15**, 16, 22	17
24	8	1, 13, 15	6	9	2, 3, 8, 11	8	38	1, 2, **3**, **7**, **10**, 11, **13**, 15, 16, **18**, 23	25
25	3	6, 7	3	3	3, 6	3	31	3, 5, 7, **8**, 9, 13, 16, 17, **20**, 24	19

**Bold** indicates that a listed linkage group harbors five or more links with a given *Danio* chromosome.

a Indicates the number of syntenic links identified between *Astyanax* linkage maps and each listed *Danio rerio* chromosome.

b Indicates the identity of *Astyanax* linkage groups harboring syntenic links with each listed *Danio rerio* chromosome.

c Indicates the number of *Astyanax* genome scaffolds harboring connections with each listed *Danio rerio* chromosome.

Our linkage map uses an entirely different marker set than those used in previous maps. Therefore, it was not possible to make direct comparisons with the linkage groups across prior studies. However, we could indirectly compare maps by examining connections between *Astyanax* genomic scaffolds and each linkage map. We examined the five strongest syntenic links between single linkage groups in our GBS-based map and single *Danio* chromosomes and then identified analogous connections between those chromosomes and specific linkage groups in the maps presented by Gross *et al.* (2008) and O’Quin *et al.* (2013).

*Astyanax* genomic scaffolds harboring markers associating each linkage group with a particular *Danio* chromosome were then compared (Table 4). We found that many of the identified *Astyanax* genomic scaffolds colocalize to putatively analogous linkage groups in both our GBS-based map and those of Gross *et al.* (2008) and/or O’Quin *et al.* (2013). However, in every case examined, our linkage groups were inclusive of a much higher number of *Astyanax* genomic scaffolds compared with prior studies. Thus, while the linkage groups in our map represent genomic intervals similar to those represented in prior maps, our map achieves a higher level of detail and resolution. These results also suggest that future mapping efforts in *Astyanax* may benefit by combining GBS marker discovery with those markers used by Gross *et al.* (2008) and O’Quin *et al.* (2013) to generate the most comprehensive linkage mapping resource.

**Table 4:** Representative analysis of linkage group equivalence and quality based on highly syntenic chromosomes in *Danio rerio* and linkage groups in *Astyanax mexicanus*

	**Gross *et al.* 2008**			**O’Quin *et al.* 2013**			**Current Analysis**		
*Danio rerio* Chromosome	Principal Represented Linkage Group*^a^*	No. of Syntenic Links*^b^*	Identity of Represented *Astyanax* Genome Scaffolds*^c^*	Principal Represented Linkage Group	No. of Syntenic Links	Identity of Represented *Astyanax* Genome Scaffolds	Principal Represented Linkage Group	No. of Syntenic Links	Identity of Represented *Astyanax* Genome Scaffolds
6	4	8	KB871811.1, **KB882115.1**, **KB882122.1**, **KB882161.1**, *KB882172.1*, *KB882176.1*	1	16	**KB871670.1**, KB871811.1, *KB871878.1*, *KB872044.1*, KB872200.1, **KB882115.1**, **KB882120.1**, **KB882122.1**, **KB882161.1**, *KB882172.1*, *KB882176.1*, **KB882185.1**	13	28	*KB882256.1*, *KB882253.1*, *KB882235.1*, KB882230.1, **KB882185.1**, *KB882171.1*, **KB882161.1**, *KB882152.1*, **KB882122.1**, **KB882120.1**, **KB882115.1**, *KB882082.1*, KB872595.1, **KB871670.1**
8	9	3	KB871816.1, KB871923.1, **KB882105.1**	17	5	**KB871601.1**, *KB871607.1*, **KB871684.1**, KB871923.1, KB872214.1	6	17	*KB882289.1*, *KB882113.1*, **KB882105.1**, KB872252.1, *KB871939.1*, KB871817.1, **KB871684.1**, **KB871601.1**, *KB871595.1*
13	5	9	*KB871819.1*, **KB872081.1**, **KB872296.1**, **KB882107.1**, **KB882118.1**, *KB882125.1*	4	6	KB881455.1, **KB872296.1**, **KB882107.1**, **KB872081.1**	25	17	*KB882261.1*, *KB882210.1*, *KB882154.1*, **KB882118.1**, KB882109.1, **KB882107.1**, **KB872296.1**, **KB872081.1**, *KB871838.1*, KB871652.1, *KB871591.1*
17	23	5	**KB882084.1, KB882233.1, KB882265.1**	20	1	**KB882265.1**	9	21	**KB882265.1,** *KB882243.1*, **KB882233.1,** *KB882179.1*, KB882158.1, *KB882153.1*, KB882117.1, **KB882084.1,** KB872047.1, KB871726.1, KB871695.1
23	26	6	KB872166.1, KB880082.1, **KB882102.1**, KB882242.1	18	4	*KB882098.1*, **KB882102.1**, *KB882128.1*	15	20	KB882214.1, *KB882138.1*, *KB882128.1*, **KB882102.1**, *KB882098.1*, KB872132.1, KB872075.1, KB871985.1

**Bold** indicates genomic scaffolds containing syntenic markers on the principal represented linkage group in our GBS-based map and one or more previous maps. *Italic* lettering indicates scaffolds that contain a syntenic marker in the GBS-based map and are associated with the principal linkage group(s) in previous map(s) but do not contain a syntenic marker (and *vice versa*).

a Indicates the most common (*i.e.*, “principal”) linkage group anchoring to the indicated *Danio rerio* chromosome.

b Indicates the number of points of synteny between the principal linkage group from this article and the indicated *Danio rerio* chromosome.

c Lists the identity of *Astyanax* genomic scaffolds to which each point of synteny identifies.

### High-density GBS-based linkage mapping will inform the Astyanax genome sequencing project

Preliminary *Astyanax* genomic resources enabled us to locate 64-bp, anonymous GBS markers, and to assess the quality and reliability of our *Astyanax* linkage map. This emerging resource did not allow us to determine how well the 25 *Astyanax* chromosomes are represented in our map. However, these resources allowed us to determine if markers predicted to occur in the same genome scaffolds also co-occur in our GBS-based linkage map. Overall, we observed a high level of agreement between our linkage groups and one or more unplaced *Astyanax* genomic scaffolds.

**Figure 4:**
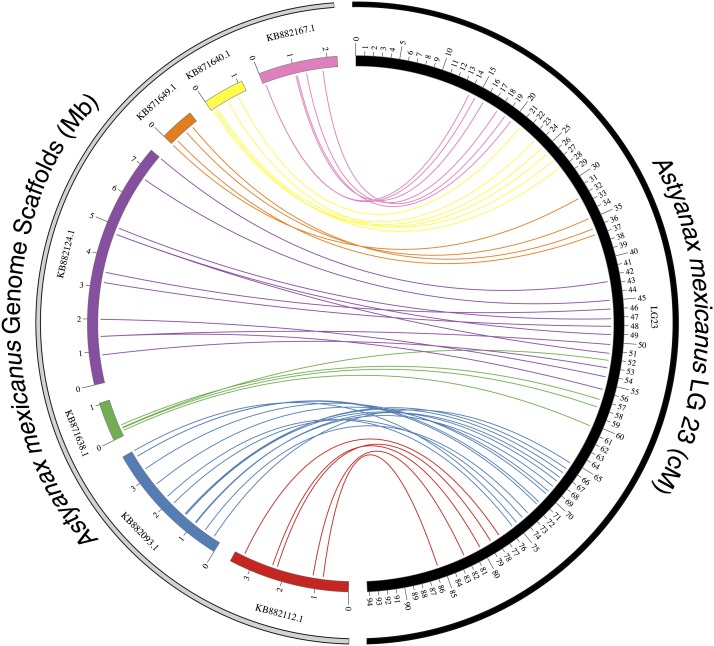
Colinearity between *Astyanax* linkage groups and genome scaffolds. We visualized the “anchoring” of seven unplaced *Astyanax* genome scaffolds (various colors) to linkage group 23 (black) in our *Astyanax* linkage map. For clarity, only scaffolds harboring four or more GBS markers were included. Scaffolds correspond to discrete, colinear sections of the linkage group with minimal overlap. The linear arrangement of markers is largely preserved between the scaffold and the linkage group. The scale for *Astyanax* scaffolds is in Mb; the scale for linkage group 23 is shown in cM.

In many cases, markers present on the same scaffold clustered together over a portion of a linkage group with little or no interruption from unplaced markers or markers from other scaffolds (Figure 4). We expect these results will help inform chromosomal positions of scaffolds, given that linkage maps have been successfully used to augment genomic resources in other fish species, including several species of catfish (Liu 2011; Ninwichian *et al.* 2012), rainbow trout (Palti *et al.* 2011; Palti *et al.* 2012), and Atlantic salmon (Lorenz *et al.* 2010). We believe our high-density GBS-based map resources will both provide a resource for more refined QTL analyses and inform the genomic architecture of the *Astyanax* genome sequencing project.

## Conclusions

We constructed a high-density linkage map for *Astyanax mexicanus* based on high-throughput genotyping-by-sequencing data. We leveraged emerging *Astyanax* genomic and transcriptomic resources and *Danio rerio* genomic and transcriptomic data to locate syntenic regions shared between our map and the *Danio* genome. These findings were based on the physical position of homologous (64-bp) GBS marker sequences. As expected, based on the significant divergence between these species, we recovered varying levels of synteny between portions of our *Astyanax* linkage groups and regions of the *Danio* genome. As a proof of concept, we successfully mapped a strong QTL associated with albinism and demonstrated significant conserved genomic architecture in the regions surrounding the gene *Oca2*, between *Astyanax* and *Danio*. We successfully anchored emerging *Astyanax* genomic information to our GBS-based linkage map, identifying the putative location of thousands of anonymous GBS marker sequences within unplaced *Astyanax* genome scaffolds. This strategy revealed significant colinearity between genomic scaffolds and our linkage map, and it demonstrated the utility of high-density, GBS-based linkage maps to inform and improve nascent genomic resources. Multiple comparisons with previously published maps suggest that our GBS-based map offers a higher level of resolution and a greater number of connections between *Astyanax* and *Danio* genomes. We hope that this resource and technology will accelerate the search and identification of genes mediating cave-associated traits in *Astyanax*, facilitate the genomic assembly for this system, and prove useful to other natural model systems of evolutionary and biomedical relevance.

## Supplementary Material

Supporting Information
