# Supplementary material for: A High-Density Linkage Map for Astyanax mexicanus Using Genotyping-by-Sequencing Technology
Source: G3 (Bethesda). 2014 Dec 17;5(2):241–51. doi: 10.1534/g3.114.015438 (PMC4321032; doi:10.1534/g3.114.015438)
Supplement: Supporting Information [file supp_5_2_241__index.html]

A High-Density Linkage Map for Astyanax mexicanus Using Genotyping-by-Sequencing Technology — Supporting Information 

# A High-Density Linkage Map for *Astyanax mexicanus* Using Genotyping-by-Sequencing Technology

## Supporting Information for Carlson, Onusko, and Gross, 2015

**Files in this Data Supplement:**

- Table S1 - GBS marker positions in a high-density *Astyanax* linkage map. (PDF, 309 KB)
