# Supplementary material for: A High-Density Linkage Map for Astyanax mexicanus Using Genotyping-by-Sequencing Technology
Source: G3 (Bethesda). 2014 Dec 17;5(2):241–51. doi: 10.1534/g3.114.015438 (PMC4321032; doi:10.1534/g3.114.015438)
Supplement: Supporting Information [file supp_g3.114.015438_015438SI.pdf]

G3 · Genes | Genomes | Genetics

A high-density linkage map for *Astyanax mexicanus* using genotyping-by-sequencing technology

Brian M. Carlson, Samuel W. Onusko and Joshua B. Gross[1]

Department of Biological Sciences, University of Cincinnati, Cincinnati, Ohio 45221

[1]*Corresponding author*: Joshua B. Gross, 312 Clifton Court, 711B Rieveschl Hall, Cincinnati, Ohio 45221 Email: grossja@ucmail.uc.edu

DOI: 10.1534/g3.114.015438

Table S1. GBS marker positions in a high-density *Astyanax* linkage map.

GBS Marker	Linkage Group	cM Position	Position in *Astyanax* Draft Genome[a]	Syntenic Position in *Danio* Genome[b]
TP54223	1	0	KB882088.1:4198831-4198894	22:24745776-24745709
TP9691	1	2.731	KB882088.1:4515859-4515796	-
TP81727	1	4.631	-	-
TP59453	1	5.796	KB882088.1:3742373-3742433	-
TP15732	1	7.572	KB882088.1:3919659-3919722	-
TP44161	1	8.923	KB882088.1:3919746-3919683	-
TP45220	1	10.206	KB882088.1:3815399-3815336	12:15418236-15418053
TP88528	1	11.09	KB882088.1:3482528-3482465	-
TP31042	1	13.44	KB882088.1:3103712-3103649	-
TP44805	1	14.465	KB882088.1:1909653-1928337	2:22309476-22318822
TP24626	1	15.041	KB873588.1:901-838	-
TP1091	1	17.691	KB882088.1:3635891-3635828	-
TP29484	1	18.294	KB882088.1:1616157-1616094	-
TP80284	1	18.977	KB882088.1:1213617-1225499	2:22837433-22849650
TP71655	1	19.93	KB882088.1:754659-754722	-
TP11301	1	22.067	KB882088.1:3657807-3657744	12:26916584-26917098
TP1564	1	22.793	KB871983.1:474678-474741	-
TP88171	1	24.092	KB871983.1:9754-9691	-
TP80632	1	24.585	KB872009.1:119783-119726	-
TP1353	1	25.03	KB871983.1:9609-9672	-
TP88960	1	25.494	KB872009.1:119780-119833	-
TP33141	1	26.858	KB871758.1:283369-286701	-
TP13691	1	27.458	KB872009.1:119833-119780	-
TP4265	1	28.064	KB871807.1:441807-478159	17:52351672-52419433
TP24664	1	28.449	KB871758.1:283369-286701	-
TP4430	1	29.053	-	1:8699866-8699816
TP59088	1	29.847	KB871983.1:302872-302809	-
TP44327	1	30.548	KB872104.1:169012-169075	-
TP90432	1	31.362	KB871983.1:420949-420886	-

TP73658	1	32.414	KB872009.1:268926-268863	-
TP91787	1	33.009	KB872009.1:119726-119783	-
TP63658	1	35.171	-	-
TP53039	1	36.202	KB880314.1:887-824	-
TP19994	1	37.407	KB871926.1:433413-464766	15:27478567-27497198
TP55923	1	38.383	KB871926.1:433413-464766	15:27478567-27497198
TP57439	1	38.891	KB871709.1:317539-317476	-
TP83730	1	40.037	KB882309.1:839397-839334	-
TP68530	1	40.874	KB882309.1:356344-371299	15:27598426-27610782
TP25704	1	41.483	KB871806.1:419285-426938	-
TP57108	1	42.267	KB882309.1:591133-599642	-
TP14503	1	42.974	KB882309.1:769448-769385	-
TP72241	1	43.602	KB882309.1:1157792-1157729	-
TP17021	1	44.798	KB871938.1:2312363-2312300	-
TP71788	1	45.835	KB871938.1:1623388-1623451	-
TP29540	1	46.831	KB882095.1:3705759-3705696	-
TP40956	1	47.597	KB871938.1:3965624-3965561	-
TP33953	1	47.921	KB871938.1:3965540-3965603	-
TP11745	1	49.038	KB882095.1:3705696-3705759	-
TP87802	1	49.364	KB882095.1:3517484-3517547	15:19416291-19416438
TP21844	1	49.872	KB882095.1:2718098-2718161	-
TP78699	1	50.385	KB882095.1:3748394-3748331	13:33290110-33290151
TP24913	1	51.028	KB882095.1:3088682-3098587	15:23010599-23030994
TP66001	1	51.531	KB882095.1:3088682-3098587	15:23010599-23030994
TP50685	1	51.91	KB882095.1:3198873-3236419	15:19100673-19151694
TP75740	1	52.218	KB882095.1:2971302-2971365	-
TP9020	1	53.378	KB882095.1:715191-715131	1:21666836-21666768
TP81261	1	54.179	KB882095.1:855773-855836	24:24231576-24231293
TP32000	1	54.633	KB882095.1:567680-567743	-
TP62747	1	55.016	KB882095.1:464856-464793	-
TP61591	1	55.89	KB882095.1:464782-464845	-

TP4509	1	57.079	KB882095.1:547729-547792	-
TP5430	1	57.501	KB882118.1:1640102-1715755	15:43896267-44024401
TP13531	1	58.107	KB882118.1:1640102-1715755	15:43896267-44024401
TP9059	1	58.67	KB882110.1:1220986-1220923	1:46778477-46778450
TP61391	1	59.071	KB877014.1:2522-2459	-
TP22954	1	59.811	KB882110.1:2467191-2467128	-
TP75766	1	60.913	KB882110.1:1220921-1220984	1:46778477-46778450
TP7472	1	61.928	KB882118.1:1660276-1660213	-
TP42274	1	62.941	KB882118.1:2854150-2854087	-
TP72538	1	63.925	KB882095.1:1557536-1557599	-
TP50040	1	64.737	KB882118.1:1583667-1583730	15:44470227-44468991
TP69159	1	67.004	KB872026.1:53691-53628	9:4652490-4652344
TP83119	1	69.332	KB871613.1:261344-261407	9:1563375-1563509
TP2329	2	0	KB871803.1:233132-233069	-
TP63826	2	1.928	KB872025.1:215685-215748	-
TP46145	2	3.545	-	-
TP22832	2	5.084	-	-
TP88303	2	5.975	KB873096.1:15308-15371	-
TP85253	2	7.441	KB882236.1:427302-470940	-
TP39533	2	8.518	-	-
TP15383	2	9.484	KB872115.1:233505-233442	3:30189111-30189233
TP56369	2	10.397	-	-
TP37817	2	11.153	KB873096.1:291-350	-
TP40987	2	12.653	KB882213.1:1161007-1161070	14:31876631-31876969
TP638	2	13.637	-	-
TP26048	2	14.652	KB872401.1:17386-17441	-
TP20543	2	15.964	KB882213.1:503577-503514	-
TP82219	2	17.165	KB872401.1:17441-17386	-
TP33510	2	17.954	KB872330.1:2662-6570	-
TP26328	2	18.731	KB882142.1:2250284-2250347	14:4758404-4758376
TP47047	2	20.179	KB882142.1:722245-722308	3:31221909-31222025

TP26542	2	21.743	KB871623.1:1066478-1079892	-
TP54536	2	23.537	KB882142.1:1267376-1281216	3:32694591-32702244
TP70720	2	23.925	KB871749.1:808026-807964	12:19171745-19171715
TP82087	2	27.521	-	-
TP4231	2	30.797	KB882110.1:1759604-1759667	-
TP74147	2	32.025	KB882299.1:148413-148476	-
TP60475	2	33.16	KB882118.1:1781917-1781854	15:43611360-43611281
TP59758	2	33.602	KB882299.1:138481-290550	15:16891349-17010825
TP45569	2	35.146	-	-
TP32915	2	36.228	KB882110.1:2874418-2874355	-
TP73145	2	36.772	KB871938.1:1077633-1077570	-
TP14570	2	37.088	KB882299.1:437556-450360	-
TP22175	2	38.586	KB882095.1:1809146-1809209	9:41164786-41164754
TP86990	2	39.614	KB871938.1:3829089-3829026	-
TP90252	2	39.987	KB882118.1:1861191-1861254	-
TP43245	2	40.486	KB882095.1:3748295-3748358	13:33290110-33290151
TP64030	2	41.076	KB882095.1:2133651-2133588	-
TP80064	2	41.979	KB882110.1:2164754-2164817	24:24231142-24231636
TP39782	2	42.788	KB882095.1:2051254-2051317	16:22287642-22287602
TP81309	2	43.509	KB882095.1:1611186-1611249	-
TP63785	2	44.323	KB882095.1:469256-469193	-
TP55533	2	44.753	KB882095.1:3754584-3754521	17:32539802-32539749
TP53395	2	45.396	KB882118.1:2802796-2802733	20:25639866-25639680
TP89944	2	46.057	KB871938.1:2262291-2281973	-
TP79489	2	46.883	KB882095.1:3754496-3754559	17:32539802-32539749
TP66169	2	47.5	KB882095.1:2051373-2051310	15:32059739-32059933
TP74076	2	48.386	KB871709.1:12805-19784	-
TP29245	2	49.22	KB871938.1:4667996-4667933	-
TP36804	2	50.556	KB871911.1:63370-181232	5:38732179-38847817
TP72632	2	51.642	KB882309.1:759455-759518	-
TP69378	2	52.647	KB871758.1:833915-833978	5:62908075-62907675

TP51924	2	53.29	KB871604.1:838453-839499	-
TP83402	2	55.571	KB872003.1:155037-155100	-
TP37468	2	56.016	KB882097.1:1340336-1340273	-
TP80479	2	58.827	KB871831.1:554165-554228	-
TP43415	2	59.885	KB872362.1:19578-61485	-
TP41918	2	60.776	KB871594.1:423110-423047	24:30842888-30842847
TP24540	2	61.542	KB882213.1:192264-192314	-
TP77205	2	61.658	KB871938.1:2082626-2082564	15:3765027-3764861
TP67586	2	61.989	KB872362.1:37825-37762	-
TP73938	2	62.799	KB882160.1:1909120-1909057	17:23462702-23462675
TP56998	2	63.569	-	-
TP43910	2	64.23	KB882270.1:4075570-4075507	-
TP35295	2	65.083	KB882087.1:558787-558850	15:24888448-24888512
TP63167	2	65.625	KB871688.1:831702-831765	-
TP32278	2	66.636	KB872325.1:118885-118948	-
TP45463	2	67.783	KB882097.1:1435746-1435683	-
TP52439	2	69.011	KB882097.1:1340256-1340319	-
TP73890	2	69.491	-	-
TP3063	2	70.602	KB871831.1:309796-309733	-
TP10669	2	78.788	-	-
TP74449	3	-4.047	KB871971.1:348368-468851	4:59603883-59639641
TP68678	3	-2.836	KB872819.1:4781853-4781916	22:24745761-24745709
TP47548	3	0	KB882089.1:1193880-1193817	-
TP47845	3	2.084	KB882229.1:1820923-1831482	22:11977789-11982038
TP79725	3	2.601	KB871610.1:293325-293388	-
TP71843	3	4.321	KB882089.1:1193803-1193866	-
TP21848	3	5.262	KB871716.1:449035-462793	-
TP67473	3	7.44	KB871763.1:475720-484440	25:19498569-19511895
TP76155	3	7.896	KB882283.1:1199466-1199403	-
TP60697	3	8.604	KB871922.1:44943-123187	-
TP12472	3	9.867	KB882287.1:367308-367245	8:13162433-13162398

TP3231	3	10.355	KB882155.1:2196113-2196176	12:26916755-26917024
TP60915	3	10.884	KB882119.1:2139232-2222315	12:43229791-43267962
TP82190	3	11.49	KB882234.1:186370-186307	-
TP41977	3	11.911	KB871728.1:43256-43319	-
TP71666	3	12.556	KB882297.1:506659-506596	-
TP88048	3	12.99	KB871624.1:997178-997241	-
TP49304	3	14.249	KB882193.1:1206968-1207031	-
TP88581	3	14.383	KB871765.1:174293-174356	-
TP7590	3	15.242	KB871728.1:43358-43295	-
TP10475	3	15.616	KB882193.1:345699-345636	24:15442939-15443133
TP78471	3	16.02	KB882202.1:6123590-6123527	-
TP37074	3	16.705	KB882193.1:1835741-1835804	-
TP86834	3	17.242	KB882193.1:1089554-1089491	-
TP41558	3	17.738	KB882297.1:1184737-1232293	20:25388715-25441734
TP86516	3	17.931	KB871876.1:179698-179635	-
TP62048	3	18.553	KB882217.1:343062-342999	-
TP87116	3	19.458	KB882297.1:408738-408675	-
TP69597	3	20.008	KB882278.1:425883-425820	-
TP69017	3	20.124	KB882278.1:425798-425861	-
TP62487	3	20.797	KB882193.1:56159-134756	24:15036261-15167157
TP252	3	21.088	KB872378.1:46947-47010	-
TP35557	3	21.653	KB882288.1:439961-440024	-
TP22562	3	22.041	KB882174.1:1308484-1308421	-
TP23098	3	22.182	KB882221.1:1022495-1022558	-
TP90612	3	22.762	KB882288.1:542618-542555	24:13266659-13267906
TP71391	3	22.977	KB882217.1:619323-619260	-
TP73643	3	23.807	KB882121.1:2186516-2186579	-
TP8265	3	24.427	KB882308.1:769683-769620	8:46980055-46980122
TP90442	3	24.798	KB882202.1:4823380-4823443	-
TP71630	3	25.212	KB877151.1:257-320	-
TP23913	3	25.534	KB871737.1:708117-708054	-

TP23154	3	26.235	KB882202.1:3276194-3276257	-
TP43785	3	26.824	KB882202.1:1016126-1016189	-
TP79009	3	27.199	KB882165.1:451109-451172	-
TP49226	3	27.607	KB882273.1:1010252-1010315	18:15337002-15336972
TP33890	3	27.865	KB882090.1:1737344-1737407	-
TP3447	3	28.489	KB882193.1:95806-95869	-
TP25717	3	28.832	KB882195.1:1417176-1417113	-
TP40740	3	29.677	KB871678.1:59584-59521	18:443440-443517
TP9186	3	30.21	KB871746.1:702040-701977	-
TP75542	3	30.759	KB871647.1:732438-732501	-
TP34045	3	30.979	KB882308.1:496869-496806	-
TP37944	3	33.025	KB882089.1:143429-143366	8:17760789-17760831
TP65858	3	33.553	KB882259.1:1411997-1411934	-
TP516	3	35.011	KB871766.1:619778-619841	24:28055955-28055738
TP59743	3	35.751	KB882155.1:2384862-2384799	-
TP9454	3	36.444	KB882165.1:2397017-2397080	-
TP5775	3	37.446	KB882121.1:2692457-2696486	-
TP56216	3	37.714	KB871728.1:122038-136936	-
TP66514	3	38.69	KB882121.1:457486-457549	24:23865431-23865698
TP47190	3	38.975	KB882259.1:1411852-1411915	-
TP54340	3	40.268	KB882287.1:1000186-1000249	-
TP29553	3	40.897	KB882267.1:1016850-1016787	-
TP22851	3	41.251	KB871953.1:412549-412612	11:44937235-44937675
TP17722	3	41.958	KB882278.1:681173-681236	-
TP66004	3	42.238	KB871876.1:276818-282984	-
TP89440	3	43.39	KB882287.1:1306334-1306397	-
TP75348	3	43.856	KB882165.1:892353-901254	-
TP143	3	44.468	KB882267.1:1113388-1113325	-
TP43072	3	45.135	KB882297.1:161308-177223	20:47198898-47221082
TP2786	3	45.857	KB882174.1:1482105-1482168	-
TP38728	3	46.716	KB882288.1:861817-861754	-

TP30391	3	47.134	KB882174.1:15086-15149	-
TP91924	3	47.321	-	-
TP62697	3	47.629	KB882193.1:1609594-1609531	-
TP52807	3	48.173	KB882193.1:1609520-1609583	-
TP89901	3	49.252	KB882288.1:1309402-1309339	-
TP6301	3	49.844	KB882121.1:3082873-3095188	24:21144508-21163421
TP9693	3	50.785	KB882193.1:1514662-1600496	24:17694937-17765417
TP88568	3	51.156	KB882288.1:861687-861750	-
TP20823	3	51.479	KB882193.1:1514662-1600496	24:17694937-17765417
TP45377	3	51.802	KB882288.1:992776-992713	24:13873327-13873495
TP47831	3	52.24	KB871763.1:475720-484440	25:19498569-19511895
TP60072	3	52.968	KB882165.1:892353-901254	-
TP413	3	53.223	KB882174.1:1706155-1706218	-
TP6636	3	53.514	KB882288.1:1247457-1247520	-
TP88708	3	54.281	KB882278.1:1351129-1351066	-
TP68110	3	54.445	KB882117.1:3200321-3389031	17:17322349-17586436
TP55652	3	54.608	KB882288.1:1247541-1247478	-
TP38906	3	55.305	KB882202.1:5641393-5641330	-
TP64806	3	55.659	KB882165.1:2332279-2335375	-
TP67681	3	55.962	KB882165.1:975202-975265	-
TP42824	3	56.69	KB882202.1:6560-143476	20:43151895-43377812
TP24050	3	56.803	KB882202.1:364091-364154	-
TP78513	3	57.027	KB882247.1:814916-814857	22:24745761-24745717
TP47317	3	57.171	KB882181.1:198796-383092	20:26125137-26389041
TP24708	3	57.774	KB882195.1:1622061-1622124	-
TP79230	3	58.052	KB882221.1:583192-583255	-
TP81761	3	58.333	KB882202.1:638627-638690	-
TP51097	3	58.539	KB882181.1:198796-383092	20:26125137-26389041
TP13373	3	58.99	KB882297.1:1122647-1122710	-
TP66002	3	59.499	KB882234.1:580045-597875	-
TP13211	3	60.325	KB882202.1:476477-476540	11:22633882-22633855

TP42782	3	60.427	KB882311.1:546388-546325	-
TP13363	3	60.825	KB882250.1:1612135-1612198	-
TP40730	3	61.247	KB882089.1:3958577-3958514	-
TP58669	3	61.437	KB882181.1:2088-2025	-
TP55771	3	61.832	KB882250.1:1054856-1054919	-
TP56798	3	62.179	KB882250.1:1054925-1054862	-
TP46196	3	62.641	KB882283.1:881107-881170	-
TP87620	3	63.027	KB871837.1:478048-478111	20:29299059-29298883
TP37406	3	63.618	KB871647.1:917850-917913	2:4349754-4349473
TP44165	3	63.724	KB882250.1:41907-41844	-
TP80141	3	64.056	KB882221.1:583337-583274	-
TP20011	3	64.782	KB872070.1:29647-29584	-
TP4383	3	65.287	KB872060.1:25295-34874	20:34351482-34365791
TP57093	3	65.471	KB871653.1:84927-127652	20:20803212-20885786
TP34665	3	66.255	KB882234.1:1158146-1158083	-
TP21268	3	66.581	KB882221.1:1450469-1450532	-
TP83798	3	67.179	KB882228.1:630960-630897	10:28345569-28345607
TP62364	3	67.701	KB882217.1:483348-502699	-
TP86370	3	68.095	KB871746.1:701938-702001	-
TP45294	3	68.553	KB882228.1:1089273-1089336	18:30423537-30423421
TP64415	3	68.824	KB882090.1:2511283-2511346	-
TP73542	3	69.426	KB882090.1:1512324-1512387	-
TP27957	3	70.098	KB882234.1:971385-986996	-
TP65289	3	70.304	KB882090.1:838059-901517	20:34568138-34667300
TP46325	3	70.836	KB882204.1:1331423-1331360	22:24745722-24745761
TP78941	3	71.359	KB876000.1:15267-15330	-
TP6778	3	72.064	KB882138.1:305199-369581	23:15314866-15441212
TP69790	3	72.819	KB882234.1:1158057-1158120	-
TP5441	3	73.19	KB871653.1:463294-463231	4:29235211-29235136
TP48187	3	73.729	KB882228.1:360296-360359	-
TP88435	3	74.157	KB882273.1:1448044-1473871	20:22202472-22252667

TP29198	3	74.599	KB882302.1:939027-939090	-
TP5138	3	75.156	KB882234.1:550876-559016	5:24376604-24386108
TP43545	3	75.512	KB876000.1:15353-15290	-
TP11209	3	76.004	KB871653.1:79266-79329	-
TP24723	3	76.954	KB882204.1:1483670-1483607	-
TP82073	3	77.554	KB882090.1:1642293-1642356	-
TP4431	3	78.796	KB882228.1:174130-174193	-
TP11322	3	79.809	KB882090.1:2479596-2479659	-
TP40555	3	80.383	KB871624.1:438116-442938	-
TP57203	3	80.844	KB882273.1:1285296-1305185	20:25741325-25764922
TP84349	3	82.026	KB871876.1:198304-198367	-
TP81346	3	82.776	KB882090.1:2572593-2572530	-
TP45973	3	83.241	KB882090.1:2452822-2452885	-
TP76732	3	84.697	-	-
TP8711	3	86.134	KB882273.1:1431312-1431375	-
TP10677	3	88.744	KB871624.1:438116-442938	-
TP70470	3	92.819	KB882234.1:1093117-1093180	-
TP45465	4	0	KB871788.1:131242-174081	-
TP7839	4	1.984	KB882270.1:4959068-5048807	12:35983394-36120397
TP64448	4	3.622	KB882270.1:221098-221035	-
TP50631	4	5.285	KB871788.1:206900-206963	-
TP53614	4	7.112	KB882270.1:1129345-1129282	-
TP56606	4	8.441	KB871850.1:77017-76954	-
TP35244	4	10.402	KB871723.1:678046-677983	-
TP63083	4	12.274	KB882143.1:1724131-1724194	7:50986700-50986768
TP25543	4	16.785	KB882119.1:414953-423991	-
TP71111	4	22.987	KB879810.1:327-367	-
TP77512	4	26.332	KB879810.1:372-327	-
TP43045	4	39.49	KB872187.1:188848-188911	-
TP84626	4	53.145	KB882143.1:1918467-1918530	-
TP82657	4	54.87	KB882143.1:1287391-1335151	12:19604562-19682203

TP59437	4	55.735	KB882231.1:669298-684059	12:12896-19363
TP46744	4	56.575	KB882270.1:1328370-1328307	-
TP62073	4	57.38	KB882143.1:1287391-1335151	12:19604562-19682203
TP34046	4	57.899	KB882270.1:461045-510288	-
TP53135	4	58.654	KB882270.1:4959068-5048807	12:35983394-36120397
TP90645	4	60.01	KB882198.1:1451726-1465268	-
TP78488	4	60.598	KB882231.1:719839-719902	6:40678407-40678118
TP73416	4	61.281	KB871788.1:238434-238371	-
TP82616	4	61.675	KB871788.1:238349-238412	-
TP39826	4	62.519	KB882310.1:1070284-1070221	-
TP87548	4	63.342	KB882143.1:2135297-2140093	12:20444820-20450053
TP74096	4	64.041	KB882285.1:701134-701197	23:754954-754986
TP60213	4	66	KB882119.1:502584-502647	-
TP42595	4	66.904	KB882119.1:1291662-1291599	11:855355-855393
TP90580	4	68.32	KB871761.1:133995-134058	-
TP15353	4	69.219	-	-
TP52987	4	70.027	KB871784.1:738696-759943	12:6803297-6826667
TP73761	4	71.679	KB871784.1:738696-759943	12:6803297-6826667
TP84126	4	74.448	KB871993.1:62921-62858	-
TP88716	4	77.068	KB871846.1:453383-453320	13:50146467-50146018
TP3646	4	79.084	KB871965.1:331856-331793	-
TP3887	4	81.031	KB871965.1:129281-129344	-
TP29735	4	82.585	KB871880.1:14697-14760	-
TP7438	4	85.06	-	-
TP38409	5	0	KB871797.1:634633-634570	-
TP14162	5	1.881	KB882216.1:1334443-1334396	-
TP84535	5	3.819	KB871688.1:2429880-2429818	-
TP35097	5	5.124	KB882160.1:1665769-1674033	-
TP91685	5	5.691	KB871785.1:483296-483233	-
TP61806	5	6.758	KB882216.1:1634035-1805840	-
TP21243	5	7.407	KB882216.1:1410260-1410323	19:17089277-17088939

TP17870	5	7.584	KB882216.1:1334396-1334443	-
TP63399	5	8.217	KB871688.1:550910-550847	-
TP75360	5	8.635	KB882160.1:1295662-1295725	17:44053595-44053485
TP3444	5	9.233	KB872295.1:530052-724797	22:16791095-16980410
TP12995	5	9.568	KB882292.1:398814-398751	-
TP29514	5	10.01	KB871698.1:31533-31470	-
TP63837	5	10.212	KB874407.1:1032-2382	-
TP38353	5	10.858	KB872105.1:223677-223740	14:44943395-44943366
TP6832	5	11.143	KB871956.1:66107-66170	-
TP21181	5	11.559	KB882249.1:1695762-1695706	-
TP3893	5	11.905	KB872033.1:371215-371152	-
TP30107	5	12.44	KB871900.1:100629-100692	-
TP20410	5	12.775	KB871698.1:208117-208180	-
TP49879	5	13.187	KB882160.1:778434-778497	-
TP76841	5	13.496	KB882160.1:34154-34091	-
TP28832	5	13.885	KB882249.1:651049-650986	-
TP61290	5	14.24	KB882160.1:476764-589247	22:2566694-2578110
TP48847	5	14.671	KB871797.1:596768-596705	-
TP31617	5	15.139	KB882292.1:881915-881852	-
TP56425	5	15.393	KB871797.1:121099-129059	22:41377617-41388011
TP86265	5	15.684	KB882292.1:203006-203069	12:17248896-17249155
TP66782	5	16.049	KB871875.1:193823-223604	11:35326937-35355113
TP66289	5	16.496	KB882292.1:203074-203011	12:17248896-17249155
TP9169	5	16.713	KB871900.1:364709-364646	-
TP56411	5	17.142	KB882159.1:1082-1019	19:14708239-14708202
TP43026	5	17.514	KB882160.1:476764-589247	22:2566694-2578110
TP51481	5	17.855	KB882159.1:444417-444480	-
TP20994	5	18.065	KB871970.1:137202-137265	-
TP52771	5	18.296	KB872033.1:350791-350729	1:31370674-31370536
TP2743	5	18.403	KB871797.1:538006-537943	-
TP29134	5	18.502	KB871900.1:67037-206061	7:75361777-75382186

TP40428	5	18.869	KB872033.1:350729-350791	1:31370674-31370536
TP43343	5	19.263	KB882159.1:214455-280791	22:41869267-41959136
TP38870	5	19.433	KB871797.1:537925-537988	-
TP43051	5	19.693	KB871875.1:193823-223604	11:35326937-35355113
TP44920	5	20.074	KB871797.1:436976-458775	-
TP88083	5	20.192	KB882159.1:21791-22930	-
TP74555	5	20.464	KB872033.1:49983-50046	2:9000197-9000122
TP68553	5	20.899	KB882081.1:3224286-3388831	1:59883467-59884178
TP74885	5	21.217	KB871956.1:29523-29586	-
TP74961	5	21.399	KB882160.1:1909021-1909084	17:23462702-23462675
TP55682	5	21.817	KB871698.1:19555-19492	19:35003609-35003649
TP19309	5	22.195	KB871875.1:241259-241196	-
TP86211	5	22.519	KB872033.1:54974-54911	-
TP57571	5	22.879	KB871900.1:371454-371517	18:30423418-30423537
TP90790	5	23.163	-	-
TP7726	5	23.639	KB872295.1:4048107-4048170	-
TP76900	5	23.89	KB871753.1:230425-230488	-
TP4787	5	24.2	KB882223.1:1719612-1719675	22:24745766-24745709
TP76344	5	24.77	KB871753.1:1558056-1557993	22:24554954-24555073
TP56958	5	24.928	KB872033.1:219234-219171	-
TP5264	5	25.314	KB871785.1:394332-403709	-
TP68543	5	25.648	KB882097.1:193842-229133	-
TP75118	5	25.929	KB882097.1:878472-878535	-
TP83684	5	26.116	KB882097.1:193842-229133	-
TP90329	5	26.358	KB871688.1:599197-599260	17:35849430-35849477
TP7414	5	26.81	KB882091.1:981592-981655	7:67027775-67027545
TP18958	5	27.193	KB871688.1:2599362-2615987	7:58352183-58369985
TP83311	5	27.775	KB871688.1:3191565-3191628	7:54649925-54649784
TP9505	5	28.165	KB882097.1:878589-878526	-
TP65842	5	29.004	KB882091.1:981679-981616	7:67027775-67027545
TP46234	5	29.708	KB871753.1:1199103-1199040	10:30445253-30445031

TP83657	5	30.001	KB882097.1:226644-226707	-
TP20264	5	30.681	KB882191.1:2017514-2017451	-
TP40961	5	31.192	KB882191.1:608355-608292	19:16585287-16585198
TP90863	5	36.696	KB871797.1:749392-749455	-
TP872	5	38.313	-	-
TP20977	5	38.879	KB871959.1:412501-412564	-
TP88687	5	40.5	KB872295.1:3906609-3906672	-
TP79499	5	41.089	KB882223.1:603553-603616	-
TP58136	5	43	KB871688.1:895652-895715	-
TP13595	5	45.789	KB872621.1:3584-3521	-
TP33640	5	46.498	KB872485.1:4381-4444	-
TP52748	5	47.06	KB882160.1:157736-157673	19:16585269-16585212
TP75819	5	48.569	KB871588.1:805584-805625	25:26106488-26106547
TP12431	5	48.798	KB882159.1:209517-209454	-
TP82894	5	49.256	KB871698.1:706314-706377	-
TP63899	5	49.895	KB871956.1:29629-29566	-
TP41506	5	50.579	KB882159.1:700150-700087	-
TP21236	5	51.29	KB871970.1:101829-101766	25:29483429-29483402
TP19039	5	51.958	KB882292.1:53230-53167	-
TP35860	5	52.28	KB871956.1:183433-183370	9:55568663-55568633
TP82448	5	53.266	KB871956.1:183366-183429	9:55568663-55568633
TP64587	5	54.101	KB871753.1:3380616-3380679	-
TP76733	5	54.331	KB882160.1:379506-412271	-
TP64931	5	54.752	KB882249.1:1071873-1183696	7:12024586-12157760
TP87937	5	55.458	-	-
TP38670	5	55.914	KB871753.1:1569333-1569396	-
TP13959	5	56.353	KB882223.1:1764638-1794725	22:38827165-38865579
TP14748	5	56.964	KB882223.1:1042003-1042066	-
TP9070	5	57.49	KB871588.1:805625-805584	25:26106488-26106547
TP84386	5	57.845	KB871797.1:596647-596710	-
TP3531	5	58.771	KB871956.1:714-777	-

TP81831	5	59.32	KB882160.1:611921-611858	-
TP58187	5	59.546	KB872295.1:4060958-4061021	-
TP63240	5	59.966	KB871753.1:3395233-3412549	-
TP23534	5	60.548	KB871753.1:2879100-2879163	-
TP55795	5	60.843	KB871753.1:3073257-3073194	-
TP39690	5	61.337	KB871875.1:473748-473811	17:44894357-44893884
TP63095	5	61.84	KB871753.1:3073171-3073234	-
TP69307	5	62.198	KB871753.1:4111595-4111658	4:21130707-21130623
TP52929	5	62.934	KB871753.1:4431213-4431276	7:34893101-34893277
TP17345	5	63.385	KB871785.1:369747-369684	-
TP24736	5	64.051	KB871924.1:294559-294622	17:34262365-34262416
TP59045	5	64.634	KB882097.1:3300353-3300416	-
TP54704	5	65.136	KB882097.1:1516779-1516842	-
TP74840	5	65.742	KB871753.1:2766545-2766608	18:30423454-30423499
TP21482	5	66.223	KB871795.1:375439-375376	2:38106111-38106186
TP80252	5	67.011	KB882097.1:1666868-1666805	-
TP91440	5	67.352	KB872295.1:2840798-2840861	-
TP46588	5	68.185	KB872089.1:39662-39601	-
TP71379	5	68.652	KB872295.1:3463466-3463529	-
TP59641	5	69.056	KB872295.1:4447902-4447839	-
TP70040	5	69.642	KB882191.1:2095117-2095054	-
TP43506	5	70.579	KB871785.1:369667-369730	-
TP49749	5	70.791	KB871795.1:15242-15305	7:68743488-68743658
TP55234	5	71.735	-	-
TP64391	5	72.266	KB871924.1:131277-131340	-
TP17595	5	72.625	KB882097.1:2105529-2105592	-
TP49279	5	73.363	KB872295.1:3299801-3299738	-
TP22762	5	75.089	KB882191.1:1416619-1416556	-
TP82164	5	75.689	KB882216.1:1321047-1320984	-
TP81818	5	76.327	KB871795.1:375195-420028	-
TP36411	5	77.067	KB882191.1:1416543-1416606	-

TP30832	5	79.25	KB882216.1:1078661-1078598	7:69353711-69353565
TP87245	5	79.873	KB882216.1:1711196-1711259	-
TP56561	5	81.409	KB882216.1:1078584-1078647	7:69353711-69353565
TP65853	5	83.722	KB871998.1:324025-329291	-
TP22558	5	87.965	KB871740.1:442933-442996	-
TP9703	5	90.197	KB871740.1:443012-442949	-
TP56648	5	94.488	KB871740.1:507053-507005	-
TP56910	6	0	KB871684.1:35923-190268	8:16794178-16925840
TP37532	6	8.757	KB872147.1:160849-160898	-
TP64457	6	11.553	KB871595.1:598435-598498	-
TP13568	6	13.357	KB872147.1:241954-241891	-
TP44958	6	14.69	KB871595.1:598527-598464	-
TP88002	6	16.492	KB871839.1:394884-425859	-
TP43630	6	17.121	KB871913.1:488106-488043	6:10356713-10356683
TP71240	6	18.456	KB871688.1:5644627-5644564	-
TP35069	6	19.391	-	-
TP74734	6	20.569	KB882094.1:3724742-3724679	-
TP80276	6	21.68	KB871682.1:346821-346884	-
TP55061	6	21.975	KB871595.1:855220-893738	8:12857501-12914333
TP83937	6	23.367	KB882113.1:932801-932864	-
TP46777	6	24.059	KB871601.1:568530-568467	-
TP16419	6	24.378	KB882211.1:1205140-1205203	-
TP14674	6	25.045	KB882094.1:1433074-1433137	-
TP32666	6	25.746	KB882113.1:2407790-2407730	Zv9_NA423:8272-8355
TP48201	6	26.353	KB872252.1:58784-74113	8:55366298-55382248
TP18651	6	26.76	KB882306.1:967641-967704	19:16585198-16585251
TP13292	6	27.886	KB871601.1:573172-573235	-
TP81812	6	28.576	KB871823.1:5755-12732	17:20793630-20809230
TP61506	6	29.684	KB882113.1:103117-128475	8:51076270-51107959
TP70015	6	30.707	KB882289.1:51733-123876	8:54270456-54369808
TP49604	6	31.243	KB882289.1:51733-123876	8:54270456-54369808

TP87408	6	31.65	-	-
TP58270	6	32.123	KB882113.1:103117-128475	8:51076270-51107959
TP20034	6	32.601	KB882094.1:531759-531696	-
TP4380	6	32.901	KB871601.1:326742-326805	8:11437968-11438127
TP76960	6	33.808	KB882094.1:531678-531741	-
TP52504	6	37.043	KB882211.1:354622-354559	15:44896240-44897183
TP43578	6	37.79	KB871688.1:5378029-5377966	-
TP72796	6	38.813	KB882211.1:1648321-1666701	-
TP79686	6	39.507	KB882211.1:1648321-1666701	-
TP12560	6	41.199	KB871688.1:4302948-4303011	-
TP49566	6	42.188	KB871688.1:4857414-4861753	-
TP21291	6	43.166	KB871688.1:5377940-5378003	-
TP54605	6	43.758	KB882183.1:233813-233874	18:10457457-10457429
TP32455	6	44.84	KB882105.1:1955095-1955158	-
TP17003	6	45.536	KB882105.1:2255352-2255415	8:32749629-32749747
TP59283	6	46.316	KB871601.1:1170635-1170572	-
TP75749	6	46.678	-	-
TP58322	6	47.934	KB882105.1:2255429-2255366	8:32749629-32749747
TP77142	6	48.243	KB882105.1:1280786-1280723	-
TP25766	6	48.631	KB873810.1:9400-10533	-
TP90779	6	49.162	KB882105.1:2500282-2500219	-
TP89271	6	49.549	KB882105.1:2775247-2775184	8:4198377-4198505
TP57291	6	50.604	KB871607.1:831418-831355	-
TP68734	6	50.71	KB871601.1:916829-916892	19:17088950-17089276
TP11198	6	51.015	KB871607.1:36484-36421	-
TP75195	6	51.559	KB882105.1:340633-351550	-
TP8285	6	52.602	KB871607.1:831341-831404	-
TP53860	6	53.092	KB871607.1:128896-128959	-
TP49235	6	53.37	KB871601.1:382625-412688	8:13441358-13464125
TP2381	6	54.054	KB871944.1:444292-444355	-
TP11757	6	54.786	KB871794.1:667894-667831	17:30836652-30836612

TP18170	6	55.153	KB872495.1:32659-32722	-
TP65353	6	55.232	KB871794.1:741472-741535	-
TP68863	6	56.152	KB871823.1:35190-35127	-
TP42861	6	56.576	KB882113.1:437608-437671	-
TP31990	6	57.591	KB871979.1:155355-157136	-
TP9955	6	57.743	KB871595.1:713091-713028	-
TP703	6	58.187	KB882113.1:285065-285002	8:50494961-50494847
TP80097	6	58.466	KB871817.1:403084-425803	8:24492067-24509673
TP87771	6	59.194	KB871939.1:4662142-4662205	-
TP62839	6	59.754	KB871595.1:679514-679577	19:20910597-20910561
TP54976	6	59.861	KB872505.1:93594-93531	-
TP55753	6	60.811	-	-
TP57684	6	60.982	KB882113.1:587396-587333	19:174779-174747
TP17618	6	61.398	KB882113.1:932912-932849	-
TP44130	6	62.51	KB881055.1:960-897	-
TP55613	6	63.116	KB882113.1:2745873-2745936	-
TP91499	6	63.229	KB871595.1:679632-679569	19:20910597-20910561
TP9829	6	63.692	KB872176.1:159708-162824	-
TP59956	6	64.005	KB871879.1:267267-267204	-
TP75880	6	64.841	KB871879.1:206197-206255	-
TP45166	6	65.395	KB871879.1:207761-207698	-
TP22610	6	65.417	KB871595.1:1101518-1101581	-
TP75359	6	66.134	KB871879.1:267189-267252	-
TP39630	6	66.685	KB871939.1:1847616-1965178	8:15309781-15335728
TP21613	6	67.325	KB872252.1:58784-74113	8:55366298-55382248
TP91154	6	67.996	KB871595.1:1096-53256	-
TP83978	6	68.006	KB872176.1:159708-162824	-
TP64133	6	68.202	KB872252.1:58784-74113	8:55366298-55382248
TP50712	6	68.739	KB882113.1:2745973-2745910	-
TP77170	6	69.803	KB871939.1:5106509-5106446	-
TP52355	6	70.501	KB871939.1:2096153-2110315	-

TP41094	6	70.615	KB871595.1:427879-427816	-
TP65405	6	71.198	KB882113.1:2596582-2596645	-
TP10493	6	71.928	KB871939.1:2293925-2392863	22:34925835-35026071
TP55580	6	72.239	KB871939.1:2561229-2561166	22:24745709-24745761
TP69016	6	73.124	KB871939.1:4662268-4662205	-
TP53643	6	73.897	KB871939.1:2774787-2914562	-
TP79970	6	74.151	KB871939.1:2293925-2392863	22:34925835-35026071
TP84343	6	74.828	KB871939.1:1703420-1703483	-
TP52017	6	75.422	KB871939.1:883744-891919	-
TP50887	6	76.004	KB871894.1:183951-184014	-
TP69655	6	76.589	KB882094.1:3721646-3721709	-
TP4461	6	76.898	KB871879.1:298934-298997	-
TP70458	6	77.269	KB871939.1:317305-317368	-
TP25490	6	78.011	KB871939.1:317369-317306	-
TP69099	6	79.102	KB882094.1:196357-196294	-
TP35464	6	80.099	KB871939.1:420481-420418	3:42013298-42013241
TP26813	6	82.02	KB871939.1:769448-769511	-
TP5002	6	83.079	KB871939.1:2774787-2914562	-
TP84868	6	84.445	KB882094.1:261593-261656	-
TP63568	6	86.002	KB882094.1:1561276-1561339	-
TP53856	6	87.591	KB871939.1:769580-769517	-
TP61000	6	91.243	KB882094.1:2546700-2546637	-
TP91984	6	93.633	KB882094.1:1486511-1486448	-
TP66087	6	94.807	KB882094.1:2012053-2011990	17:18994063-18994036
TP18199	7	0	KB882280.1:1284628-1284687	24:6062205-6061732
TP71482	7	0.49	KB871934.1:82871-82808	-
TP65272	7	0.821	KB871706.1:274727-274664	-
TP10360	7	1.251	KB871706.1:206171-206234	24:39308904-39308950
TP30988	7	2.317	KB882102.1:596955-596898	11:32558804-32559024
TP3406	7	2.584	KB882162.1:2031526-2031463	4:4853579-4853440
TP9993	7	3.71	KB871706.1:12858-12921	-

TP28292	7	4.602	KB882162.1:738661-738724	-
TP55249	7	5.814	KB882280.1:1284687-1284628	24:6062205-6061732
TP79613	7	6.726	KB871620.1:834721-834658	22:15518553-15518525
TP88045	7	6.817	KB874330.1:2819-4304	4:10013913-10022598
TP31336	7	7.289	KB871934.1:237630-237567	-
TP22592	7	7.806	KB882280.1:804708-804771	-
TP74243	7	8.313	KB882280.1:48258-48321	-
TP81893	7	8.814	KB871934.1:250084-250021	-
TP38190	7	9.684	KB882162.1:738737-738674	-
TP37753	7	10.688	KB882162.1:2237359-2237296	22:24745717-24745761
TP40545	7	10.914	KB871706.1:27043-26980	4:6758351-6758535
TP46079	7	11.393	KB882280.1:13657-13720	-
TP70877	7	11.788	KB871934.1:51926-51863	-
TP71934	7	12.74	KB871620.1:980237-980174	-
TP81686	7	13.081	KB882087.1:4045437-4045374	-
TP48166	7	13.869	KB871620.1:575594-575531	22:41380992-41381337
TP31777	7	14.666	KB871620.1:839200-839263	-
TP5991	7	15.064	KB871833.1:875109-1012481	25:20244007-20355181
TP63063	7	15.784	KB871833.1:584441-584504	13:37549306-37549277
TP11052	7	16.886	KB871713.1:675954-676017	-
TP38531	7	17.267	KB871833.1:1456900-1456837	11:20292486-20292452
TP69637	7	17.959	-	-
TP27366	7	19.257	KB872029.1:25595-37130	-
TP32497	7	19.781	KB882209.1:1292397-1292334	-
TP35194	7	20.203	KB871735.1:262559-262496	4:20167797-20167954
TP65419	7	20.68	KB871730.1:521364-562968	4:21360143-21408343
TP34271	7	21.355	-	-
TP79670	7	21.773	KB871894.1:136510-146851	8:18875502-18887136
TP82311	7	22.363	KB872049.1:119951-120014	-
TP78499	7	22.873	KB871857.1:519097-519160	5:63164954-63164693
TP30226	7	23.815	KB871857.1:474499-556370	4:19748590-19923254

TP58401	7	24.235	KB871971.1:424388-424451	-
TP45833	7	24.894	KB871857.1:474499-556370	4:19748590-19923254
TP27793	7	24.999	KB872868.1:23991-24054	-
TP80612	7	25.362	KB872177.1:233853-233916	-
TP76657	7	25.522	KB871812.1:364074-364109	24:39308950-39308911
TP69811	7	26.62	KB872562.1:56398-56335	-
TP61003	7	26.905	KB871971.1:424502-424439	-
TP64244	7	27.337	KB871735.1:262482-262545	4:20167797-20167954
TP30682	7	28.155	KB882087.1:247004-247067	-
TP30413	7	28.7	KB872029.1:25595-37130	-
TP20453	7	29.556	KB871617.1:972483-972420	-
TP61117	7	30.479	KB871768.1:127174-127237	-
TP44340	7	31.265	KB871583.1:763760-763823	-
TP62696	7	32.86	KB882280.1:44786-44723	5:26040848-26040876
TP41200	7	33.31	KB882280.1:44682-44745	5:26040848-26040876
TP46466	7	34.893	KB871833.1:4939237-4939300	-
TP83407	7	36.272	KB871857.1:270243-270180	-
TP6172	7	37.105	KB871681.1:519988-519925	-
TP9075	7	38.243	KB882109.1:3110003-3109948	-
TP25250	7	39.225	KB871834.1:232802-232865	-
TP75903	7	46.732	KB882162.1:260727-260664	11:38566794-38567223
TP73989	7	47.495	KB873866.1:4902-4839	-
TP57803	7	47.585	KB873866.1:4792-4855	-
TP63601	7	47.762	KB882280.1:119241-119178	22:6430296-6430141
TP79862	7	48.057	KB882280.1:605141-605204	-
TP34543	7	49.333	KB871713.1:285885-285948	-
TP22574	7	50.031	KB871893.1:370276-370213	-
TP73954	7	50.243	-	-
TP52239	7	50.952	KB871857.1:343280-343343	-
TP50864	7	51.477	KB882162.1:519603-519667	-
TP12809	7	51.842	KB871706.1:559600-559537	10:44130449-44130418

TP89353	7	52.749	KB871857.1:343357-343294	-
TP61775	7	53.211	KB882087.1:4016891-4016954	4:13005740-13005853
TP24706	7	53.762	KB882209.1:589340-589403	-
TP56961	7	54.234	KB882087.1:223802-703826	23:278938-287938
TP65027	7	54.935	KB882087.1:223802-703826	23:278938-287938
TP36616	7	55.262	KB882087.1:1315988-1315925	-
TP8620	7	55.82	KB871620.1:575591-575654	22:41381050-41381337
TP89682	7	56.803	KB871934.1:82721-82784	-
TP73347	7	57.243	KB882162.1:340965-340902	24:13629590-13629560
TP51381	7	57.744	KB882087.1:3173878-3216290	4:18646376-18676721
TP1388	7	58.172	KB871583.1:49337-49400	-
TP62041	7	58.584	KB882189.1:1635929-1635870	-
TP42579	7	59.615	KB871834.1:2584282-2624980	25:5992926-6024305
TP38513	7	60.106	KB882082.1:6725155-6725092	Zv9_scaffold3556:38635-38693
TP27425	7	60.679	KB882082.1:6725074-6725137	Zv9_scaffold3556:38635-38693
TP64737	7	61.336	KB871834.1:2584282-2624980	25:5992926-6024305
TP80537	7	61.805	KB882156.1:75642-82317	-
TP42596	7	62.306	KB882156.1:75642-82317	-
TP80162	7	63.163	KB882082.1:6803582-6887360	7:58153142-58351210
TP82083	7	64.462	KB882087.1:2028611-2028674	-
TP41105	7	66.661	KB882100.1:2617412-2617349	18:30423268-30423535
TP7340	7	67.511	KB882087.1:2028712-2028649	-
TP61971	7	68.528	KB871579.1:8856671-8856608	-
TP67641	7	69.061	KB882100.1:28087-28150	-
TP78238	7	69.813	KB871834.1:3867049-3866986	-
TP18896	7	70.726	KB882087.1:711239-775815	4:17277222-17349254
TP81820	8	0	KB882239.1:413655-413592	-
TP50488	8	5.434	KB873380.1:2810-2879	25:38282945-38283173
TP21816	8	7.591	-	-
TP42433	8	9.644	KB880947.1:1015-1078	-
TP78605	8	13.027	KB882175.1:446423-446486	-

TP8518	8	15.41	KB882258.1:1484226-1484163	-
TP72096	8	17.057	KB882258.1:1484119-1484182	-
TP40180	8	18.556	KB882258.1:1434666-1434603	16:22287612-22287642
TP83	8	20.112	KB871579.1:249234-249297	14:2390720-2391441
TP87763	8	24.291	KB871579.1:1992744-1992807	5:28412596-28412872
TP58897	8	25.45	KB871763.1:475720-484440	25:19498569-19511895
TP28553	8	26.345	KB871579.1:1833224-1833169	-
TP54887	8	27.166	KB871579.1:1833169-1833224	-
TP47479	8	28.51	KB871579.1:3094845-3094908	5:23787433-23787406
TP47776	8	29.787	KB871763.1:475720-484440	25:19498569-19511895
TP52618	8	30.467	KB871579.1:3239228-3239291	12:16679420-16679351
TP30305	8	31.754	KB871579.1:3921201-3921264	-
TP1247	8	32.657	KB871579.1:3318001-3318064	-
TP80180	8	33.704	KB871579.1:4250472-4250409	-
TP47919	8	34.732	KB871579.1:4625551-4625604	Zv9_NA503:31350-31323
TP13657	8	35.554	KB871579.1:4467953-4467890	-
TP32305	8	36.094	KB871579.1:3362408-3362471	-
TP55224	8	36.912	KB871579.1:4467867-4467930	-
TP39485	8	37.559	KB871579.1:4625604-4625551	Zv9_NA503:31350-31323
TP8074	8	39.964	KB871579.1:7619745-7619808	-
TP89515	8	40.711	KB871579.1:8066809-8066872	Zv9_NA101:20073-19664
TP80609	8	41.412	KB871579.1:9119575-9119638	-
TP57124	8	42.327	KB871579.1:9117320-9117381	5:26040883-26040849
TP58317	8	43.683	KB871579.1:8270204-8270141	-
TP5664	8	43.851	KB871579.1:9191756-9191819	-
TP29626	8	44.398	KB871579.1:7619763-7630248	-
TP66817	8	44.791	KB871579.1:7699314-7711437	25:3507866-3527071
TP71216	8	45.726	KB871579.1:8856537-8856600	-
TP70200	8	46.798	KB871579.1:9117381-9117320	5:26040883-26040849
TP55437	8	47.841	KB882100.1:1803726-1818327	25:33294061-33341195
TP37687	8	48.659	KB882100.1:2679360-2702927	25:28610997-28679514

TP11080	8	49.682	KB882100.1:1806069-1806006	-
TP56241	8	50.272	KB882100.1:3247199-3247136	25:29911302-29911506
TP50068	8	51.851	KB882082.1:5628718-5628655	-
TP62896	8	52.434	KB882082.1:5079627-5079564	-
TP32198	8	53.375	KB871630.1:4698393-4723079	1:23343890-23368434
TP65662	8	54.327	KB882082.1:5241330-5253733	-
TP50230	8	55.036	KB882082.1:4495766-4495703	-
TP9180	8	56.321	KB882082.1:5241330-5253733	-
TP19607	8	57.258	KB882082.1:4767504-4767441	-
TP40136	8	57.983	KB871681.1:205510-205573	-
TP60996	8	59.334	KB882082.1:5180612-5180549	-
TP36567	8	60.546	KB871681.1:285658-285721	-
TP86867	8	61.998	KB871834.1:3723013-3722950	-
TP12501	8	63.114	KB871681.1:159654-159717	-
TP27097	8	65.659	KB871681.1:372874-372937	-
TP22579	8	67.061	KB871834.1:2584282-2624980	25:5992926-6024305
TP80809	9	0	-	-
TP75765	9	2.78	KB872976.1:17300-17363	-
TP7145	9	7.191	KB882243.1:654473-654410	17:44053382-44053757
TP83705	9	12.031	KB882265.1:915749-915812	-
TP24534	9	13.6	KB873319.1:33094-33145	-
TP72434	9	15.274	KB882265.1:455895-465338	-
TP80772	9	15.719	KB871627.1:777194-777131	7:62132584-62132653
TP43399	9	16.766	-	-
TP55764	9	17.483	KB871600.1:398477-398414	-
TP80125	9	18.418	KB882084.1:992904-1003826	-
TP89434	9	19.252	KB882084.1:859529-859592	11:20231194-20231280
TP81070	9	20.571	KB882265.1:1201821-1201884	-
TP20039	9	21.771	KB882236.1:865766-865829	-
TP81424	9	22.739	KB872091.1:108444-108507	5:28412679-28412514
TP40035	9	23.312	KB872454.1:5037-5100	Zv9_NA4:6782-6397

TP50825	9	23.915	KB882084.1:452448-452385	4:23917438-23917498
TP20240	9	24.714	-	-
TP18579	9	24.983	-	-
TP36124	9	25.606	-	-
TP15355	9	26.073	-	-
TP29263	9	26.595	KB882084.1:2142625-2142688	-
TP71948	9	27.262	KB882084.1:2142761-2142698	-
TP46326	9	27.83	KB882281.1:366600-366663	-
TP39644	9	28.244	KB882084.1:1940918-1952384	17:19311202-19327159
TP29002	9	28.731	KB882084.1:2932924-2932987	15:42502758-42502811
TP10587	9	29.657	KB882084.1:3600295-3600358	-
TP56098	9	30.031	KB882084.1:2933017-2932954	15:42502758-42502811
TP61558	9	30.381	KB882084.1:4338081-4338144	8:11062655-11062600
TP87967	9	31.054	KB882153.1:1832494-1832431	-
TP74204	9	31.617	KB882153.1:1832387-1832450	-
TP67459	9	32.134	KB882153.1:417760-417823	-
TP47822	9	32.675	KB882153.1:472796-472859	17:36972586-36972212
TP19149	9	33.174	KB882153.1:56808-56745	-
TP47971	9	34.232	KB882158.1:848983-849046	-
TP31790	9	35.026	KB882179.1:1928342-1928405	-
TP41664	9	35.535	KB871726.1:543942-549861	-
TP45203	9	35.944	KB871726.1:424645-442410	17:45176230-45195289
TP46433	9	36.666	KB882158.1:1924740-1924677	-
TP63778	9	37.15	KB882158.1:1464967-1559322	17:38770308-38882444
TP50753	9	38.167	KB871726.1:424645-442410	17:45176230-45195289
TP71065	9	38.296	KB882158.1:840641-840578	-
TP77505	9	39.386	KB882179.1:1671782-1671845	17:42008839-42009148
TP77653	9	39.683	KB882179.1:1404085-1404022	-
TP6677	9	40.343	KB882150.1:827474-827411	-
TP2900	9	41.101	KB882233.1:6157953-6212655	-
TP43326	9	41.916	KB882233.1:5957400-5962876	17:2955959-2962753

TP63316	9	42.386	KB882179.1:278210-278147	7:44728048-44727931
TP17220	9	42.984	KB882084.1:77062-77125	-
TP18246	9	43.306	KB871600.1:396715-396778	-
TP82112	9	43.715	KB882150.1:1581012-1589760	-
TP75526	9	44.312	KB882179.1:59652-59589	15:15145687-15145643
TP26778	9	45.474	KB882233.1:5957400-5962876	17:2955959-2962753
TP53676	9	46.008	KB882150.1:1581012-1589760	-
TP47693	9	47.292	KB882265.1:1034640-1055460	17:28143502-28161099
TP60908	9	47.766	KB882265.1:1034640-1055460	17:28143502-28161099
TP3143	9	48.38	KB882236.1:766605-766668	5:38441264-38441319
TP67593	9	48.861	KB882150.1:318393-318330	-
TP29851	9	49.292	KB872310.1:88749-108386	-
TP52902	9	50.081	KB882117.1:509240-509303	19:31702303-31702276
TP40382	9	50.611	KB882233.1:3040699-3040762	-
TP87718	9	51.176	KB882117.1:487921-487984	1:14199917-14200332
TP56532	9	52.381	KB882117.1:2271036-2271099	19:29084484-29084444
TP92005	9	52.753	KB882117.1:1290851-1290788	-
TP26660	9	53.784	KB882257.1:173287-173224	-
TP83346	9	54.624	KB882117.1:2783100-2783037	-
TP25071	9	55.45	KB872047.1:133673-133736	17:18691703-18691568
TP38413	9	55.936	KB882117.1:488013-487950	1:14199917-14200332
TP54945	9	56.5	KB882233.1:2209366-2256054	17:51756133-51785695
TP31289	9	56.956	KB882233.1:934630-934693	-
TP20635	9	57.443	KB871991.1:269749-269801	5:26040883-26040848
TP74981	9	58.169	KB882117.1:1215054-1215117	-
TP36252	9	58.406	KB871991.1:269801-269749	5:26040883-26040848
TP91062	9	60.332	KB871991.1:226734-226797	-
TP90715	9	62.676	KB872358.1:2417-2480	-
TP90493	9	63.323	KB871726.1:220130-240316	17:45580840-45619632
TP49160	9	64.14	KB871726.1:220130-240316	17:45580840-45619632
TP500	9	64.594	KB882233.1:5345397-5345460	-

TP63265	9	65.777	KB871726.1:261657-280806	17:45515242-45545989
TP38411	9	67.102	KB872091.1:19757-19694	-
TP81517	9	67.557	KB882084.1:2341871-2341934	-
TP15679	9	68.254	KB882158.1:489726-489663	-
TP75250	9	68.932	KB882233.1:1500927-1500990	-
TP65637	9	70.396	KB882233.1:3292187-3292250	-
TP29610	9	71.376	KB882233.1:5660176-5678771	20:874892-917683
TP8663	9	72.932	KB882233.1:3231390-3231327	-
TP9471	9	73.877	KB882084.1:1940918-1952384	17:19311202-19327159
TP10325	9	74.914	KB882117.1:2239106-2239043	17:16408132-16408394
TP5752	9	75.561	KB882233.1:115202-115139	-
TP30937	9	76.665	KB882117.1:3280725-3280788	17:17398712-17398859
TP72093	9	77.525	KB872195.1:75773-132194	25:33983333-34104360
TP34380	9	79.826	KB871991.1:61646-61709	-
TP36338	9	81.045	-	-
TP17842	9	82.436	KB871655.1:395747-395684	-
TP37844	9	83.18	KB882117.1:2233339-2233276	9:4652497-4652461
TP38511	9	84.678	KB871695.1:337491-337428	19:20910596-20910561
TP53666	9	86.586	KB871695.1:337425-337488	19:20910596-20910561
TP42446	9	88.723	KB871695.1:881764-901468	17:19250142-19293508
TP54037	9	90	KB871695.1:881764-901468	17:19250142-19293508
TP1052	9	92.594	KB871695.1:718908-718845	4:28296143-28296283
TP27087	10	0	KB872295.1:4206419-4206447	-
TP65624	10	1.993	KB872150.1:179469-179406	-
TP56771	10	4.781	-	-
TP158	10	9.079	KB872082.1:910473-910410	-
TP61798	10	10.406	KB872082.1:3005817-3005754	-
TP48596	10	11.414	KB872082.1:2613201-2613264	-
TP54896	10	12.468	KB872082.1:1390168-1390105	-
TP74570	10	13.316	KB872082.1:824643-824706	-
TP7784	10	14.243	KB872082.1:3177794-3177731	-

TP80201	10	14.894	KB872082.1:3968388-3968325	-
TP11799	10	15.739	KB872082.1:3571273-3571219	-
TP65857	10	16.58	KB872082.1:3571219-3571273	-
TP33691	10	17.566	KB872082.1:3968303-3968366	-
TP76625	10	18.415	KB872082.1:2521115-2521178	24:2392288-2392164
TP70701	10	18.982	KB872082.1:4500290-4500353	-
TP78791	10	19.992	KB872082.1:3177714-3177777	-
TP80723	10	20.218	KB872082.1:4407813-4614141	24:377386-402286
TP36151	10	20.851	KB875994.1:13439-13376	-
TP75420	10	21.861	KB872011.1:295434-318313	16:58232595-58259363
TP29709	10	22.641	KB871754.1:509494-509431	17:31262739-31262704
TP43536	10	23.094	KB871754.1:935919-935982	14:34983896-34983862
TP42286	10	23.922	KB882127.1:47325-47388	-
TP12179	10	25.487	KB882127.1:1320903-1320966	7:70676892-70676920
TP28811	10	26.029	KB882127.1:47407-47344	-
TP55297	10	26.407	KB882127.1:1256281-1293086	16:32826766-32866687
TP43864	10	27.118	KB882133.1:2374373-2374436	-
TP76235	10	27.941	KB882127.1:2189189-2189126	-
TP8980	10	28.468	KB882127.1:1200525-1200588	-
TP54316	10	28.922	KB882127.1:1004746-1004809	-
TP20482	10	29.176	KB882127.1:1266236-1266173	-
TP81256	10	29.623	KB876249.1:323-386	-
TP36867	10	30.109	KB876249.1:405-342	-
TP3790	10	30.875	KB882127.1:1256281-1293086	16:32826766-32866687
TP52606	10	31.441	KB882133.1:2577434-2577371	-
TP82273	10	32.147	KB882133.1:2122049-2122101	-
TP70185	10	32.443	KB882127.1:2224418-2224355	-
TP18455	10	33.03	KB882133.1:2577313-2577376	-
TP55889	10	33.594	KB882133.1:537494-537557	-
TP88531	10	34.436	KB882133.1:784874-784937	24:31444126-31444235
TP31803	10	35.024	KB882133.1:718945-732497	-

TP8783	10	35.333	KB882126.1:649446-649509	-
TP82218	10	35.617	KB882133.1:2340426-2340489	-
TP63908	10	35.889	KB882133.1:2374491-2374428	-
TP81358	10	36.976	KB882127.1:1154500-1154563	16:33006719-33006666
TP61744	10	37.601	KB882127.1:1984307-1984244	-
TP88242	10	38.988	KB882127.1:2243181-2243244	-
TP28111	10	39.603	KB871754.1:2056223-2056286	22:24745709-24745766
TP65472	10	40.389	KB882133.1:1542617-1542554	-
TP85906	10	41.584	KB882133.1:892519-892582	24:31572122-31572167
TP27055	10	42.25	KB882148.1:101671-101734	-
TP33178	10	43.289	KB882148.1:101786-101723	-
TP91507	10	43.947	KB882126.1:2967529-2967466	-
TP42289	10	45.034	KB882126.1:3138851-3138914	-
TP24718	10	46.194	KB882148.1:1485999-1485936	16:22287642-22287612
TP32715	10	46.671	KB882169.1:915566-921930	13:406179-417379
TP19546	10	47.352	KB882188.1:920997-920934	23:19858266-19858234
TP76501	10	48.366	KB882188.1:920905-920968	23:19858266-19858234
TP21421	10	49.572	KB882148.1:1886561-1886624	-
TP60018	10	50.617	KB882232.1:539046-539109	3:32183143-32183215
TP51499	10	51.301	KB882232.1:1384846-1384783	-
TP37368	10	52.044	KB882232.1:1131592-1131529	-
TP9046	10	52.791	KB882226.1:710928-718395	19:9834567-9851048
TP84537	10	53.61	KB882226.1:1230225-1230162	-
TP47198	10	55.072	KB882226.1:1230154-1230217	-
TP88432	10	56.139	KB872410.1:66294-74849	-
TP56934	10	56.565	KB871700.1:185587-185524	3:3673753-3673780
TP55074	10	57.34	KB871700.1:185480-185543	3:3673753-3673780
TP57569	10	58.677	KB871885.1:151815-185126	-
TP41843	10	59.709	KB871690.1:665630-665567	-
TP50771	10	60.983	KB871700.1:174655-174592	24:24231636-24231143
TP35109	10	62.526	KB871691.1:729514-729451	-

TP19535	10	63.279	KB871594.1:988322-988385	-
TP65337	10	66.262	KB882224.1:1256502-1256565	-
TP29629	10	67.262	KB882224.1:14471-14408	-
TP7924	10	68.162	KB872335.1:19754-165403	-
TP43309	10	68.959	KB871789.1:572067-572130	-
TP37418	10	69.653	KB871789.1:618205-618142	-
TP26240	10	70.229	KB872286.1:108329-108266	-
TP38527	10	70.802	KB872286.1:133723-133660	-
TP80210	10	71.385	KB871814.1:480211-480148	-
TP38306	10	72.094	KB872532.1:26477-26414	23:40786458-40786550
TP90422	10	72.674	KB872256.1:74360-74423	-
TP62326	10	73.36	KB871910.1:411742-411803	-
TP52365	10	73.884	KB872449.1:61083-91600	-
TP26786	10	74.825	KB872449.1:61083-91600	-
TP43719	10	75.596	KB872256.1:74444-74381	-
TP78911	10	76.517	KB871910.1:411803-411742	-
TP36936	10	77.347	KB872335.1:14612-14675	-
TP40201	10	78.164	KB882087.1:2764557-2764496	8:30638220-30638177
TP34391	10	78.78	KB882219.1:1909772-1971631	16:18091182-18164707
TP72346	10	80.281	KB872256.1:125532-125469	-
TP20886	10	80.857	KB872449.1:69605-69542	-
TP62184	10	83.381	KB882219.1:236585-236648	-
TP76600	10	85.165	KB882219.1:359738-359675	-
TP19549	11	0	KB872082.1:541462-541399	-
TP3563	11	4.419	KB872011.1:295434-318313	16:58232595-58259363
TP57531	11	6.406	KB871754.1:2365604-2365541	12:16080742-16080685
TP77776	11	8.298	KB871754.1:2365538-2365601	12:16080742-16080685
TP56161	11	9.056	-	-
TP27451	11	9.847	KB882127.1:1321058-1321121	7:70676892-70676920
TP81768	11	10.773	KB882127.1:1967186-1967123	-
TP80825	11	12.245	KB882127.1:502183-502120	5:34604677-34604740

TP86761	11	13.051	KB882127.1:1967107-1967170	-
TP83740	11	14.579	KB882148.1:1656897-1656834	-
TP70721	11	15.136	KB882188.1:2099588-2105442	-
TP53782	11	15.515	KB871690.1:280768-280705	-
TP20665	11	16.59	KB871961.1:41206-41143	19:22534712-22534770
TP75930	11	17.695	KB882224.1:433876-440637	-
TP65153	11	18.379	KB871594.1:422966-423029	-
TP13974	11	19.234	KB871691.1:430338-430401	22:24745794-24745717
TP43865	11	20.196	-	-
TP33046	11	20.859	KB872850.1:32263-32326	-
TP35218	11	22.014	KB882139.1:2268013-2278798	-
TP52144	11	22.338	KB882139.1:968376-968439	-
TP79576	11	23.019	KB871814.1:242344-242407	24:35603005-35602551
TP30003	11	23.719	KB872481.1:62667-62730	-
TP61434	11	24.686	KB871910.1:194430-201204	-
TP22058	11	25.923	KB882219.1:1373673-1373736	-
TP20177	11	26.981	KB882219.1:1618145-1618082	-
TP88776	11	28.145	KB882139.1:1513920-1513983	-
TP56833	11	31.183	KB882224.1:1499569-1499506	-
TP66038	12	0	KB871906.1:28375-28438	-
TP70940	12	4.087	KB872729.1:5466-5403	-
TP76331	12	8.235	KB871906.1:30367-30304	1:59235174-59235214
TP51687	12	10.814	KB882301.1:705531-705468	1:31370588-31370503
TP19932	12	15.129	KB871612.1:1018456-1018408	-
TP2487	12	17.263	KB871612.1:1018408-1018456	-
TP53009	12	18.549	KB872681.1:57454-57513	17:32921481-32921509
TP56536	12	20.818	KB871612.1:409991-410054	-
TP78048	12	22.511	-	-
TP9805	12	22.95	KB871612.1:345445-441937	1:54829728-54941279
TP5204	12	24.556	KB871612.1:410081-410018	-
TP22520	12	25.709	KB882301.1:1281095-1281032	3:61381507-61381545

TP68685	12	27.553	KB882101.1:1294661-1294625	1:29417092-29416936
TP55242	12	30.478	KB882101.1:1530868-1530805	-
TP47586	12	31.73	KB882101.1:3554094-3554031	-
TP39546	12	33.845	KB882144.1:1042446-1042509	-
TP63739	12	35.246	KB882168.1:2317630-2317693	-
TP53430	12	36.411	KB882168.1:732284-732347	1:6237230-6237202
TP11356	12	37.489	KB882168.1:691358-727999	-
TP46127	12	38.326	KB882168.1:691358-727999	-
TP22341	12	39.497	KB882168.1:2317699-2317636	-
TP47250	12	40.773	KB873087.1:5855-5908	3:52275064-52275012
TP32941	12	41.385	KB882144.1:1042524-1042461	-
TP61763	12	42.707	KB882101.1:3780898-3780835	-
TP31924	12	43.737	KB872729.1:5340-5403	-
TP21163	12	43.769	KB871589.1:327945-376604	9:56096420-56133921
TP23446	12	45.291	KB882269.1:494397-494460	-
TP78705	12	46.091	KB882144.1:1322475-1322412	-
TP30103	12	46.355	-	-
TP15847	12	46.961	KB882194.1:670037-670100	-
TP64305	12	48.007	KB871977.1:305580-305517	-
TP77049	12	48.545	KB871977.1:317965-318028	4:23917498-23917228
TP3844	12	49.866	KB871977.1:318040-317977	4:23917498-23917228
TP29700	12	51.091	KB871906.1:387239-404890	1:59697247-59722698
TP18641	12	51.241	KB882293.1:318726-318789	Zv9_scaffold3466:2490-2319
TP72637	12	52.492	KB871999.1:255963-255900	-
TP60118	12	52.921	KB882293.1:1060944-1168137	1:7381076-7477583
TP46794	12	53.84	KB871705.1:893290-893353	-
TP82809	12	54.678	KB882293.1:128375-128312	1:8081096-8080964
TP2957	12	55.456	KB882293.1:346387-346450	-
TP41258	12	56.568	KB872122.1:247400-247463	-
TP18742	12	57.603	KB872122.1:247532-247469	-
TP50113	12	58.214	KB876503.1:1099-1036	-

TP41974	12	59.343	KB876503.1:1024-1087	-
TP9847	12	60.356	KB872045.1:15804-22571	-
TP68467	12	60.787	KB871994.1:147108-147045	-
TP44288	12	61.747	KB871845.1:93995-93932	22:24745766-24745722
TP49041	12	63.086	KB872045.1:146559-146622	6:39976073-39975958
TP55483	12	64.447	KB882220.1:1034493-1087035	21:4514314-4562968
TP66786	12	66.204	KB871826.1:308807-308870	12:30331054-30331372
TP80071	12	66.664	KB876503.1:896-959	-
TP8926	12	66.947	KB871826.1:308874-308811	12:30331054-30331372
TP45857	12	69.111	KB871845.1:103469-103532	-
TP49921	12	70.026	KB871999.1:61813-61876	-
TP43495	12	71.343	KB872269.1:68373-68310	-
TP38245	12	74.156	KB871769.1:668572-668510	-
TP70491	12	78.247	KB871769.1:231457-231520	-
TP74601	12	79.653	KB871845.1:325217-325154	-
TP90947	12	80.364	KB871845.1:325146-325209	-
TP55354	12	81.11	-	-
TP57484	12	81.741	KB871994.1:392106-392043	-
TP24614	12	82.037	-	-
TP51593	12	82.875	KB871705.1:49321-49384	-
TP37664	12	84.015	KB871769.1:231544-231481	-
TP25958	12	84.549	KB882144.1:234966-234903	1:47203622-47203751
TP65865	12	85.67	KB871999.1:258646-258583	-
TP74644	12	86.474	KB882194.1:1812630-1812567	17:42642889-42643062
TP20582	12	87.931	KB882144.1:234872-234935	1:47203622-47203751
TP37896	12	89.905	KB882293.1:316149-316086	17:44893933-44894357
TP65046	13	-0.514	KB882171.1:395911-409242	6:42222718-42268405
TP42311	13	0	KB882171.1:4903672-4903609	8:17760865-17760804
TP48469	13	1.329	KB882122.1:3178745-3178808	-
TP7964	13	1.969	KB882235.1:651486-651549	-
TP91162	13	2.202	KB882171.1:4993916-4993979	-

TP20111	13	2.381	KB871802.1:164135-164072	-
TP48266	13	2.882	KB882152.1:1207248-1207185	-
TP84247	13	3.183	KB879591.1:868-805	24:899942-899974
TP80408	13	3.606	KB872671.1:115935-118819	3:46482738-46490382
TP70668	13	4.154	KB882171.1:4460409-4460472	3:1241526-1241554
TP66354	13	4.617	KB871670.1:82219-82156	-
TP28771	13	5.11	KB882122.1:2798793-2798856	24:7762729-7762441
TP73580	13	5.252	KB882171.1:1289540-1289603	-
TP52991	13	5.574	KB882115.1:2638977-2639040	-
TP82982	13	6.098	KB882185.1:1598612-1598675	-
TP13841	13	6.438	KB882122.1:3178814-3178751	-
TP90742	13	6.855	KB882115.1:3180979-3180916	-
TP64729	13	7.298	KB882253.1:1023155-1023218	-
TP19424	13	7.509	KB882082.1:1008734-1016462	-
TP56580	13	7.997	KB882122.1:915725-915788	6:36295809-36295985
TP74596	13	8.41	KB882115.1:2351072-2351135	-
TP72239	13	8.634	KB882115.1:3001183-3202266	6:29838528-29924174
TP57173	13	8.882	KB882152.1:1098679-1098742	13:21770972-21770945
TP19356	13	9.157	KB882230.1:1206944-1290897	6:7472429-7573834
TP50450	13	9.556	KB882122.1:915807-915744	6:36295809-36295985
TP15350	13	10.06	KB882253.1:190722-190785	6:33109266-33109171
TP6054	13	10.398	KB882152.1:286989-287052	6:40468917-40468804
TP50196	13	10.79	KB882230.1:55936-55873	7:62132584-62132653
TP17600	13	11.158	KB882115.1:1818341-1818404	-
TP87700	13	11.429	KB882115.1:1433750-1433687	-
TP72044	13	11.568	KB882115.1:1433674-1433737	-
TP37173	13	12.1	KB882152.1:287075-287012	6:40468917-40468804
TP75388	13	12.311	KB882162.1:2122829-2122766	-
TP1795	13	12.722	KB882082.1:157932-157869	-
TP73133	13	12.975	KB882176.1:1547543-1547480	-
TP17149	13	13.135	KB882115.1:1818410-1818347	-

TP7828	13	13.395	KB882230.1:53471-64639	-
TP41979	13	13.936	KB882120.1:1201440-1269076	6:11183233-11273818
TP23915	13	14.115	KB871764.1:639990-640053	3:50575197-50575170
TP49559	13	14.36	KB882120.1:352809-454001	6:11948143-12084297
TP39567	13	14.63	KB882176.1:1654612-1665509	-
TP30121	13	15.034	KB882176.1:1654612-1665509	-
TP36297	13	15.272	KB881948.1:593-538	9:4652386-4652490
TP12006	13	15.568	KB872173.1:113691-113722	-
TP73757	13	15.818	KB871651.1:717985-717922	-
TP62949	13	16.031	KB882256.1:1081998-1092852	6:52163393-52176108
TP89714	13	16.281	KB882120.1:50171-50108	-
TP69132	13	16.832	KB871932.1:36170-36107	-
TP79575	13	17.349	KB882120.1:616714-616651	6:11754464-11754342
TP68210	13	17.95	KB882172.1:537323-537260	-
TP73315	13	18.233	KB871764.1:478454-478517	-
TP71477	13	18.851	-	-
TP14030	13	18.975	KB882256.1:933238-980996	6:51815743-51984937
TP89581	13	20.192	KB871670.1:825296-853938	6:23272530-23300991
TP74176	13	22.479	KB882171.1:5057621-5065264	-
TP40991	13	24.938	KB882256.1:56024-109271	6:16426420-16495588
TP1775	13	26.234	KB882256.1:1668982-1672538	-
TP51602	13	27.069	KB882256.1:56024-109271	6:16426420-16495588
TP9356	13	27.596	KB882120.1:1201440-1269076	6:11183233-11273818
TP79065	13	28.114	-	-
TP77005	13	29.53	KB882171.1:2009622-2009685	-
TP84838	13	29.837	KB882256.1:1706353-1714701	-
TP63188	13	30.503	KB871932.1:195786-195723	-
TP6843	13	30.858	KB882120.1:385903-385966	-
TP82136	13	31.507	KB882168.1:1540430-1557362	1:31160640-31176847
TP55360	13	31.727	KB871651.1:829048-829111	15:7679763-7679791
TP65539	13	32.26	KB882120.1:241479-241416	-

TP52486	13	32.892	KB882301.1:177962-184147	15:1886681-2000232
TP14170	13	33.344	KB871651.1:829131-829068	15:7679763-7679791
TP7248	13	34.057	KB871670.1:829092-829155	24:21435926-21435967
TP29485	13	34.577	KB882115.1:1287379-1287442	-
TP39833	13	35.107	KB872111.1:59383-59446	-
TP19373	13	35.506	KB872818.1:4248066-4248003	-
TP33057	13	36.25	KB882082.1:694619-716777	6:4048461-4090070
TP52539	13	36.539	KB882082.1:694619-716777	6:4048461-4090070
TP1258	13	38.055	KB871815.1:602877-602828	-
TP27200	13	38.628	KB882161.1:1001147-1001084	3:50575169-50575211
TP15822	13	39.166	KB882082.1:3276638-3276701	9:47053421-47053581
TP55367	13	39.572	KB882082.1:2708084-2708147	-
TP86560	13	39.918	KB882161.1:1323822-1323759	-
TP9229	13	40.077	KB882122.1:2135244-2135307	9:55478164-55477616
TP82208	13	40.674	KB882176.1:1920359-1920422	-
TP76975	13	41.147	KB882082.1:3262822-3262759	-
TP91510	13	41.715	KB872595.1:22045-21982	6:27635539-27635015
TP42242	13	42.342	KB882176.1:1633339-1633402	-
TP5756	13	42.788	KB871651.1:526427-526490	-
TP26423	13	42.984	KB871651.1:563451-563388	-
TP54637	13	43.007	KB882082.1:3276725-3276662	9:47053421-47053581
TP87709	13	43.124	-	24:18921730-18921674
TP16577	13	43.408	-	-
TP81058	13	43.927	KB882120.1:50071-50134	-
TP73789	13	44.271	KB882120.1:64805-64742	-
TP91947	13	44.921	KB882161.1:2737455-2737392	6:58316050-58316377
TP347	13	45.486	KB872372.1:118786-118849	-
TP58182	13	45.704	KB872062.1:411521-411458	-
TP82191	13	46.343	KB882120.1:2360852-2360915	-
TP39682	13	46.779	-	-
TP31009	13	47.24	KB882082.1:1260532-1260469	18:30423537-30423434

TP12314	13	47.575	-	-
TP39703	13	47.923	KB882176.1:1633615-1633552	-
TP22399	13	48.247	KB882171.1:3966809-3966746	14:29170935-29170869
TP40485	13	48.453	KB872372.1:118245-118185	-
TP60305	13	49.083	KB882235.1:86992-87055	6:29470531-29470504
TP35350	13	49.288	KB882159.1:2734707-2734767	7:62132653-62132584
TP17672	13	49.44	KB882082.1:1316097-1316034	-
TP53063	13	49.576	KB882171.1:3090745-3090808	-
TP55244	13	50.079	KB882082.1:702840-702777	-
TP41633	13	50.382	KB882176.1:1669996-1670059	25:29483445-29483402
TP6228	13	50.891	KB882171.1:3966712-3966775	14:29170935-29170869
TP23120	13	51.068	KB871878.1:224743-224680	14:18820983-18821043
TP46496	13	51.342	KB872035.1:163867-163804	-
TP75781	13	51.666	KB882235.1:501187-501124	7:10961451-10961582
TP24172	13	52.023	KB871665.1:702619-702682	-
TP28008	13	52.276	KB882172.1:2420411-2420352	13:28047557-28047512
TP29421	13	52.571	KB882172.1:2420352-2420411	13:28047557-28047512
TP64634	13	52.866	KB871670.1:676042-675979	24:899974-899946
TP59665	13	53.177	KB882235.1:38110-49985	9:57612045-57628299
TP38498	13	53.666	KB882082.1:77776-77713	-
TP88907	13	54.028	KB882185.1:1347463-1360201	6:40856558-40878533
TP86367	13	54.072	KB882082.1:1367355-1367292	-
TP18575	13	54.536	KB872044.1:45300-45237	-
TP90880	13	55.026	KB872044.1:45158-45221	-
TP88787	13	55.203	KB871670.1:675960-676023	24:899974-899946
TP33526	13	55.406	KB882152.1:822405-822342	-
TP79264	13	55.656	KB882122.1:33262-33325	-
TP71406	13	56.073	KB882185.1:417701-433207	6:39599715-39631385
TP64674	13	56.366	KB882185.1:1347463-1360201	6:40856558-40878533
TP19732	13	56.496	KB882172.1:15398-15335	-
TP15116	13	57.189	KB882152.1:814511-814448	-

TP89798	13	57.366	KB882185.1:1410417-1410480	8:4866614-4866653
TP63374	13	57.528	-	-
TP20769	13	57.956	KB882115.1:1180507-1180570	-
TP80514	13	58.317	KB882171.1:2798940-2798877	-
TP74136	13	58.675	KB882082.1:644812-644749	-
TP77861	13	59.13	KB882152.1:822300-822363	-
TP78629	13	59.256	KB882253.1:1002611-1002674	14:47613485-47613450
TP44190	13	59.951	KB882172.1:1942166-1942229	22:24745766-24745709
TP20296	13	60.294	KB882115.1:514736-514673	13:33290107-33290153
TP80316	13	60.539	KB882172.1:828956-828893	3:32183121-32183309
TP20976	13	60.902	KB882230.1:1397878-1397815	-
TP20956	13	61.21	KB882253.1:1120495-1120432	-
TP7656	13	61.51	KB872111.1:235271-235208	12:15418053-15418164
TP48041	13	61.791	KB882230.1:55839-55902	7:62132584-62132653
TP4370	13	62.067	-	13:30849005-30849061
TP64988	13	62.671	KB882171.1:5906529-5906592	-
TP29140	13	62.86	KB882230.1:1206944-1290897	6:7472429-7573834
TP15205	13	63.053	KB882115.1:1989290-1989227	24:38054269-38054296
TP58663	13	63.439	KB882115.1:2335108-2335045	20:29809911-29809982
TP7720	13	63.595	KB882230.1:1224292-1224229	-
TP30661	13	64.058	KB882122.1:549391-549328	7:62859850-62859823
TP67127	13	64.38	KB882185.1:417701-433207	6:39599715-39631385
TP88433	13	64.81	KB871802.1:219634-219697	-
TP43879	13	65.163	KB882171.1:2798835-2798898	-
TP82366	13	65.676	KB882253.1:547428-547365	-
TP1170	13	66.542	KB882185.1:99321-99258	-
TP59763	13	66.99	KB882122.1:3120728-3120791	-
TP31572	13	67.412	KB882122.1:2883798-2883861	-
TP5779	13	68.099	KB882230.1:1627870-1627807	-
TP82537	13	68.494	KB882230.1:1224187-1224250	-
TP42458	13	69.14	KB882115.1:514635-514698	13:33290107-33290153

TP27893	13	69.595	KB882253.1:547345-547408	-
TP59985	13	70.494	KB882115.1:734069-734132	7:10962779-10962739
TP89918	13	70.925	KB882253.1:1392333-1392270	15:13386711-13386739
TP61793	13	71.663	KB882115.1:719806-719743	-
TP16833	13	72.206	KB882115.1:1810453-1810390	-
TP89336	13	73.695	KB882185.1:816127-816064	-
TP31187	13	74.561	KB882152.1:1312832-1335353	-
TP54964	13	75.774	KB882171.1:3706866-3706929	-
TP27527	13	76.415	KB882122.1:1386944-1386881	-
TP28663	13	77.721	KB882152.1:1312832-1335353	-
TP57668	13	79.619	KB882171.1:6572496-6590793	6:58770954-58804193
TP79528	14	0	KB882129.1:822449-822386	-
TP81504	14	1.524	KB882163.1:2615644-2615707	17:40760708-40760628
TP65410	14	2.602	KB882220.1:1177783-1262492	23:232228-265517
TP19527	14	3.59	KB871781.1:622875-622812	-
TP79644	14	3.925	KB882129.1:937529-937592	18:10457459-10457429
TP89565	14	4.858	-	-
TP91155	14	5.291	KB882129.1:822351-822414	-
TP86274	14	5.995	KB882096.1:618159-618096	21:10501672-10501604
TP36807	14	7.036	KB877858.1:1811-1748	1:16007109-16007063
TP70451	14	7.397	KB871783.1:12052-11989	-
TP65520	14	8.133	KB872107.1:95000-95063	-
TP78231	14	8.642	KB871642.1:43864-103440	-
TP76510	14	9.432	KB882104.1:959975-959912	-
TP68656	14	9.721	KB882104.1:448014-587120	22:9717905-9727337
TP24628	14	9.895	KB882104.1:995792-1016563	-
TP53209	14	10.602	KB882096.1:2878767-2878704	-
TP19556	14	10.882	KB882096.1:3246604-3246541	-
TP31610	14	11.383	KB882096.1:2878683-2878746	-
TP32329	14	11.8	KB871781.1:195741-195678	-
TP12606	14	12.464	KB871955.1:259852-259915	-

TP25286	14	12.882	KB882275.1:981617-981680	-
TP82804	14	13.136	KB871685.1:107463-107400	21:35250453-35250528
TP32142	14	13.841	KB882104.1:3874328-3874391	-
TP54589	14	14.324	KB882220.1:1177783-1262492	23:232228-265517
TP33294	14	14.685	KB882104.1:3409922-3409859	-
TP14081	14	15.045	KB882275.1:337913-337976	-
TP80876	14	15.802	KB871656.1:75035-75098	21:1166556-1166718
TP29292	14	16.124	KB882104.1:3932723-3932786	1:40718506-40718790
TP9783	14	16.812	KB882104.1:995792-1016563	-
TP22903	14	17.476	KB871642.1:541204-541267	14:31876634-31877131
TP31762	14	17.668	KB882083.1:113484-113421	-
TP57809	14	18.295	KB882105.1:1995435-2013289	8:32233446-32241471
TP41629	14	18.779	KB882129.1:1802805-1822218	17:32364748-32539865
TP36991	14	19.377	KB882083.1:4095370-4095307	-
TP71155	14	26.813	KB882083.1:1280369-1280432	Zv9_scaffold3560:39237-39313
TP17203	14	29.633	KB872107.1:136826-136889	-
TP89189	14	30.461	KB871650.1:31585-31648	21:38215753-38215639
TP64861	14	31.213	KB882096.1:2718406-2718469	20:53876426-53876385
TP29607	14	31.806	KB871781.1:441024-440961	1:42737356-42737152
TP29532	14	32.198	KB871650.1:31660-31597	21:38215753-38215639
TP74903	14	32.603	KB872063.1:49548-49611	-
TP53142	14	33.099	KB872063.1:49633-49570	-
TP21406	14	33.813	KB871650.1:944684-944747	13:10748600-10748548
TP91235	14	34.374	KB882096.1:2718483-2718420	20:53876426-53876385
TP82720	14	34.848	KB871781.1:189712-189649	20:51700857-51700800
TP36287	14	35.048	-	-
TP55299	14	35.593	KB871650.1:313103-313166	-
TP70603	14	36.526	KB871642.1:541352-541289	14:31876634-31877131
TP16958	14	37.294	KB882220.1:526303-526362	-
TP44273	14	37.808	KB871742.1:502948-502885	-
TP14243	14	38.823	KB882220.1:526362-526303	-

TP75027	14	39.173	KB882220.1:1531585-1531522	21:5331658-5331832
TP36682	14	39.888	-	-
TP56654	14	40.392	KB882096.1:489237-489300	-
TP34019	14	40.818	KB871783.1:423434-423492	-
TP15648	14	41.382	KB882220.1:718127-718064	-
TP65969	14	41.548	KB882220.1:1204216-1204279	2:2956026-2956074
TP58362	14	42.311	KB882104.1:759653-803975	8:40684179-40720555
TP16128	14	42.982	KB882104.1:438286-438223	-
TP19276	14	43.274	KB882104.1:759653-803975	8:40684179-40720555
TP8656	14	43.868	KB871783.1:423492-423434	-
TP19704	14	44.353	KB882104.1:1242532-1242469	-
TP47353	14	44.892	KB882104.1:3237018-3237081	-
TP323	14	45.182	KB882096.1:832584-832521	4:23917366-23917502
TP60415	14	45.867	KB882104.1:3109323-3109260	12:45015100-45015228
TP64470	14	46.334	KB871685.1:677110-706438	21:34293174-34344131
TP54990	14	47.091	KB882083.1:2162617-2162554	-
TP74001	14	47.527	KB882083.1:3590829-3590892	-
TP40920	14	48.021	KB882096.1:1291167-1291230	-
TP48614	14	48.268	KB876177.1:4293-4230	-
TP48137	14	49.171	KB882083.1:2552430-2552367	-
TP11007	14	49.576	KB882083.1:2719813-2719876	-
TP10863	14	49.876	KB882104.1:1410631-1449516	-
TP17640	14	50.3	KB876177.1:4172-4235	-
TP88183	14	50.553	KB882096.1:1748140-1833994	21:8195493-8278837
TP76873	14	50.748	KB882083.1:2719915-2719852	-
TP22247	14	51.438	KB882083.1:1965756-1965693	12:49926876-49926794
TP88252	14	51.843	KB882083.1:3710892-3710829	-
TP76512	14	52.38	KB882083.1:3826059-3860854	21:31099916-31112612
TP50911	14	52.721	KB882083.1:3668137-3673600	14:6912871-6919881
TP34561	14	53.161	KB882083.1:2486067-2486004	-
TP3223	14	53.881	KB882142.1:503208-544464	3:31077483-31092481

TP36815	14	54.449	KB882083.1:4124253-4124316	-
TP90688	14	54.964	KB882275.1:1344201-1358668	21:37705353-37755396
TP18197	14	55.152	KB882275.1:830885-830948	-
TP81661	14	56.004	KB882275.1:815279-815216	-
TP36667	14	56.804	KB882304.1:379044-378981	21:27068641-27068851
TP16955	14	56.935	-	-
TP27088	14	58.153	KB882181.1:1385961-1385898	-
TP33729	14	58.564	KB882304.1:743032-742969	-
TP89033	14	59.528	-	-
TP43819	14	60.02	KB882129.1:2197836-2197773	21:22568110-22568440
TP26014	14	60.319	KB882275.1:815185-815248	-
TP43870	14	61.027	KB882184.1:1096600-1096537	-
TP78354	14	61.435	KB882129.1:937663-937600	18:10457459-10457429
TP88925	14	62.016	KB882163.1:2615778-2615715	17:40760708-40760628
TP62733	14	63.793	KB882163.1:1790229-1790166	22:24745755-24745723
TP53977	14	64.479	-	-
TP45863	14	65.317	-	-
TP72955	14	65.992	KB871861.1:116476-116413	1:52423437-52423558
TP42496	14	67.853	KB882163.1:1368105-1368168	22:24745721-24745761
TP56949	14	69.11	KB882163.1:1843943-1898361	21:15563063-15618633
TP17723	14	69.828	KB882163.1:2071254-2071191	1:45760755-45760582
TP40569	14	71.723	KB882163.1:1580692-1580755	1:57205068-57205214
TP69948	14	73.762	KB882163.1:1589932-1589869	-
TP5857	14	76.008	KB882163.1:884541-884604	13:21770944-21770972
TP16403	15	0	-	-
TP85444	15	4.355	KB872239.1:14824-14761	-
TP25335	15	5.362	KB872175.1:129878-129941	-
TP91771	15	7.776	KB871870.1:50441-50378	-
TP90156	15	8.201	KB872001.1:179291-179232	-
TP68995	15	8.682	KB882096.1:4047582-4077543	-
TP61716	15	10.054	KB873385.1:10142-10205	9:45413799-45413852

TP52499	15	10.746	KB882096.1:4307036-4306973	9:54297522-54298500
TP51988	15	11.26	-	-
TP4235	15	12.235	KB872075.1:210144-238304	23:4764287-4802992
TP5341	15	12.528	KB872021.1:58793-58856	-
TP67696	15	14.16	KB871907.1:377410-377473	19:16585212-16585291
TP25532	15	14.226	KB871870.1:50365-50428	-
TP44268	15	14.814	KB871907.1:377477-377414	19:16585212-16585291
TP87118	15	16.725	KB871886.1:150673-150610	5:73625314-73624578
TP48527	15	16.827	-	-
TP15307	15	17.745	KB871886.1:243464-243401	9:4652341-4652497
TP14179	15	18.126	-	-
TP55202	15	18.804	KB871886.1:243396-243459	9:4652341-4652497
TP43652	15	20.207	KB882102.1:60384-60447	-
TP51717	15	21.192	-	-
TP67133	15	21.586	KB871835.1:368189-370555	-
TP2520	15	22.88	KB882102.1:313244-323351	23:28041156-28063557
TP64679	15	23.13	KB882102.1:313244-323351	23:28041156-28063557
TP50108	15	23.659	KB871835.1:369790-369853	-
TP2278	15	24.832	KB882102.1:934609-934546	-
TP31684	15	25.42	KB882102.1:120413-124515	23:27882108-27888406
TP45426	15	26.056	KB882102.1:120413-124515	23:27882108-27888406
TP70190	15	26.781	KB872077.1:112561-112498	22:26752625-26752669
TP45911	15	27.731	KB882102.1:762866-784702	-
TP23620	15	28.209	KB872077.1:160545-160482	-
TP44378	15	29.319	KB872077.1:152816-152879	-
TP63760	15	30.104	KB882102.1:1279171-1316684	23:28737093-28811700
TP80318	15	30.313	KB882102.1:1279171-1316684	23:28737093-28811700
TP53883	15	31.518	KB882102.1:1469099-1490126	23:28936170-28956874
TP79744	15	32.242	KB882102.1:2115663-2221297	23:29641872-29748355
TP27039	15	32.882	KB882102.1:1279171-1316684	23:28737093-28811700
TP63249	15	34.221	-	-

TP33561	15	34.52	KB882102.1:2115663-2221297	23:29641872-29748355
TP3946	15	36.185	KB882262.1:853699-853762	-
TP52027	15	36.928	KB882098.1:301078-305217	-
TP32988	15	37.641	KB882098.1:785944-785881	-
TP48760	15	38.203	KB882098.1:2247717-2247780	23:22804452-22804228
TP21159	15	38.41	KB882128.1:241118-279465	23:20550120-20598055
TP61735	15	39.512	KB871724.1:490946-491009	-
TP65570	15	40.073	KB882128.1:1177567-1177630	17:18307981-18308025
TP91765	15	40.578	KB871724.1:687152-687089	Zv9_NA808:14412-14323
TP88727	15	41.557	KB882098.1:2649782-2649719	12:6993586-6993613
TP3364	15	42.153	KB882098.1:2737840-2737777	24:24231161-24231632
TP39901	15	42.903	KB882138.1:3031225-3060790	23:16802135-16812899
TP56835	15	43.405	KB882138.1:3031225-3060790	23:16802135-16812899
TP68921	15	43.91	KB882128.1:2322497-2322560	-
TP88934	15	44.968	KB882128.1:3002772-3062345	23:17182172-17272078
TP18304	15	45.127	-	-
TP9513	15	46.062	KB882138.1:87646-87583	-
TP71928	15	46.585	KB882175.1:433755-441315	Zv9_scaffold3556:4519-13947
TP7832	15	47.34	KB871980.1:284086-284023	18:37343290-37343239
TP50830	15	47.806	KB871790.1:149512-149575	-
TP3379	15	48.568	KB871724.1:577875-596689	-
TP74800	15	49.15	KB876217.1:6805-6868	-
TP72871	15	49.789	KB882236.1:427302-470940	-
TP56963	15	50.273	KB871787.1:95347-95402	22:5886315-5885924
TP73600	15	50.663	KB871629.1:478476-478413	-
TP39132	15	51.34	KB871787.1:95402-95347	22:5886315-5885924
TP10541	15	52.607	KB872355.1:83428-83365	-
TP50750	15	53.257	KB882214.1:1057425-1057488	23:10654460-10654534
TP9820	15	53.772	KB871972.1:114152-114100	-
TP44941	15	54.62	KB871724.1:595894-595957	-
TP51419	15	55.056	KB872246.1:54894-54957	-

TP43803	15	55.345	-	-
TP43601	15	55.901	KB871597.1:477178-477241	3:54148084-54148115
TP34350	15	57.467	KB882102.1:1279171-1316684	23:28737093-28811700
TP42606	15	58.325	KB872397.1:110059-110111	5:16725810-16725710
TP57284	15	59.099	KB871597.1:574429-574492	-
TP7932	15	59.605	KB882098.1:3128199-3128262	-
TP22710	15	60.332	KB871597.1:533221-533158	-
TP70424	15	61.066	KB871754.1:3126458-3126521	-
TP75613	15	62.124	KB872370.1:171449-171386	-
TP90871	15	62.652	KB871754.1:3442349-3442412	-
TP44546	15	63.998	KB882214.1:114720-114657	-
TP9472	15	65.217	KB882214.1:114624-114687	-
TP68719	15	66.675	KB871754.1:3105811-3105755	-
TP27013	15	69.061	KB871754.1:3105755-3105811	-
TP54150	15	70.492	KB871754.1:4449520-4449583	-
TP73771	15	78.424	KB872132.1:113794-129363	23:20995078-21020456
TP47506	15	80.036	KB871866.1:33383-65458	-
TP64417	15	80.654	KB882262.1:521774-521711	19:28865464-28865540
TP73562	15	81.627	KB871972.1:215328-215391	-
TP30275	15	82.859	KB871751.1:202439-202376	9:4652497-4652446
TP22044	15	83.554	KB871629.1:478362-478425	-
TP49946	15	83.682	KB871985.1:274269-291401	23:44017505-44034669
TP7249	15	84.71	KB882098.1:2270168-2270231	-
TP30163	15	85.275	KB872694.1:38995-38932	-
TP35234	15	86.662	KB882262.1:316212-316264	-
TP84638	15	87.106	KB871754.1:4236417-4245364	-
TP55497	15	87.833	KB882098.1:2270268-2270205	-
TP28515	15	88.86	KB882098.1:2709859-2770159	11:29094981-29301026
TP68036	15	89.543	KB871929.1:405844-405907	-
TP44469	15	90.891	KB872132.1:12616-12553	-
TP75346	15	93.486	KB879394.1:399-342	-

TP28623	15	94.423	KB871754.1:3866826-3866889	-
TP85846	16	0	KB882190.1:183769-183832	-
TP8329	16	0.916	KB871618.1:218734-226576	10:35982021-35985196
TP83137	16	2.048	KB882137.1:2513690-2513753	-
TP27463	16	2.811	KB871908.1:167215-167278	-
TP45554	16	4.471	KB882246.1:836076-875739	2:37792881-37818491
TP61126	16	5.581	KB871606.1:361438-361375	7:1534014-1533971
TP57293	16	6.102	KB871773.1:222877-222940	-
TP16478	16	7.449	KB872739.1:3076-29837	-
TP71407	16	7.791	KB882131.1:391258-391195	-
TP16832	16	11.83	-	-
TP70591	16	13.111	KB871748.1:736735-736798	-
TP86745	16	14.603	-	-
TP89114	16	15.297	KB871588.1:484972-485035	24:24231142-24231528
TP37352	16	16.521	KB882246.1:1124591-1124654	16:17971275-17971338
TP61496	16	17.276	-	-
TP28516	16	17.926	-	-
TP70957	16	18.802	KB872782.1:5870-5807	-
TP11926	16	19.395	-	-
TP89287	16	20.261	KB871801.1:278172-313639	-
TP36761	16	20.7	KB871801.1:278172-313639	-
TP74194	16	21.563	KB882082.1:5023944-5023905	15:43947786-43947387
TP17096	16	21.981	KB872248.1:40038-40101	18:10457421-10457458
TP66085	16	22.594	KB882246.1:1729162-1729225	-
TP48688	16	23.185	KB871606.1:1140134-1140071	-
TP61119	16	23.764	KB871801.1:489790-489727	-
TP81880	16	24.227	KB878273.1:3895-3832	25:6841867-6841837
TP7848	16	25.012	KB871801.1:29234-29171	7:60790646-60790702
TP41923	16	25.463	-	-
TP54806	16	25.7	KB871801.1:166192-166129	-
TP69770	16	26.104	KB871908.1:65847-65784	7:43505341-43505284

TP9857	16	26.53	KB871606.1:361318-361262	7:1534014-1533971
TP63308	16	27.1	KB871908.1:460-22510	-
TP71373	16	27.628	KB872248.1:67706-67643	2:31642249-31642177
TP28707	16	27.998	KB871748.1:590933-590996	-
TP57993	16	28.503	KB871801.1:509738-519583	-
TP71554	16	29.445	KB882290.1:694255-694318	-
TP69282	16	29.972	KB882290.1:715422-715485	2:30598006-30597649
TP45449	16	30.573	KB882290.1:715493-715430	2:30598006-30597649
TP34156	16	31.051	KB882290.1:79665-79728	-
TP16305	16	31.909	KB882290.1:1118686-1118623	-
TP72212	16	32.336	KB882290.1:1353204-1353267	-
TP89592	16	32.786	KB882290.1:1352823-1352886	-
TP25198	16	33.029	KB882290.1:1352925-1352862	-
TP39903	16	33.581	KB882190.1:305333-305396	-
TP44235	16	34.003	KB882290.1:1118580-1118643	-
TP14981	16	34.402	KB882290.1:1188632-1188569	23:4204534-4204691
TP70565	16	34.957	KB871658.1:171455-330615	7:59131813-59343765
TP19724	16	35.819	KB882290.1:1291221-1291158	-
TP22182	16	36.429	KB871631.1:105886-105823	-
TP77173	16	37.46	KB872027.1:265440-265503	-
TP10740	16	37.98	KB871631.1:251962-252025	-
TP89701	16	38.718	KB872027.1:265541-265478	-
TP48705	16	39.513	KB873346.1:11795-11732	18:22910186-22910290
TP44467	16	40.32	KB871631.1:941031-940968	18:30423425-30423536
TP84113	16	41.38	KB871631.1:8446-8509	22:15296743-15296770
TP9768	16	43.021	KB882205.1:271042-279222	7:19200553-19216035
TP1838	16	43.531	KB882197.1:137466-137529	-
TP10470	16	44.427	KB882197.1:151208-151271	-
TP59742	16	45.47	KB882199.1:1467619-1467682	-
TP20633	16	46.021	KB882199.1:882619-882556	7:73174630-73175408
TP46657	16	47.058	KB882114.1:3001480-3001421	25:8481272-8481337

TP78955	16	48.497	KB882114.1:1426931-1426868	-
TP81066	16	48.959	KB882114.1:2567812-2567875	-
TP10102	16	50.507	KB872818.1:3770227-3770164	-
TP10519	16	50.949	KB882114.1:46859-46796	6:24050490-24050942
TP91259	16	52.084	KB872818.1:3770071-3770134	-
TP29718	16	53.202	KB872818.1:2472125-2472063	2:13282190-13282115
TP21540	16	53.98	KB872818.1:860788-860851	-
TP79193	16	55.444	KB882131.1:2231616-2231553	-
TP1807	16	55.864	KB882131.1:2545853-2545916	-
TP48623	16	56.521	KB872818.1:75847-75784	-
TP45752	16	57.279	KB872818.1:4012-4075	-
TP44270	16	57.599	KB872818.1:4102-4039	-
TP81008	16	58.336	KB871773.1:221019-220956	-
TP20327	16	58.722	KB882131.1:2992671-2992608	-
TP40331	16	59.6	KB882131.1:1116692-1118071	-
TP43293	16	60.412	KB882190.1:305414-305351	-
TP87780	16	61.589	KB882131.1:804841-804778	-
TP68527	16	62.521	KB882131.1:1792124-1792187	-
TP27047	16	63.418	KB882131.1:2287653-2287716	-
TP33799	16	64.35	KB882131.1:2062371-2062308	-
TP16918	16	65.259	KB873811.1:12620-12564	-
TP91268	16	67.031	KB882131.1:2062258-2062321	-
TP61375	16	67.806	KB882131.1:2181151-2181088	-
TP50445	16	68.833	KB882190.1:149467-149404	-
TP80690	16	70.12	-	-
TP77677	16	71.759	KB882131.1:1796129-1796192	18:30423276-30423537
TP47075	16	72.912	-	-
TP50630	17	-9.919	KB871732.1:708582-708645	2:10321885-10321826
TP7214	17	-6.122	KB872346.1:66912-66975	-
TP54654	17	-4.044	KB871732.1:264633-264696	-
TP59876	17	-2.996	-	-

TP73551	17	-1.139	KB871660.1:79805-79742	-
TP83454	17	0	KB871856.1:21928-77032	2:54798988-54950572
TP71467	17	1.299	KB872305.1:147014-147062	6:26685-26465
TP38003	17	2.912	KB871660.1:925439-925376	6:9816328-9816398
TP45734	17	5.048	KB877523.1:825-762	-
TP71558	17	6.789	KB871620.1:860842-860789	-
TP75485	17	8.562	KB872465.1:73646-73709	-
TP25205	17	9.95	KB871688.1:473768-473821	-
TP19847	17	11.149	-	-
TP33850	17	13.555	KB882255.1:1544617-1544554	-
TP43504	17	15.075	KB871856.1:435970-436033	-
TP70832	17	16.426	KB882254.1:52809-52872	-
TP18836	17	18.409	KB871644.1:919090-919153	-
TP57842	17	20.91	KB871644.1:210556-223014	19:11478788-11505488
TP84015	17	21.755	KB882116.1:1313002-1313064	-
TP26390	17	22.473	KB872244.1:81868-81931	-
TP42219	17	23.888	KB871741.1:26891-26821	-
TP52825	17	25.263	KB882166.1:749397-749344	-
TP48158	17	26.385	KB882166.1:770956-770893	-
TP7062	17	26.994	KB882166.1:749344-749397	-
TP23615	17	27.852	-	-
TP45038	17	28.795	-	-
TP4657	17	29.507	KB882166.1:1960211-1995547	16:35319311-35372907
TP7447	17	30.32	-	-
TP60949	17	30.511	KB882166.1:1960211-1995547	16:35319311-35372907
TP91230	17	31.356	KB882166.1:1764675-1764738	-
TP82465	17	32.289	KB872819.1:464339-481953	16:30830213-30855807
TP89977	17	33.09	KB871644.1:894155-894216	-
TP60934	17	33.805	KB872000.1:219135-224937	2:42173798-42182459
TP70125	17	35.034	KB871710.1:649323-653539	11:28694916-28704282
TP47730	17	36.103	KB871701.1:381493-381556	16:6801924-6801987

TP60631	17	36.873	KB871741.1:722758-722825	-
TP13788	17	38.281	-	-
TP29812	17	38.726	-	-
TP70180	17	39.721	KB872940.1:8784-8721	25:27929209-27929178
TP16194	17	41.253	-	-
TP85518	17	42.908	KB871645.1:657334-657271	-
TP86378	17	43.688	KB871645.1:657211-657274	-
TP76078	17	45.579	KB871654.1:682885-682822	-
TP25522	17	46.598	KB871645.1:174490-174553	-
TP77410	17	47.466	KB872163.1:175474-175413	-
TP65625	17	52.908	KB871741.1:240482-308033	2:47877473-47941446
TP55767	17	55.094	KB871741.1:240482-308033	2:47877473-47941446
TP13785	17	56.122	KB871741.1:26794-26733	-
TP45625	17	57.155	KB871741.1:26726-26789	-
TP50563	17	61.219	KB871579.1:9137612-9137675	-
TP66795	17	67.15	KB871579.1:9137694-9137631	-
TP51084	18	0	KB872309.1:13455-13518	18:2757443-2759172
TP44368	18	3.043	KB882146.1:829056-829119	18:10457459-10457421
TP44387	18	5.393	KB882146.1:464037-553248	-
TP78139	18	7.976	KB882146.1:2485701-2485764	-
TP42065	18	8.751	KB882146.1:983923-983860	-
TP22439	18	10.216	KB882146.1:812713-812650	2:22238007-22237928
TP34675	18	11.108	KB882081.1:593110-593173	24:24231358-24231142
TP61830	18	12.549	KB882140.1:1534327-1534264	22:22410843-22410894
TP19821	18	13.786	KB882146.1:2489330-2540899	18:39573030-39650116
TP25058	18	15.203	KB882146.1:2947998-2947935	-
TP44577	18	15.395	KB882146.1:2947903-2947966	-
TP41288	18	16.727	KB882081.1:725620-725557	-
TP2692	18	17.229	KB882081.1:570563-570626	-
TP21179	18	17.576	KB882081.1:570650-570587	-
TP13458	18	18.68	KB882081.1:4108800-4108737	-

TP28824	18	19.825	KB882140.1:2644240-2671289	18:11506755-11566641
TP57309	18	20.119	KB882140.1:2644240-2671289	18:11506755-11566641
TP91619	18	21.34	KB882081.1:1462098-1462035	-
TP32118	18	22.623	KB882081.1:3160054-3159991	-
TP44112	18	23.406	KB882135.1:2115722-2115785	-
TP18072	18	24.086	KB882081.1:4158389-4158452	-
TP56806	18	24.566	KB882135.1:2115805-2115742	-
TP39245	18	24.912	KB882291.1:246642-246702	-
TP44730	18	25.469	KB882081.1:3686404-3723042	18:5172468-5196738
TP34535	18	26.093	KB882081.1:3084598-3084535	11:42616435-42616405
TP69268	18	26.372	KB882081.1:3024335-3029242	-
TP79905	18	26.759	KB882081.1:4543525-4543588	-
TP6894	18	27.724	KB882081.1:3265260-3265323	18:30423499-30423341
TP73352	18	28.338	-	-
TP8477	18	28.99	KB882135.1:1301761-1301698	-
TP51023	18	29.988	KB882140.1:755172-788683	-
TP86616	18	30.768	KB882135.1:2291929-2291866	-
TP42817	18	31.589	KB882140.1:1471844-1471907	-
TP80560	18	32.383	KB882135.1:2270414-2270477	-
TP91327	18	33.145	KB882135.1:2614307-2638535	18:15520525-15550461
TP65437	18	33.583	KB882123.1:3022205-3022268	-
TP14659	18	34.091	KB882123.1:3053358-3065948	-
TP21064	18	34.633	KB882135.1:2789148-2797490	-
TP26941	18	35.152	KB882135.1:539163-539100	-
TP31977	18	35.758	KB882135.1:431684-431747	-
TP2621	18	36.15	KB882123.1:2433179-2433116	-
TP13084	18	36.758	KB882123.1:3022285-3022222	-
TP87746	18	37.208	KB882135.1:1301635-1301698	-
TP59645	18	38.164	KB882135.1:2614307-2638535	18:15520525-15550461
TP15608	18	38.476	KB882123.1:1848428-1874378	-
TP36954	18	39.054	KB882123.1:1848428-1874378	-

TP5018	18	39.633	KB882123.1:778717-778654	-
TP42693	18	40.03	KB882209.1:78608-78545	-
TP59827	18	40.58	KB882123.1:13741-13804	18:19911704-19911311
TP89904	18	41.046	KB882212.1:1489415-1489352	1:15015467-15015505
TP36365	18	41.73	-	-
TP37471	18	42.399	KB871764.1:471035-471074	12:8857982-8858175
TP30729	18	43.544	KB882212.1:1489342-1489405	1:15015467-15015505
TP63069	18	44.079	-	-
TP16385	18	44.642	KB882212.1:737264-737201	-
TP37640	18	45.405	KB871864.1:153909-288728	18:26402611-26454918
TP55317	18	46.185	KB882187.1:1910906-1910969	24:24231192-24231632
TP78721	18	47.117	KB882274.1:1680982-1681045	-
TP74546	18	47.559	KB871864.1:409591-409654	-
TP82445	18	48.853	KB882187.1:1910980-1910917	24:24231192-24231632
TP19924	18	49.395	KB882274.1:37794-37857	-
TP44726	18	50.031	KB882274.1:1159148-1159085	-
TP73569	18	51.596	-	-
TP40327	18	52.151	KB871693.1:125994-126057	-
TP60935	18	53.577	KB871679.1:924093-942739	-
TP59798	18	54.14	KB882149.1:29187-29250	-
TP19233	18	54.803	KB871693.1:798499-798436	24:8762079-8762180
TP22913	18	55.288	KB882149.1:313281-313344	-
TP77441	18	55.577	-	-
TP13647	18	56.466	KB871693.1:722935-917248	18:39654310-39805917
TP38972	18	56.569	KB871693.1:798427-798490	24:8762079-8762180
TP34077	18	57.972	KB882187.1:1987043-1987106	-
TP57372	18	59.102	KB871679.1:560978-561041	-
TP11670	18	59.741	-	-
TP19435	18	61.61	KB882149.1:2317510-2317447	-
TP77163	18	62.676	KB882171.1:3979266-3979319	21:27132568-27132596
TP14765	18	63.663	KB882149.1:2515644-2515707	-

TP70949	18	64.899	KB873582.1:9904-9851	-
TP89629	18	65.071	KB872096.1:359967-360030	-
TP82117	18	66.11	-	-
TP23953	18	68.535	KB871797.1:353106-414705	20:20037083-20255031
TP72449	18	70.096	KB871808.1:15698-15635	-
TP53189	18	71.26	-	-
TP30563	18	72.356	KB882110.1:2804662-2804604	-
TP85255	18	74.711	-	-
TP46925	18	75.92	-	-
TP91160	18	77.011	-	-
TP90961	18	78.699	-	-
TP20834	18	80.618	-	-
TP69242	18	83.494	KB872211.1:53802-53753	-
TP76212	18	85.488	KB872777.1:12979-12916	-
TP38393	18	89.466	KB874764.1:584-647	-
TP27112	19	0	KB871772.1:316402-316465	-
TP27565	19	3.379	KB871578.1:5556203-5593339	9:35404357-35454002
TP28853	19	6.767	KB871801.1:332817-332871	-
TP39625	19	9.694	KB871827.1:631159-631222	-
TP18844	19	11.34	-	-
TP40062	19	14.104	KB882170.1:2526946-2526883	3:32183149-32183263
TP43300	19	15.303	KB871827.1:656806-656743	-
TP80349	19	17.813	KB882170.1:1266555-1266492	22:6430314-6430190
TP31723	19	19.106	KB872067.1:29118-56328	-
TP19829	19	21.082	KB882170.1:967660-967723	-
TP52822	19	23.536	KB882178.1:232237-232300	18:30423343-30423533
TP50938	19	24.405	-	-
TP20424	19	25.821	-	-
TP85170	19	26.489	KB872167.1:151170-151107	-
TP86913	19	27.799	KB882215.1:1014457-1014394	-
TP87779	19	29.068	KB882215.1:796049-796112	-

TP72698	19	30.233	KB882215.1:1095489-1095426	-
TP75336	19	31.027	KB882312.1:988402-988339	-
TP33210	19	31.846	KB872197.1:181828-181891	16:34107963-34107864
TP42994	19	32.954	KB872280.1:27807-27744	-
TP40401	19	33.286	KB882312.1:952751-952688	-
TP70251	19	33.967	KB871791.1:11984-11921	-
TP9385	19	35.263	KB882312.1:952681-952744	-
TP60097	19	35.613	KB871791.1:138062-137999	-
TP56236	19	36.447	KB882244.1:926329-926392	20:39721892-39721929
TP74061	19	36.831	KB882244.1:638387-650902	-
TP4997	19	38.257	KB882244.1:926425-926362	20:39721892-39721929
TP63064	19	38.864	KB882244.1:638387-650902	-
TP5687	19	40.065	KB882244.1:647219-647282	-
TP78559	19	40.996	KB882312.1:97678-97741	-
TP30944	19	41.753	KB871829.1:179373-179436	-
TP79850	19	42.702	KB882173.1:279435-279372	-
TP63843	19	43.48	KB871712.1:732060-732123	-
TP10596	19	43.998	KB882173.1:279363-279426	-
TP17862	19	44.82	KB882147.1:2378637-2404717	9:19657654-19676003
TP87661	19	45.371	KB882147.1:2378637-2404717	9:19657654-19676003
TP86998	19	46.818	KB882147.1:354040-353977	-
TP13030	19	48.299	KB872089.1:79975-80038	9:25467446-25467286
TP75772	19	48.968	KB871877.1:61054-60991	-
TP17805	19	49.905	KB871877.1:30644-30707	-
TP75034	19	50.779	KB882203.1:82565-82628	3:44611778-44611914
TP9067	19	51.593	KB871877.1:60990-61053	-
TP10606	19	52.503	KB871877.1:241894-241831	-
TP56052	19	52.839	KB882082.1:7479124-7479187	-
TP75399	19	53.874	KB872163.1:230167-230104	-
TP18082	19	54.849	KB882136.1:1323649-1323586	-
TP1141	19	55.314	KB882136.1:1328276-1414333	9:26958526-27023334

TP48485	19	56.02	KB882300.1:712374-712437	-
TP4413	19	56.621	KB882082.1:7337321-7337384	Zv9_NA797:110219-110192
TP64373	19	56.716	KB882244.1:719966-720029	21:44482645-44482692
TP24608	19	57.731	KB882300.1:1179375-1179312	-
TP7952	19	58.884	KB882300.1:1258875-1258938	-
TP56553	19	59.763	KB882296.1:677617-677554	-
TP61641	19	60.613	KB882296.1:785193-785256	-
TP57730	19	61.246	KB882296.1:677492-677555	-
TP68650	19	62.214	KB882082.1:8869106-8869043	-
TP87966	19	62.353	KB871578.1:8217774-8217837	9:38757817-38757685
TP26027	19	62.89	KB882215.1:1550279-1550216	-
TP69862	19	63.361	KB871578.1:8217847-8217784	9:38757817-38757685
TP74161	19	63.813	KB871578.1:9659332-9659395	5:28767791-28767837
TP56271	19	65.667	KB882203.1:1616170-1616107	1:30453639-30453733
TP31999	19	66.096	KB882168.1:1696113-1740639	1:31396933-31433482
TP29882	19	66.256	KB882082.1:8869015-8869078	-
TP60743	19	66.775	KB871578.1:7804067-7804004	-
TP26379	19	67.633	-	-
TP38574	19	68.019	KB871578.1:4388104-4388042	-
TP66676	19	68.652	KB882082.1:7684419-7684356	-
TP9854	19	69.443	KB871578.1:4119893-4119956	9:32014496-32014172
TP57426	19	71.399	KB871578.1:4388042-4388104	-
TP74810	19	71.831	KB871578.1:5076671-5076608	-
TP40933	19	72.58	KB871578.1:7651442-7726800	9:38194145-38349217
TP17146	19	73.581	KB871578.1:7470334-7470271	-
TP68555	19	74.549	KB882082.1:8789927-8789864	2:7913236-7913361
TP45065	19	77.354	-	-
TP87464	19	78.861	KB882173.1:2308481-2308544	-
TP47748	19	80.012	KB882244.1:599416-599353	1:24078188-24078218
TP76967	19	81.556	-	-
TP73070	19	81.839	KB882170.1:391116-391179	-

TP42078	19	82.568	-	-
TP81921	19	83.731	KB882136.1:2951257-2951194	-
TP77058	19	84.469	KB871626.1:781237-781174	-
TP80811	19	84.766	KB882296.1:826409-826472	-
TP66826	19	86.169	KB882300.1:637406-637469	9:30036396-30036565
TP71669	19	87.522	KB882136.1:299148-299085	-
TP25757	19	88.603	KB871578.1:5263307-5263244	-
TP76734	19	94.298	KB882203.1:1465363-1495711	-
TP24998	20	0	-	-
TP52343	20	3.211	KB882132.1:2691259-2691196	-
TP2595	20	7.358	KB872983.1:11808-14876	-
TP14093	20	9.462	-	-
TP56276	20	15.332	KB882132.1:1032249-1032312	17:35849478-35849430
TP7313	20	20.804	KB882132.1:1041007-1040944	-
TP11400	20	22.632	KB882132.1:1073974-1073911	19:10272159-10272127
TP50936	20	25.195	KB871675.1:124452-163843	-
TP84325	20	27.749	KB882085.1:3851205-3851142	19:35003609-35003664
TP9769	20	29.634	KB871675.1:124452-163843	-
TP41400	20	31.626	KB882085.1:3700603-3714264	10:1623969-1663150
TP47503	20	32.368	KB882085.1:4349605-4349668	-
TP56312	20	36.578	KB882085.1:2803103-2803152	19:40064423-40064387
TP57779	20	37.16	KB882085.1:2803152-2803103	19:40064423-40064387
TP92028	20	39.28	KB882085.1:1172790-1172727	-
TP34980	20	39.709	KB882085.1:2646506-2724801	10:38602081-38728184
TP33148	20	40.017	KB882085.1:1976126-1976063	-
TP67270	20	40.257	KB871622.1:1261773-1264122	-
TP65935	20	41.491	KB871849.1:68755-68692	-
TP39513	20	42.3	KB871901.1:171777-171714	4:23917467-23917407
TP70433	20	43.143	KB871622.1:1261773-1264122	-
TP80466	20	43.719	KB871901.1:171710-171773	4:23917467-23917407
TP6499	20	44.081	KB872500.1:23110-23047	16:51857437-51857822

TP88659	20	44.873	KB872117.1:122661-122598	17:6129397-6128567
TP54968	20	45.71	KB871901.1:71019-71082	-
TP88658	20	46.375	KB872282.1:70567-70504	-
TP29022	20	47.238	KB871901.1:289584-289647	25:29483402-29483429
TP3789	20	47.729	-	-
TP88624	20	48.303	KB871675.1:745423-745486	25:37483987-37483960
TP1112	20	48.901	KB882198.1:1472153-1514619	12:12186820-12263281
TP37641	20	49.363	-	-
TP44063	20	49.767	KB872282.1:69375-69438	-
TP1104	20	50.018	KB872126.1:6543-6606	-
TP20612	20	50.853	-	-
TP13392	20	51.3	KB882272.1:459278-459341	-
TP62573	20	51.841	KB872953.1:10276-24702	15:39974169-39983303
TP34399	20	52.273	KB871675.1:745506-745443	25:37483987-37483960
TP20905	20	52.673	-	-
TP64276	20	53.219	KB882272.1:371154-371091	-
TP56967	20	53.932	KB871901.1:19609-19546	-
TP74128	20	54.382	KB871901.1:289704-289641	25:29483402-29483429
TP65933	20	54.603	KB871849.1:69165-69102	-
TP2575	20	54.92	KB871849.1:69347-69410	-
TP65930	20	55.368	KB871849.1:69422-69359	-
TP45759	20	55.778	KB882272.1:397769-417114	10:34257606-34370562
TP43845	20	56.419	KB882272.1:425658-453469	-
TP64062	20	57.286	KB871849.1:273398-273335	-
TP70892	20	58.368	KB872004.1:379049-379112	-
TP39536	20	59.099	KB871689.1:200221-200284	17:44053619-44053380
TP87004	20	59.562	KB882241.1:227194-257917	10:17745086-17758385
TP67996	20	60.099	KB871593.1:220983-220920	-
TP84949	20	60.417	KB871593.1:220918-220981	-
TP70198	20	61.272	KB871689.1:567355-567292	-
TP43048	20	61.792	KB882241.1:91636-91573	-

TP88932	20	62.623	KB882196.1:182042-225692	10:20214096-20268316
TP85954	20	62.773	KB882196.1:182042-225692	10:20214096-20268316
TP84061	20	63.503	KB882196.1:339522-339459	-
TP3926	20	63.787	KB882241.1:91558-91621	-
TP3960	20	64.689	KB871593.1:290700-300617	10:6929512-6938339
TP2716	20	65.477	KB882196.1:40033-39970	7:12636183-12636154
TP41616	20	66.325	KB871689.1:130276-130213	-
TP77931	20	67.094	KB882183.1:1946671-1946608	4:8296290-8296046
TP45103	20	68.251	KB882183.1:1685187-1740317	10:4511584-4547733
TP3617	20	69.121	KB882201.1:793579-793642	-
TP83349	20	69.635	KB882201.1:793672-793609	-
TP91740	20	70.382	KB882183.1:1946516-1946579	4:8296290-8296046
TP9391	20	71.067	KB882157.1:668312-668375	-
TP76924	20	71.86	KB871752.1:431532-431469	17:23621566-23621538
TP74480	20	72.267	KB871752.1:431459-431522	17:23621566-23621538
TP81593	20	72.959	KB882201.1:407909-407846	-
TP83295	20	73.486	KB882201.1:91872-91935	-
TP66874	20	74.221	KB882201.1:501876-501813	-
TP5171	20	75.506	KB882201.1:592172-592235	-
TP73953	20	75.985	KB882157.1:668431-668368	-
TP57468	20	76.434	KB882157.1:1794260-1794197	8:2482544-2482024
TP55631	20	77.514	KB882157.1:1655721-1655784	-
TP26043	20	78.253	KB882201.1:261610-261673	25:29483402-29483445
TP16110	20	79.245	KB882157.1:1749092-1749029	-
TP55028	20	79.978	KB882201.1:261739-261676	25:29483402-29483445
TP56249	20	81.001	KB882157.1:288212-288161	-
TP54863	20	81.897	KB882157.1:867499-867436	-
TP68667	20	83.94	KB882248.1:1399379-1399316	-
TP276	20	85.085	KB871899.1:45558-45495	-
TP80370	20	87.065	KB871899.1:45482-45545	-
TP91090	20	88.647	KB871899.1:210848-210911	-

TP75198	20	91.1	KB882248.1:344307-344370	-
TP58712	20	93.501	-	-
TP25178	20	96.382	KB871661.1:697877-697831	-
TP14365	21	0	KB871605.1:919995-920058	1:13068753-13068632
TP69011	21	3.719	KB871605.1:920076-920013	1:13068753-13068632
TP68576	21	8.553	KB871605.1:476113-476050	1:40545514-40545585
TP19642	21	12.367	KB871605.1:476001-475938	1:40545514-40545585
TP57435	21	15.403	KB871605.1:377129-377192	-
TP10002	21	19.412	-	-
TP59170	21	21.503	-	-
TP35054	21	25.375	KB871605.1:476186-476123	1:40545514-40545585
TP75776	21	29.114	KB871763.1:301412-301347	-
TP43675	21	30.37	-	-
TP48768	21	32.107	-	-
TP24383	21	33.666	-	-
TP9077	21	35.426	KB871763.1:301347-301412	-
TP16139	21	36.935	-	-
TP21649	21	39.467	-	-
TP32025	21	42.102	KB873883.1:2469-2532	8:47642662-47642759
TP57547	21	42.98	KB873019.1:12158-20406	-
TP27826	21	44.106	-	-
TP3005	21	45.203	-	-
TP40400	21	46.797	KB873883.1:2606-2543	8:47642662-47642759
TP46518	21	49.872	KB871630.1:351554-351491	-
TP4784	21	51.246	KB882094.1:495560-515720	8:20993152-21009564
TP67962	21	52.463	KB882090.1:3577937-3617426	20:37325307-37401268
TP40794	21	53.44	KB871630.1:749959-749896	-
TP83556	21	55.154	KB871630.1:2732780-2732717	-
TP15349	21	56.251	KB871630.1:170232-186812	-
TP37042	21	58.199	KB871630.1:2425077-2451119	13:328952-350855
TP78747	21	59.955	KB871630.1:4381274-4381337	-

TP2492	21	60.296	KB871992.1:388691-393208	-
TP32204	21	63.213	KB871630.1:4058370-4058307	-
TP81952	21	67.679	KB871630.1:5000240-5000177	-
TP76818	21	70.014	KB871630.1:5365036-5364973	1:17861592-17861470
TP91388	21	71.693	KB871731.1:120401-120464	-
TP80718	21	73.877	KB871630.1:5516431-5533521	-
TP52371	21	75.754	KB871630.1:5516431-5533521	-
TP38899	21	80.064	-	-
TP75944	21	82.882	KB871667.1:45006-45062	-
TP59346	21	84.521	KB871667.1:45062-45006	-
TP42194	21	87.656	KB882169.1:1988979-1989042	11:753377-753350
TP87674	21	90.134	KB882169.1:1989056-1988993	11:753377-753350
TP88257	21	93.339	KB882169.1:1382398-1382335	18:19099433-19099150
TP75712	21	94.961	KB882169.1:292097-292035	-
TP29000	21	96.726	KB882169.1:1336453-1336390	-
TP64227	21	98.7	KB882169.1:529273-548228	18:15362233-15372316
TP19506	21	100.154	KB882169.1:760025-808887	11:44132223-44140551
TP47954	21	101.939	KB882169.1:796611-796548	11:44139614-44139999
TP52735	21	104.43	KB882169.1:760025-808887	11:44132223-44140551
TP53719	21	106.703	KB882169.1:962646-974358	13:354637-362295
TP43124	21	111.963	-	13:432857-432794
TP4910	21	114.508	KB872242.1:78288-78351	-
TP12873	22	0	KB882145.1:6512380-6512317	-
TP74403	22	3.261	KB871987.1:309719-309782	8:9120145-9120363
TP45732	22	5.894	KB882145.1:7257210-7257147	-
TP3822	22	7.368	KB882145.1:7399137-7399200	17:20818877-20818843
TP72334	22	8.785	KB882145.1:6770048-6770111	-
TP81342	22	10.762	KB882145.1:6512280-6512343	-
TP23411	22	12.762	-	-
TP14547	22	14.736	KB882145.1:6770115-6770052	-
TP91147	22	15.673	KB877205.1:1458-1521	-

TP35104	22	17.142	KB880823.1:885-824	22:24745761-24745709
TP65186	22	17.986	KB880823.1:824-885	22:24745761-24745709
TP13511	22	19.563	KB877205.1:1580-1517	-
TP25073	22	21.65	KB882145.1:4547796-4547733	-
TP61698	22	22.404	KB882145.1:4422197-4422260	-
TP44523	22	24.188	KB882145.1:4547697-4547760	-
TP25924	22	25.106	KB882145.1:4621488-4637341	11:43680161-43707068
TP83443	22	26.14	KB882145.1:3064887-3064824	17:30836612-30836644
TP83698	22	26.385	KB882145.1:3456590-3456653	-
TP60989	22	28.346	KB882145.1:3064799-3064862	17:30836612-30836644
TP63512	22	29.372	KB882111.1:1996562-1996625	11:22649144-22649324
TP52895	22	30.096	KB882145.1:2926914-2952626	2:48424681-48470959
TP84206	22	30.79	KB882145.1:2732012-2731949	11:41011830-41011978
TP20433	22	31.011	KB882145.1:2636546-2896506	11:41011852-41403023
TP33983	22	31.943	KB882145.1:1197898-1197835	-
TP79121	22	32.874	KB882116.1:2991466-2991403	-
TP433	22	33.676	KB871822.1:381371-381434	16:45936008-45936035
TP11365	22	34.667	KB871590.1:982424-982361	22:24745709-24745761
TP4765	22	35.128	KB871590.1:982325-982388	22:24745709-24745761
TP14419	22	35.706	KB882145.1:1197809-1197872	-
TP55846	22	36.471	KB872005.1:139312-139375	-
TP78202	22	36.939	KB872230.1:42570-42507	17:25710558-25710525
TP25060	22	37.403	KB882134.1:388487-415526	5:42724184-42742823
TP72510	22	38.099	KB872005.1:84224-84287	6:10138974-10139009
TP18243	22	38.713	KB882218.1:340797-340734	8:45270996-45270929
TP30896	22	39.564	-	-
TP87880	22	39.707	KB882116.1:2261597-2274793	-
TP46254	22	40.441	KB882218.1:1707150-1707087	-
TP26657	22	41.184	KB882116.1:3239756-3239693	-
TP35408	22	41.642	KB882116.1:2148032-2147974	-
TP8346	22	42.395	KB871614.1:400341-400278	9:52712011-52711984

TP56838	22	42.822	KB871581.1:191849-191786	-
TP58085	22	43.494	KB882116.1:329088-329151	-
TP44531	22	43.787	KB871641.1:835520-835583	-
TP7906	22	44.244	KB871804.1:245030-245093	-
TP68286	22	44.843	KB882169.1:915566-921930	13:406179-417379
TP46342	22	45.487	KB882116.1:793429-793366	-
TP73644	22	45.782	KB871869.1:133672-154722	23:32277965-32299462
TP61724	22	46.14	KB871614.1:206154-206217	-
TP65081	22	46.608	KB882116.1:2261817-2261880	-
TP52919	22	47.099	KB882116.1:2147974-2148032	-
TP17784	22	47.683	KB882225.1:578053-578116	-
TP55032	22	48.699	KB882145.1:4319731-4319668	-
TP36307	22	48.995	KB882116.1:830386-830323	-
TP26731	22	49.834	KB882116.1:2332706-2332643	9:4652489-4652462
TP17163	22	50.46	KB871584.1:687098-687161	8:6036044-6036301
TP23983	22	50.841	KB882111.1:1726771-1726708	-
TP29799	22	51.336	KB882116.1:2261597-2274793	-
TP83857	22	51.692	KB882218.1:340722-340785	8:45270996-45270929
TP17493	22	53.09	KB871584.1:470175-470112	-
TP83185	22	53.614	KB882111.1:252079-263163	11:18496932-18517447
TP60439	22	54.546	KB871804.1:301908-318602	-
TP35055	22	55.281	KB882189.1:117401-123117	3:41950140-42081359
TP16450	22	55.924	KB871804.1:373721-373658	-
TP6850	22	56.833	KB872230.1:150780-150843	-
TP28750	22	57.912	KB872230.1:150856-150793	-
TP83366	22	58.435	KB882111.1:1530595-1530658	16:58694577-58694632
TP42190	22	59.857	KB882225.1:503729-503792	-
TP42318	22	60.732	KB871804.1:11996-11933	23:34670652-34670438
TP19418	22	62.354	KB882189.1:2200192-2205339	-
TP8041	22	64.05	KB871804.1:325025-339462	22:10725222-10749168
TP17427	22	66.756	KB871822.1:85987-86050	-

TP77145	22	67.793	KB871822.1:540072-558383	11:25662905-25690375
TP50301	22	68.436	KB882116.1:527809-527746	-
TP75829	22	69.181	KB882116.1:567004-567067	-
TP44397	22	69.622	KB871641.1:646049-645986	-
TP57163	22	70.494	KB871581.1:193879-193928	11:30278368-30278447
TP66427	22	71.017	KB871584.1:866386-989189	11:24277502-24393171
TP27953	22	71.455	KB871822.1:540072-558383	11:25662905-25690375
TP36129	22	71.74	KB871614.1:1074425-1091771	-
TP56148	22	72.429	KB871581.1:193928-193879	11:30278368-30278447
TP62841	22	73.208	KB882145.1:935806-935869	-
TP74593	22	74.229	KB871614.1:760470-760533	11:20292486-20292452
TP44061	22	74.921	KB882116.1:2021589-2021526	-
TP66518	22	75.497	KB882116.1:3464065-3496613	11:25754101-25785015
TP80179	22	76.374	KB882116.1:1767759-1767822	23:22945728-22945765
TP71512	22	77.009	KB876853.1:1126-1189	-
TP36926	22	77.356	KB882111.1:3226036-3226099	2:28951788-28951584
TP19488	22	77.909	KB882116.1:1096821-1096758	8:53716452-53716556
TP24360	22	78.389	KB882145.1:1164453-1164516	-
TP85396	22	79.655	KB882116.1:1096746-1096809	8:53716452-53716556
TP91563	22	80.527	KB871822.1:230507-230570	-
TP24888	22	81.857	KB871590.1:188828-222184	11:20148048-20195332
TP33309	22	82.192	KB882111.1:1430242-1430179	-
TP64021	22	83.163	KB871590.1:519409-589490	11:18817201-18854363
TP39631	22	84.161	KB871590.1:188828-222184	11:20148048-20195332
TP66513	22	84.831	KB871590.1:163722-163785	6:12336957-12336984
TP31344	22	86.241	KB882111.1:1641925-1641862	-
TP37192	22	86.763	KB882218.1:1415565-1415502	-
TP64471	22	88.169	KB882189.1:1416815-1416752	22:24745709-24745766
TP86604	22	91.083	KB882189.1:2300254-2300317	-
TP29351	23	0	KB882229.1:296866-296908	3:25168269-25168615
TP26695	23	3.356	KB872225.1:135613-135670	-

TP56371	23	4.483	KB872142.1:61433-69223	3:2139248-2139927
TP42112	23	6.888	-	-
TP81674	23	8.241	KB871897.1:261023-261086	3:1947810-1947723
TP67292	23	8.991	KB871762.1:580574-580637	8:49765598-49765554
TP50112	23	10.968	KB871897.1:491615-491678	-
TP23368	23	11.856	KB871762.1:35629-35566	22:41380994-41381336
TP38583	23	13.945	KB882167.1:68851-86940	16:36464082-36473792
TP63343	23	14.737	KB882167.1:68851-86940	16:36464082-36473792
TP57340	23	15.857	KB882167.1:1017638-1017701	16:27227592-27227471
TP39791	23	16.834	KB871897.1:249132-265016	3:1940165-1953609
TP48308	23	17.796	KB882167.1:1343226-1343163	-
TP76993	23	18.928	KB882167.1:1845465-1845528	-
TP70466	23	19.859	KB882167.1:1060613-1060550	-
TP7045	23	21.185	KB871640.1:826658-846249	-
TP77064	23	23.51	KB871640.1:169419-180241	19:9205454-9222922
TP46690	23	24.737	KB871640.1:51303-51240	-
TP3624	23	25.665	KB871640.1:51195-51258	-
TP75035	23	26.764	KB871640.1:521741-521804	-
TP41572	23	27.901	KB871640.1:111876-111939	-
TP21525	23	29.13	KB871927.1:234658-234721	-
TP32348	23	30.835	KB882192.1:54650-54587	-
TP88189	23	31.46	KB872563.1:65737-65787	-
TP21416	23	32.192	KB871649.1:2610-2547	-
TP26890	23	33.807	KB871921.1:150608-150671	19:5783059-5782706
TP7942	23	34.872	KB871649.1:492790-492727	-
TP38204	23	36.194	KB871649.1:695219-715321	19:12520943-12573746
TP38547	23	36.943	KB871649.1:160456-180803	19:49796266-49809511
TP42010	23	37.813	KB871582.1:589169-589232	-
TP47434	23	38.733	KB871582.1:606994-641130	19:26826401-26864320
TP64325	23	40.01	KB871686.1:690813-690750	-
TP51335	23	40.877	KB871686.1:614461-614398	-

TP13710	23	41.845	KB871686.1:739471-739408	-
TP72332	23	42.73	KB882124.1:7517480-7517543	24:35555112-35555140
TP88167	23	44.968	KB882124.1:6682124-6682187	-
TP81353	23	46.018	KB882124.1:3490222-3490159	-
TP44468	23	47.184	KB882124.1:4944007-4944070	19:39456832-39456364
TP65403	23	48.081	KB882124.1:3164090-3164027	-
TP56510	23	49.072	KB882124.1:4730900-4730837	-
TP31735	23	50.26	KB882124.1:1456517-1456580	19:33201643-33201760
TP45060	23	51.312	KB882124.1:4730832-4730895	-
TP84731	23	52.157	KB871638.1:220595-220532	-
TP52209	23	53.065	KB882124.1:862140-879687	19:32255095-32280467
TP43492	23	54.151	KB882124.1:1997924-1997987	-
TP78398	23	55.693	KB882124.1:1456593-1456530	19:33201643-33201760
TP72599	23	56.946	KB871638.1:298959-298896	-
TP30814	23	57.887	KB871638.1:202489-202426	19:37607288-37607457
TP66102	23	59.439	KB882263.1:1336538-1336601	-
TP14048	23	60.291	KB871638.1:134325-134262	-
TP85475	23	60.807	KB882263.1:458481-587262	19:36588322-36874120
TP23080	23	61.937	KB877690.1:1240-3211	3:41950140-42081359
TP13018	23	62.785	KB882263.1:458481-587262	19:36588322-36874120
TP77126	23	63.539	KB882282.1:560674-585497	19:41822479-41859226
TP85458	23	64.666	KB882282.1:560674-585497	19:41822479-41859226
TP65048	23	65.314	KB882093.1:248945-248882	-
TP77091	23	66.129	KB882093.1:1361897-1361960	-
TP79387	23	66.759	KB882093.1:1394554-1394491	-
TP9161	23	67.425	KB882093.1:953553-953490	-
TP46172	23	67.987	KB882093.1:1341120-1366066	-
TP14616	23	68.775	KB882093.1:966722-983279	19:44657609-44692582
TP74021	23	69.562	KB882093.1:2672538-2672601	22:24745723-24745766
TP63951	23	69.981	KB882093.1:3257110-3257173	-
TP57737	23	71.274	KB882093.1:3668172-3772685	-

TP38362	23	71.636	KB882093.1:3668172-3772685	-
TP4658	23	72.721	KB882282.1:1445003-1444940	-
TP46931	23	73.14	KB882093.1:588929-588866	16:45936056-45936008
TP74882	23	73.984	KB882093.1:1874186-1874123	-
TP63572	23	74.886	KB882093.1:3920077-3920140	8:39234663-39234709
TP26642	23	75.555	KB882093.1:2160725-2160662	-
TP27255	23	77.466	KB882112.1:3335523-3342185	-
TP77602	23	78.678	KB882112.1:2253197-2253134	-
TP82461	23	79.039	KB871948.1:15580-15639	-
TP18421	23	80.158	KB882112.1:2404616-2404553	-
TP80882	23	82.284	KB882112.1:1113111-1113048	-
TP72788	23	84.103	KB882279.1:632896-683561	3:43657886-43692220
TP37163	23	85.673	KB882112.1:794506-794443	17:18307978-18308025
TP15246	23	87.175	KB882279.1:180026-179963	16:56735781-56735893
TP4846	23	88.581	KB871692.1:436644-436707	3:43147901-43147595
TP69718	23	90.569	KB871692.1:7826-7889	10:14403904-14403806
TP34489	23	94.27	KB871692.1:436761-436698	3:43147901-43147551
TP29956	24	-2.091	KB871902.1:9742-9805	-
TP38607	24	-0.601	KB871808.1:8635-8572	-
TP79274	24	0	KB882099.1:1603214-1603151	-
TP44163	24	0.789	KB872032.1:277047-305299	2:26838033-26868941
TP86799	24	2.163	KB871808.1:8509-8572	-
TP1601	24	2.973	KB882108.1:1309237-1309174	-
TP79136	24	4.097	KB882108.1:1318525-1318462	-
TP67547	24	4.812	KB871793.1:193709-207530	14:20023591-20053282
TP85511	24	5.333	-	-
TP57010	24	6.251	KB871580.1:2223111-2240218	25:17238046-17298675
TP13557	24	6.931	KB872572.1:25016-25079	-
TP7949	24	7.699	KB871580.1:2533139-2533202	-
TP39801	24	8.614	KB872572.1:20205-20142	11:14057673-14057717
TP88468	24	9.822	KB871759.1:222098-222040	14:28923944-28924316

TP15094	24	17.926	KB871580.1:2533318-2533255	-
TP57835	24	18.805	KB871580.1:2533253-2533316	-
TP38490	24	18.94	KB882119.1:3429666-3429729	-
TP4089	24	20.984	KB871580.1:1411223-1411160	18:30423487-30423454
TP76879	24	21.125	KB872512.1:73595-73658	-
TP58462	24	22.883	KB871580.1:1760798-1760735	-
TP16229	24	24.313	KB871580.1:643870-676224	25:15130720-15159786
TP9247	24	24.847	KB871596.1:513081-603802	2:3178319-3182992
TP25206	24	25.782	KB871941.1:435003-435066	-
TP34359	24	25.96	KB871941.1:356964-356901	-
TP72784	24	26.048	-	-
TP79782	24	26.954	KB882083.1:2660641-2660604	11:41579690-41579049
TP3984	24	27.962	-	-
TP66844	24	29.024	KB872774.1:6565-6502	11:20292486-20292452
TP20734	24	29.727	-	-
TP73152	24	29.816	KB871711.1:692270-692333	-
TP83862	24	30.264	KB872910.1:20659-20596	-
TP1095	24	30.785	-	-
TP54998	24	31.781	KB871786.1:328738-328675	-
TP64816	24	32.259	KB872419.1:68144-68207	-
TP26810	24	32.369	KB871711.1:692362-692299	-
TP69288	24	32.898	-	-
TP53980	24	33.306	KB873051.1:4815-21252	4:59499479-59598537
TP46684	24	34.167	-	-
TP13262	24	34.798	KB872910.1:19390-19327	25:13665233-13665358
TP76055	24	35.423	KB871793.1:628369-628432	7:60995467-60995344
TP3459	24	35.802	KB871711.1:81680-81623	-
TP83926	24	35.817	-	-
TP47893	24	36.43	KB882116.1:185726-185668	-
TP62797	24	36.906	KB871793.1:336007-336073	-
TP64208	24	37.717	-	-

TP28306	24	37.892	KB872442.1:6745-12451	-
TP85613	24	38.15	-	-
TP78901	24	38.658	-	-
TP47573	24	39.017	KB871711.1:81623-81680	-
TP58083	24	39.577	-	-
TP70406	24	40.332	-	-
TP32558	24	40.943	KB872442.1:90783-90720	-
TP32149	24	41.729	KB872442.1:2415-2478	-
TP31311	24	41.955	KB882119.1:3530439-3530502	-
TP17822	24	42.346	KB872065.1:63839-63776	-
TP27478	24	42.928	KB872489.1:55398-55461	-
TP31743	24	43.516	-	-
TP3018	24	44.183	KB872458.1:77458-77521	-
TP63839	24	44.922	-	-
TP4418	24	45.176	KB871841.1:342588-352853	14:22784667-22819971
TP56708	24	46.037	KB872123.1:164135-164072	-
TP17616	24	46.393	KB871841.1:411794-434151	14:22624150-22661599
TP91869	24	46.776	-	-
TP58297	24	47.743	KB871621.1:962864-962801	-
TP58064	24	48.513	KB871708.1:372971-373034	-
TP76707	24	49.195	KB871621.1:881643-881703	-
TP91624	24	49.253	-	-
TP10115	24	49.932	KB871621.1:601263-601200	-
TP34835	24	50.323	KB871621.1:300026-300089	-
TP64336	24	50.825	KB882091.1:2657646-2657583	8:53716768-53716452
TP43930	24	51.854	KB882091.1:2854972-2854909	6:9816330-9816358
TP27017	24	53.295	KB882091.1:1042429-1042492	18:10457431-10457459
TP62482	24	53.329	KB882091.1:986513-986450	-
TP67872	24	54.536	KB882260.1:1135008-1134945	14:32008039-32008325
TP32878	24	54.996	KB882164.1:2304560-2304497	Zv9_scaffold3495:121328-121660
TP34185	24	55.61	KB882186.1:843774-868333	14:33773571-33801002

TP60297	24	56.146	KB882108.1:1021757-1021818	-
TP8813	24	56.287	KB882099.1:1083724-1116040	14:25848223-25872579
TP67734	24	56.731	KB882186.1:339714-339777	15:7679791-7679763
TP64176	24	57.735	KB882119.1:2683309-2683246	-
TP6801	24	58.475	KB871979.1:74789-150567	8:52039624-52125109
TP20829	24	58.88	KB882186.1:672497-732810	14:33619348-33650873
TP33579	24	59.571	KB882260.1:575106-575043	-
TP19723	24	60.083	KB882186.1:185851-185788	5:3834798-3834097
TP66028	24	60.582	-	-
TP45032	24	61.355	KB871800.1:169832-169769	-
TP56154	24	61.863	KB882099.1:1584323-1584386	-
TP35859	24	62.549	KB882099.1:1583891-1606053	-
TP88990	24	63.29	KB882099.1:1083724-1116040	14:25848223-25872579
TP38889	24	63.785	KB882164.1:448244-480211	25:21012807-21020024
TP13493	24	64.325	KB882164.1:100380-100443	-
TP45858	24	65.035	KB882164.1:1039909-1039846	5:39037888-39037930
TP48140	24	65.996	KB882099.1:1330885-1330822	-
TP58857	24	66.93	KB882099.1:1381718-1381781	-
TP87903	24	67.735	KB882099.1:3292379-3292316	14:28319633-28319748
TP8647	24	68.194	KB882108.1:1021818-1021757	-
TP73582	24	68.998	KB882108.1:86728-86665	-
TP19863	24	70.221	KB871759.1:222043-221980	14:28923944-28924316
TP19154	24	72.436	KB882108.1:974073-974010	-
TP2202	24	73.536	KB882186.1:1818803-1818866	-
TP63185	24	78.361	KB882186.1:2060794-2130869	14:35070890-35131017
TP69493	25	0	-	-
TP65100	25	2.263	KB872028.1:137338-137275	-
TP54105	25	5.276	KB871697.1:157442-157379	-
TP64565	25	7.112	KB871697.1:426841-426778	5:38440943-38441310
TP4956	25	8.881	KB871697.1:828234-828297	-
TP63339	25	9.718	KB871697.1:828329-828266	-

TP71807	25	14.705	KB882177.1:2143149-2143212	-
TP56	25	16.476	KB882177.1:1347765-1347702	-
TP46889	25	17.529	KB882177.1:447761-463694	-
TP56902	25	18.384	KB871611.1:701543-719797	-
TP1722	25	19.287	-	-
TP63462	25	20.7	KB882177.1:447761-463694	-
TP90637	25	21.761	KB871611.1:94227-94290	-
TP15653	25	22.257	KB882103.1:3882039-3882102	-
TP9979	25	23.299	KB882210.1:922024-965400	13:23901839-23938370
TP33100	25	24.355	KB871799.1:171637-171700	-
TP91287	25	24.726	KB871611.1:3392-3455	2:59027410-59027447
TP7105	25	25.578	KB882103.1:3882123-3882060	-
TP34562	25	26.442	KB882261.1:902226-902289	13:22219982-22220160
TP84191	25	26.983	KB871799.1:285473-285410	1:50805213-50805486
TP73762	25	27.491	KB882210.1:472116-472053	-
TP218	25	27.897	KB882261.1:160103-160040	-
TP82454	25	28.354	KB882261.1:1036597-1036534	-
TP78864	25	28.982	KB871799.1:336532-336595	3:38716090-38715878
TP33453	25	29.288	KB882210.1:306085-318850	1:44402933-44423718
TP69211	25	30.16	KB871587.1:5657-5595	-
TP65594	25	30.641	KB871587.1:26136-26199	-
TP42037	25	31.299	KB882261.1:460090-460027	-
TP45472	25	32.26	KB871587.1:566916-620305	-
TP56174	25	32.98	KB871587.1:566916-620305	-
TP78338	25	33.303	KB882118.1:478670-478607	-
TP66943	25	33.95	KB882118.1:391488-391425	-
TP9727	25	34.736	KB882118.1:388448-388511	13:53207027-53207190
TP1152	25	35.66	KB871587.1:651520-651583	-
TP10911	25	36.495	KB871587.1:883293-883230	-
TP82929	25	37.472	KB882154.1:2560751-2560814	13:25126030-25125487
TP328	25	38.489	KB882154.1:2101000-2101063	13:25596714-25596670

TP57986	25	39.633	KB882125.1:1985280-1985217	-
TP26891	25	40.384	KB872296.1:3765467-3765404	-
TP72999	25	41.095	KB872296.1:4528841-4528904	9:4652494-4652441
TP78004	25	41.699	KB872296.1:4112593-4112656	-
TP9488	25	43.115	KB872296.1:2158547-2158492	13:6681117-6681042
TP46292	25	43.654	KB872296.1:2059889-2091502	-
TP44580	25	44.707	KB872296.1:1813932-1813869	-
TP83849	25	45.583	KB872296.1:1757994-1757931	-
TP71515	25	46.207	KB872296.1:231316-231253	-
TP44675	25	46.848	KB871915.1:152819-162987	-
TP46276	25	47.629	KB871915.1:274078-284739	-
TP78345	25	48.181	KB871915.1:123351-123414	-
TP85390	25	48.66	KB871915.1:274078-284739	-
TP78349	25	49.335	KB871958.1:237108-237171	-
TP21628	25	50.107	KB872012.1:260529-260592	-
TP65837	25	50.893	KB871652.1:297441-297378	-
TP52806	25	51.578	KB871652.1:865123-896963	13:12902994-12968957
TP68218	25	53.141	KB871652.1:286088-286025	-
TP11149	25	53.735	KB882206.1:16071-27995	-
TP80197	25	55.336	KB871819.1:479347-479410	-
TP15076	25	56.131	KB872601.1:34399-34462	-
TP7661	25	56.924	KB882206.1:1057648-1057585	-
TP64329	25	57.471	KB882206.1:1446391-1471522	-
TP56952	25	58.313	KB882206.1:1476132-1476069	-
TP52868	25	59.197	KB882206.1:1446391-1471522	-
TP42639	25	60.071	KB882206.1:1443690-1443627	-
TP53891	25	61.059	KB871838.1:6978-7041	13:33142708-33143265
TP1639	25	61.632	KB871838.1:7052-6989	13:33142708-33143265
TP72005	25	62.57	KB882107.1:2892531-2903032	13:3593110-3605257
TP20960	25	63.762	KB872209.1:47690-47627	-
TP67639	25	64.637	KB882107.1:2535003-2535066	-

TP56436	25	65.63	KB882107.1:2129380-2129317	-
TP51255	25	66.358	KB882107.1:1788123-1788186	13:5285891-5285930
TP1242	25	66.781	KB882107.1:1788229-1788166	13:5285891-5285930
TP91880	25	67.787	KB872170.1:187345-187408	-
TP26109	25	68.377	KB871960.1:66137-66074	1:28632100-28632131
TP27475	25	69.349	KB871591.1:27352-122773	13:48083531-48150481
TP80492	25	69.993	KB882107.1:161570-304981	13:1355889-1594370
TP20629	25	70.444	KB882109.1:3506678-3506741	-
TP32151	25	71.067	KB872182.1:201997-201934	19:19146832-19146890
TP56264	25	71.866	KB872182.1:201927-201990	19:19146832-19146890
TP57860	25	72.742	KB872182.1:202355-202418	19:19146832-19146890
TP40248	25	74.125	KB871591.1:172-235	-
TP89219	25	74.918	KB882107.1:642611-642674	-
TP72281	25	75.352	KB871591.1:754902-754965	11:44610380-44610219
TP85399	25	76.991	KB882109.1:2502021-2502084	13:13343651-13343388
TP41723	25	77.569	KB876325.1:6975-6912	-
TP51188	25	78.627	KB871598.1:1102242-1102182	-
TP11292	25	79.962	-	-
TP79950	25	81.143	KB872081.1:536313-536376	-
TP55442	25	81.964	KB876308.1:1933-1870	-
TP18603	25	82.525	KB882109.1:814065-814128	9:1563496-1563377
TP32191	25	83.382	KB882291.1:1055847-1055910	15:37757752-37757692
TP90730	25	84.139	KB871598.1:1102185-1102122	-
TP3975	25	85.322	KB872081.1:536384-536321	-
TP16108	25	86.031	KB872081.1:4441715-4468548	13:46144663-46190695
TP47550	25	86.967	KB882291.1:946474-952273	-
TP54270	25	87.849	KB872081.1:5040460-5040523	10:43100718-43100766
TP62703	25	89.07	KB872081.1:4053479-4053416	-
TP82166	25	90.324	KB872081.1:3795943-3796006	13:4080649-4080749
TP68688	25	91.636	KB872081.1:3999967-3999904	-
TP78620	25	94.407	KB872081.1:3249952-3249889	1:54337131-54337356

TP42775	25	96.973	KB872081.1:3999890-3999953	-
TP63232	25	97.594	KB872081.1:2106852-2115142	-
TP11584	25	98.514	KB872081.1:1050496-1050559	-
TP35766	25	100.933	KB872081.1:1050571-1050508	-
TP25865	25	104.982	KB872081.1:2106852-2115142	-

[a]In instances where a transcript-based result was chosen, positions in *Astyanax* are reflective of the span of the entire gene, not just the region with which our 64-bp GBS marker sequence aligned.

[b]Listed positions in *Danio* reflect the span of the alignment between *Danio* and the searched *Astyanax* sequence (e.g., an entire gene transcript or ~2000-bp genomic sequence harboring a GBS marker sequence).
